# Cost-effective and power-efficient portable turbine-based emergency ventilator

**DOI:** 10.1016/j.ohx.2022.e00350

**Published:** 2022-08-26

**Authors:** Syed Razwanul Haque, Shovon Sudan Saha, A.K.M. Maruf Hossain, Md Hasanur Rahman Sohag, Fozle Rabbi Shafi, Satya Ranjan Sarkar, Tanzilur Rahman

**Affiliations:** a CRUX Technologies, Sylhet, Bangladesh; b University of Waterloo, Ontario, Canada; c Oishi Electronics, Sylhet, Bangladesh; d North South University, Dhaka, Bangladesh

**Keywords:** Biomedical, Emergency, Ventilator, Turbine-based, Open-source, Low cost, Modular, Portable, Covid-19, Ards, OSHWA, Sfm3300, Mpx2010, Prvc, Simv, Peep, 3d printing, Pressure release mechanism

## Abstract

Ventilators have always been common in medical scenarios but are very expensive to procure or develop. One of the main reasons for these is the components that are being used are expensive and require precise instrumentation, research, and development. This paper attempts to mitigate that problem by proposing a novel way to rapidly develop a portable ventilator that uses common 3D printing technology and off-the-shelf components. This turbine and valve-based ventilator feature most of the modes that are commonly used by healthcare professionals. A unique servo-based pressure release mechanism has been designed that makes the system around 36 times more efficient than solenoid-based systems. Reliability and efficiency have been increased further through the use of a novel positive end-expiratory pressure (PEEP) valve that does not contain any electromechanical component. Effective algorithms such as feed-forward and proportional–integral–derivative (PID) controllers were used alongside the unique ‘Sensor data filtration methodology’. The system also provides an interactive graphical user interface (GUI) via an android application that can be installed on any readily found tabs while the firmware manages the breathing detection algorithm using a flow meter and pressure sensor. This modular and portable ventilator also features a replaceable battery and holds the ability to run on solar power. This energy-efficient low-noise system can run for 5 to 6 h at a stretch without needing to be connected to the main’s supply.

## Graphical abstract

**Figure:**
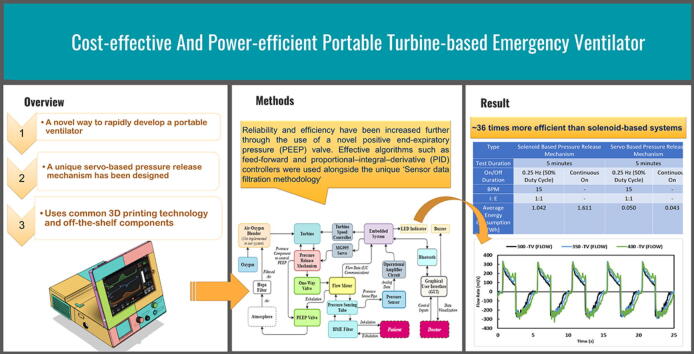

Specifications table.

**Table:** 

Hardware name	Portable turbine-based emergency ventilator
Subject area	Medical
Hardware type	• Biomedical device Sensors Electronics Pneumatic system
Closest commercial analog	Portable ventilator
Open-Source License	CC BY SA 4.0
Cost of Hardware	*$*284
Source File Repository	https://doi.org/10.17632/b94xgzxsdm.2
OSHWA Certification UID	BD000001

## Hardware in context

Medical ventilators are one of the most integral parts of an intensive-care unit’s setup. However, their significance became very apparent to the mass people when Covid-19 struck the world leading to cases of acute respiratory distress syndrome (ARDS) condition escalate. Even though ventilator manufacturing companies immediately tried to ramp up their production, it was insufficient. Due to the complexity of the development process and supply chain, not much could have been possible. Besides that, along with the cost of the ventilators, the procurement process is also not easy. On top of that, most of the devices found on the market have proprietary parts and complicated operations requiring skilled personnel.

## Hardware description.

A low-cost portable emergency ventilator that is rapidly developable using readily available components while featuring the prominent modes used by healthcare professionals.

Highlighting some of the advantages of the proposed system below:

• Low-cost and robust:

The development cost is affordable as it uses commonly available technology, such as 3D printing. Moreover, most of the design components are widely available on the market and can also be taken from other devices such as drones, other respiratory devices, etc.

• Portable with long battery backup:

Over recent years, there have been considerable developments in portable ventilators, however, they are still very bulky with low battery life. Weighing only 3.7 kg, the proposed system can run for 5.6 h at 10 breaths per minute (BPM), 2 s inspiration time , and at 35 cm·H_2_O pressure. The device is found to be consuming only about 16 W, which enables it to operate relentlessly for long hours. Swappable and modular battery systems empower the system’s operation time to be increased further. Furthermore, the design holds extra space to add more batteries; enabling the option to increase the runtime further.

High efficiency and low noise (26.1 dB from 1 m distance) have been achieved through the novel design of our pressure release mechanism (PRM) and positive-end-expiratory-pressure (PEEP) valves. A servo-based mechanism has been used that can easily replace the heavy power-consuming solenoid-based mechanisms that are to be found in any ventilators. More than 36 times more efficiency can be achieved using such a system. This comes to a great advantage along with portability.

This mechanism can also be used in various pneumatic systems such as compressors, safety valves, oxygen concentrators, and fuel control systems.

Nevertheless, this ventilator can also be used as a continuous positive airway pressure (CPAP) therapy device to help a person during sleep who struggles due to obstructive sleep apnea (OSA) [1]. Due to its easy operation, it can also be used at the home.

• Adaptable to any patient circuit:

Upon effective market analysis and looking deep into some commercially available ventilators, it was realized that the patient circuits that were used are always unique and exclusive to that particular brand. This becomes a huge problem even after the hospital procures a ventilator because the entire ventilator becomes unusable even if just the patient circuit is not available. To avoid such a massive problem, the system had been designed to adapt to any patient circuit available on the market. Besides that, the pressure sensor (MPX2010DP) [2] which can measure 0 to 101 cmH2O pressure and flow sensor used in this system can easily be replaced with other available sensors on the market; increasing the adaptability of this system.

• Graphical User Interface (GUI) is adaptable to any android device:

An android application is developed that communicates with the system through a predetermined data frame using Bluetooth. The low size and efficient software architecture enable the app to be highly responsive and require small storage space, subsequently allowing the app to be installed on just about any device with an android operating system.

Nevertheless, it can also be connected to single-board computers such as Raspberry Pi [3], and Radxa Zero [4].

• Ability to power itself using solar panels and other alternate resources:

Unfortunately, still in a lot of lower- and middle-income countries (LMIC), there are many regions where power through the grid has not been able to reach and this acts as a huge barrier to using biomedical devices in the clinics and hospitals that are barely available. To diminish such problems the portable ventilator can be charged using solar energy, wind energy, etc. where a reliable constant power source is not available. Moreover, it also holds the potential to be deployed at refugee camps and disaster-affected areas to provide critical care to the victims as they cannot be relocated due to disruption in transportation and logistics.

This paper proposes building a ventilator that can easily be developed using low-cost 3D printing technology (fused deposition modeling) [5], readily available materials for assembly, and sensors that need not be coded from scratch to be used. This can mitigate the problem of gaining access to ventilators as they can be developed at a very low cost compared to ventilators that are found on the market.

**Fig. 1:**
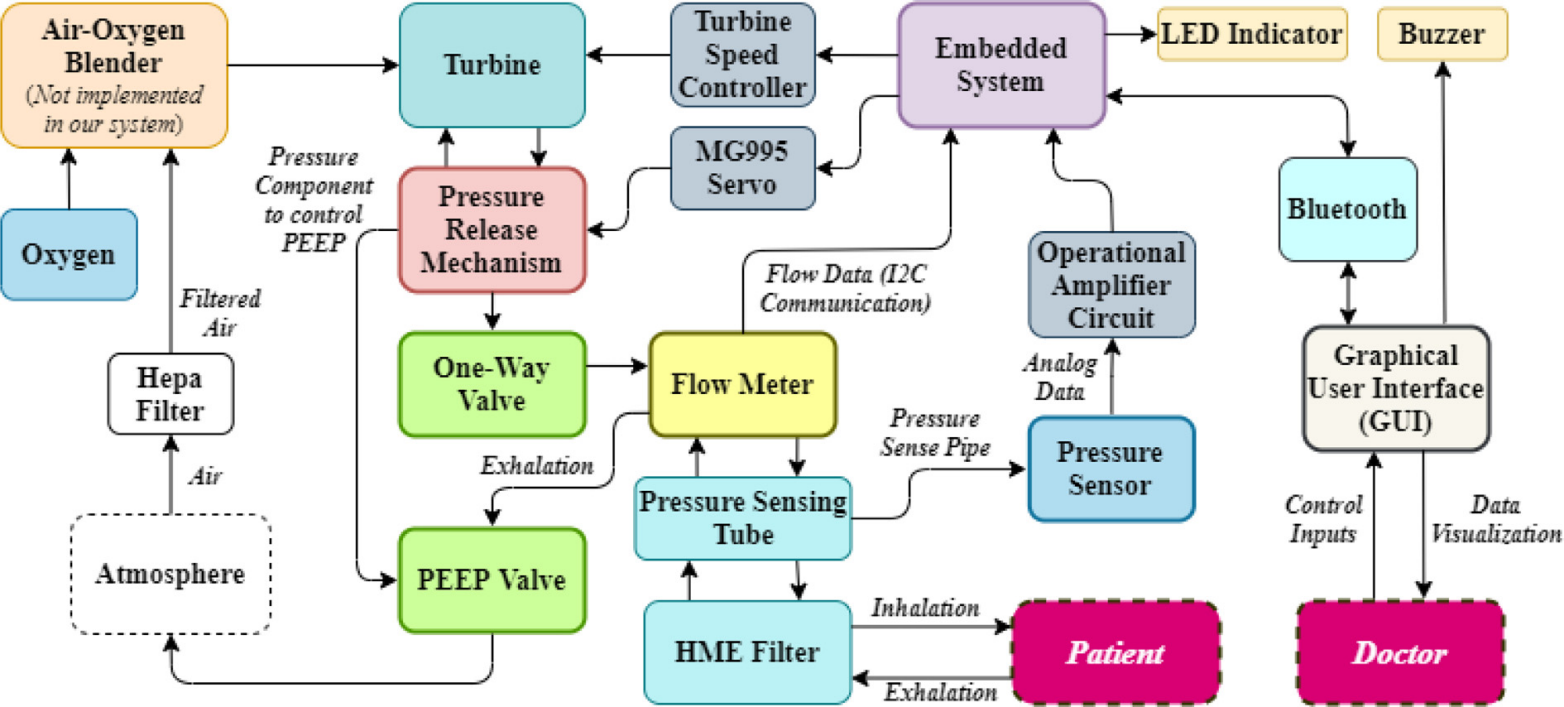
System Architecture for our turbine-based ventilator.

The system architecture [Fig. 1] demonstrates the airflow to and from the atmosphere to the patient passing through the critical modules of the system. The first stage of filtration is conducted by using a HEPA filter to trap Covid-19 particles. The filtered air is then fed into the pressure release mechanism leading to the one-way valve and then to the flow meter, eventually passing through the pressure sensing pipe and a second filtration through the Heat and Moisture Exchanger (HME) filter [6] before being inhaled by a patient.

During the exhalation period [Fig. 1], exhaled air filters from the HME filter and travels from the patient to the PEEP valve after the flow meter, subsequently leading back to the atmosphere.

The turbine and pressure release mechanism is controlled by the turbine speed controller and servo respectively by the embedded system. The embedded system takes in data from the system through a flow meter and pressure sensor to run in the set settings by a healthcare professional using the graphical user interface (GUI) that sends data to and from the embedded system to send settings and represent data.

### Mechanical

(a) Turbine:

After testing out several turbines by 3D printing, it was decided to use a readily available turbine. 7040 DC 12 V centrifugal turbo turbine [7] was chosen because of its ability to provide high airflow of 240 L/min, 70 cmH2O air pressure, low noise, low rise in temperature during operation, and compact size. Moreover, it also has a built-in control circuit that made controlling the airflow of the entire system much easier. Lastly, the three-phase brushless motor enabled the ventilator to run at very low power.

**Fig. 2:**
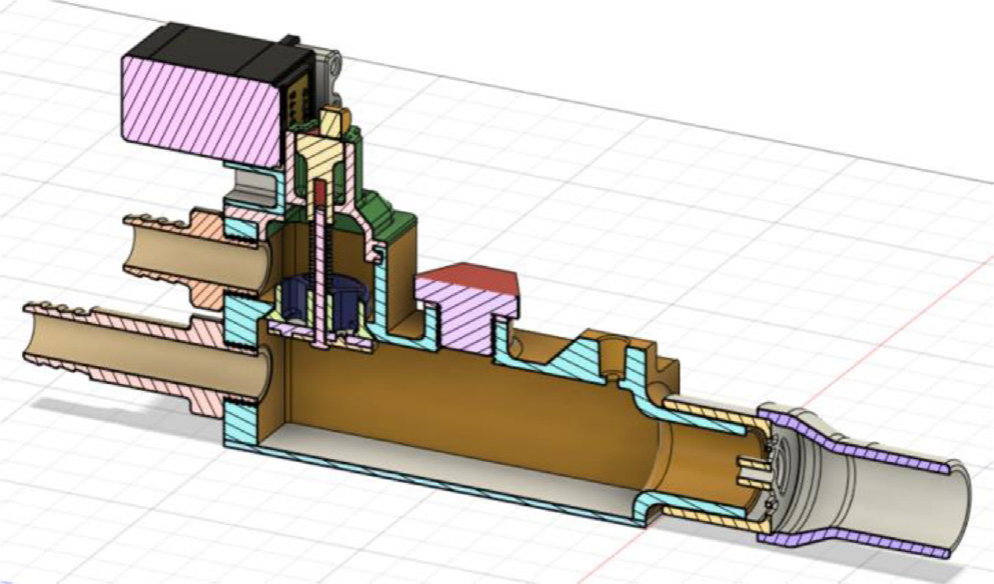
Cross-section of the PRM.

(b) Pressure Release Mechanism (PRM):

An efficient servo-actuated system was developed for the PRM. This enabled the system to regulate the pressures quickly during the inhale and exhale cycles. It also played a vital role in deviating the pressure while the turbine is on and providing a synchronization control of the positive end-expiratory pressure (PEEP) valve. The cross-section of the PRM is shown where all the parts are visible in Fig. 2.

**Fig. 3:**
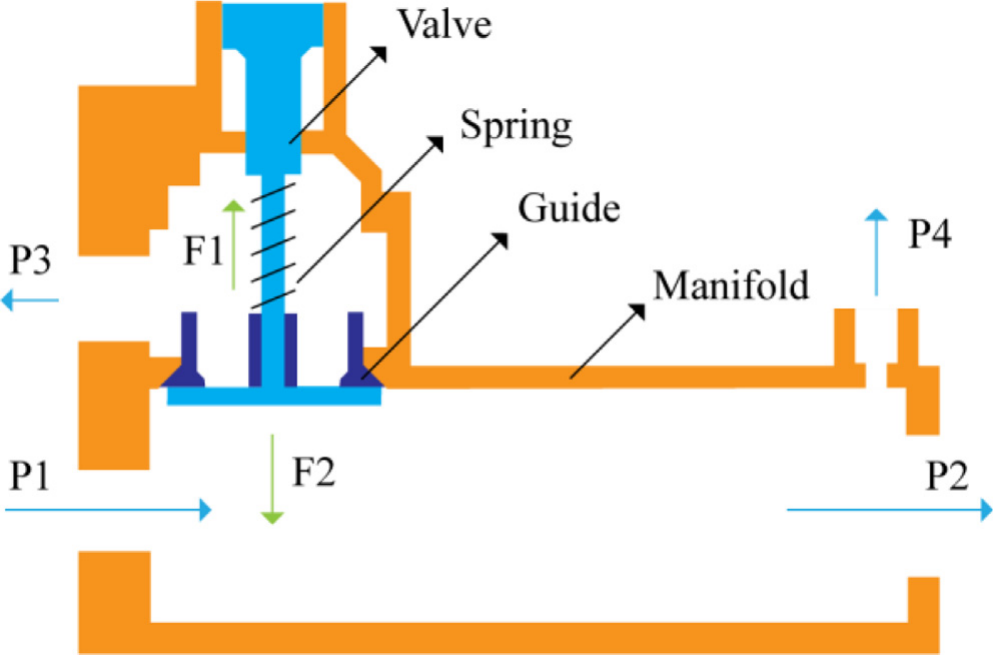
Free-body diagram of PRM during inhale state.

In Fig. 3, the PRM is in the inhale state. The manifold, valve, and guide of the PRM are all to be 3D-printed to be easily deployable and fit into the system. The guide was glued onto the manifold that provides a supported pathway for the valve to move. A spring was installed to keep the valve pushed upwards so that during the inhale state, air can flow from the inlet. The magnitude of the pressures depends on Eq.(1). *P*1 pressure is provided at the inlet. Since *P*3 pressure is zero, *P*1 is divided into *P*2 and *P*4. *P*2 and *P*4 are going to the patient and the pressure component is applied to the PEEP valve respectively.

(1) P1(cmH2O)=P2(cmH2O)+P3(cmH2O)+P4(cmH2O)

**Fig. 4:**
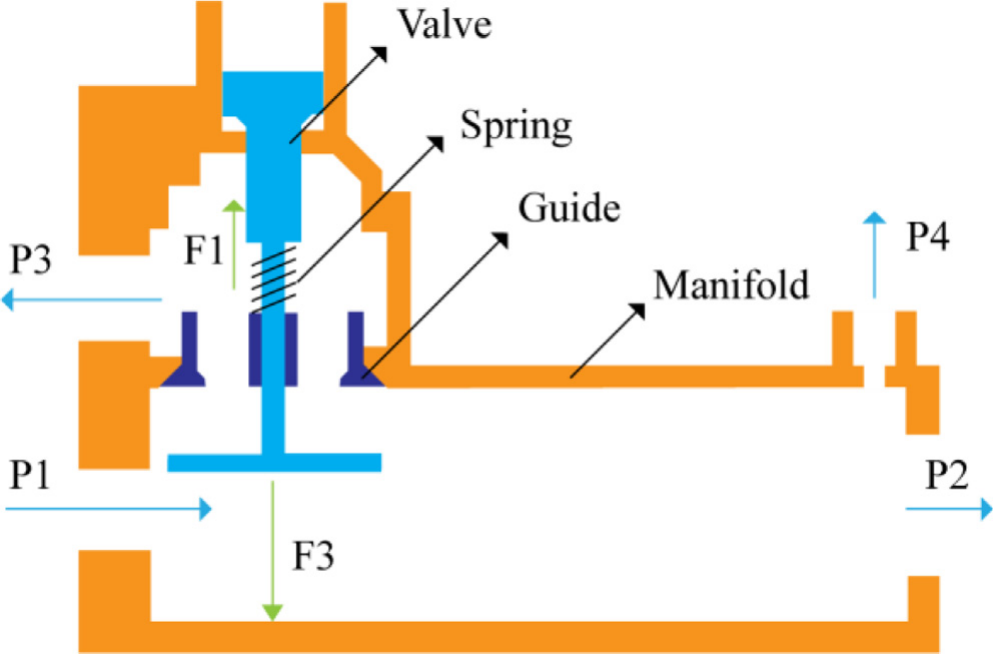
Free-body diagram of PRM during exhale state.

During the exhale state (Fig. 4), the horn of the servo pushes the valve downwards, compressing the spring and opening up the space between the guide and the manifold. A large portion of the pressure now deviates through that gap and raises the pressure *P*3, eventually decreasing the pressure *P*2 and *P*4. These pressures play a vital role in the system to keep a positive PEEP to restrict the alveoli from collapsing. When the system enters the inhale state, the servo again retains its original position and the valve again closes itself due to the force (F1).

**Fig. 5:**
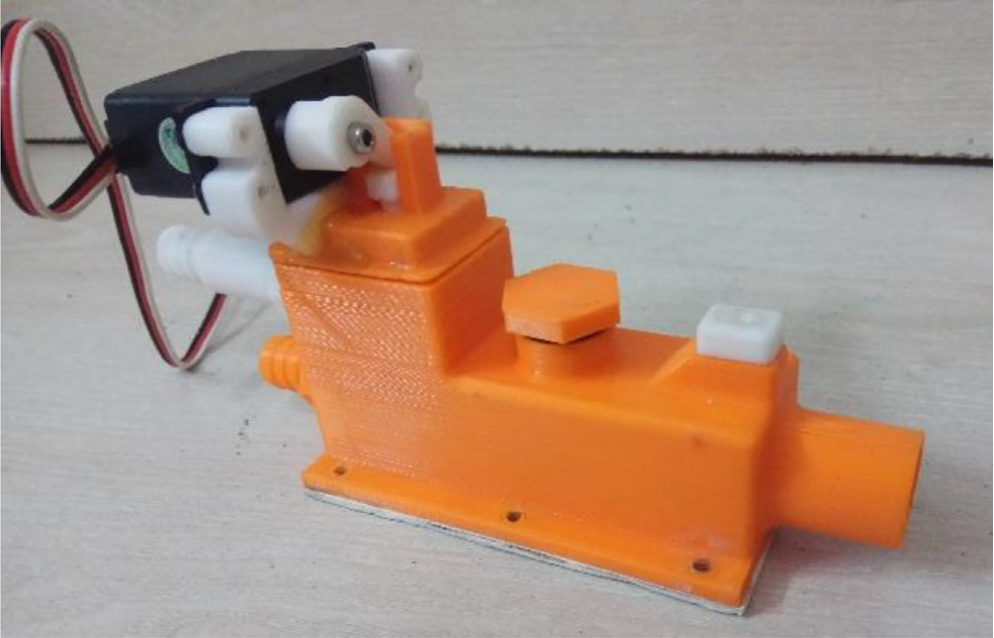
Pressure Release Mechanism with MG995.

To summarize, this novel design of the pressure release mechanism is power efficient and minimizes failure as it is normally open. The PEEP is adjusted by maintaining the speed of the turbine. The air travels from the turbine through this mechanism and then reaches the patient after passing the ‘one-way valve’. Fig. 5 demonstrates the PRM valve after assembly.

**Fig. 6:**
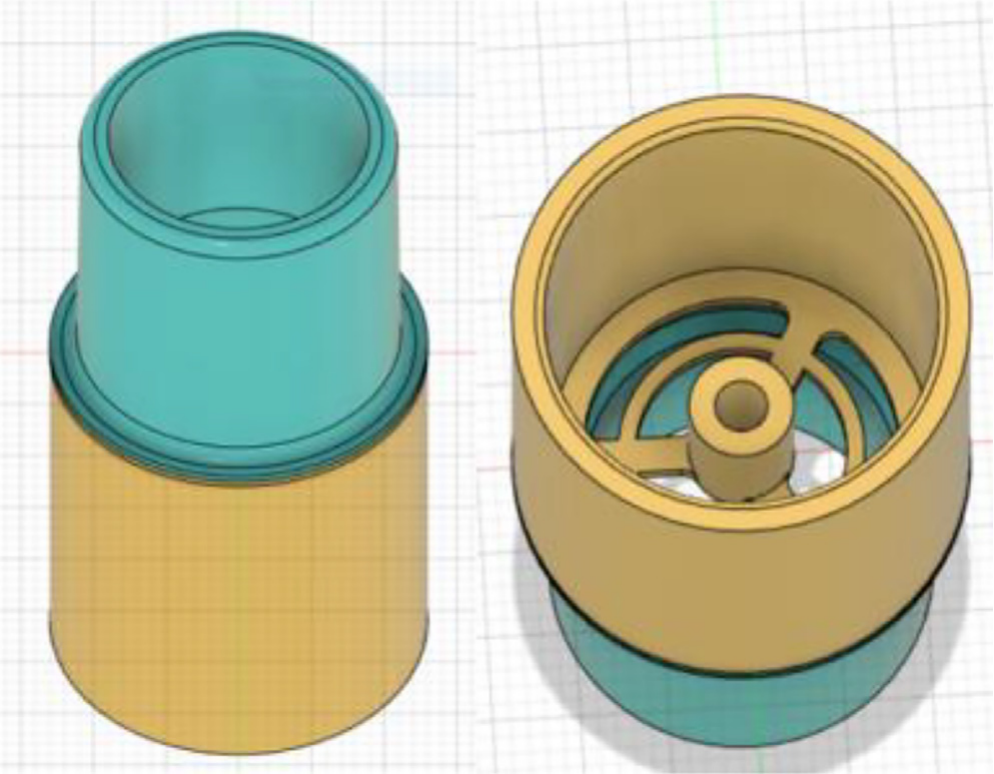
One-way valve.

**Fig. 7:**
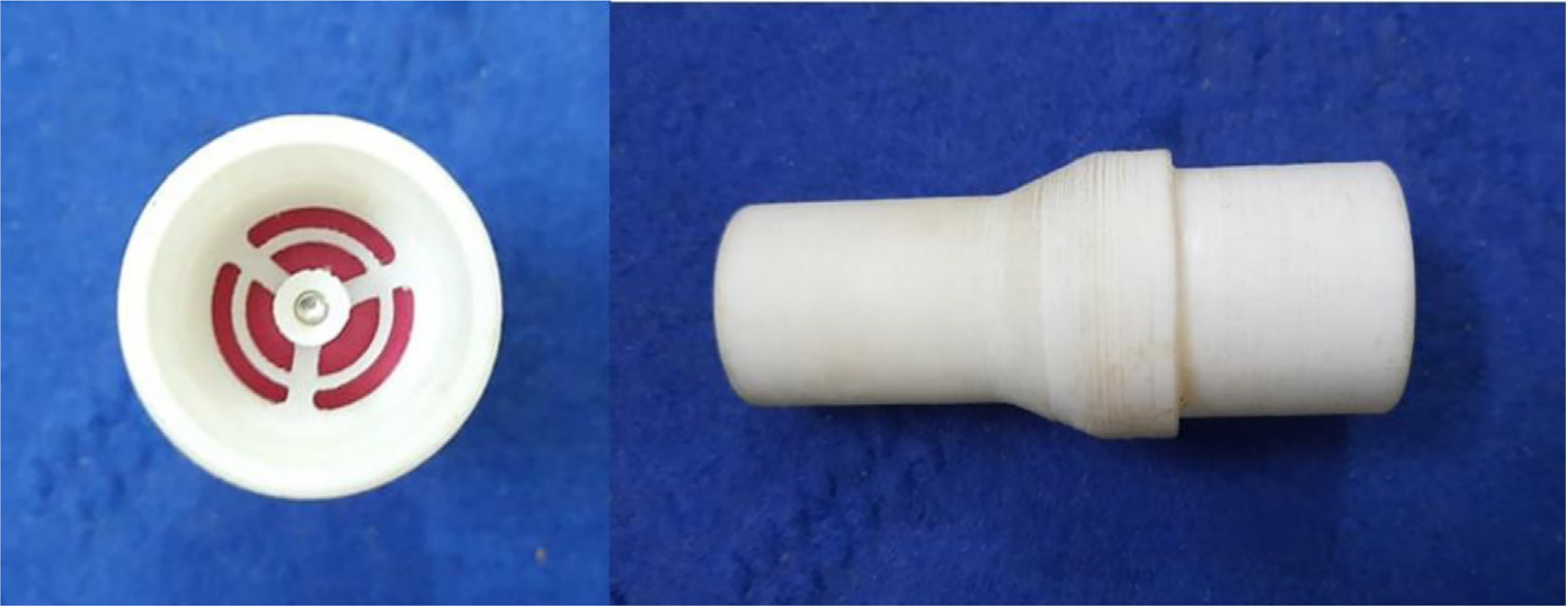
One-way valve (real-life).
(d) **Positive End-Expiratory Pressure (PEEP) valve:** Maintaining the PEEP value ranging from 5 to 20 cmH_2_O of patients is highly essential so that the alveoli of the patients do not collapse and there is some volume present inside, which makes it easier to inflate; this point of change in compliance that is denoted as the critical opening pressure (COP) [8].

(c) **One-way valve:** was custom designed by our team to fit as appropriate modules were not available and procuring the exact module would have taken a long time. It was experimented with several times before a design (Fig. 6 and Fig. 7] was finalized that worked brilliantly with 5 cmH_2_O. The 3D-printed structure holds a rubber membrane on the inside and can fit any readily available patient circuit.

**Fig. 8:**
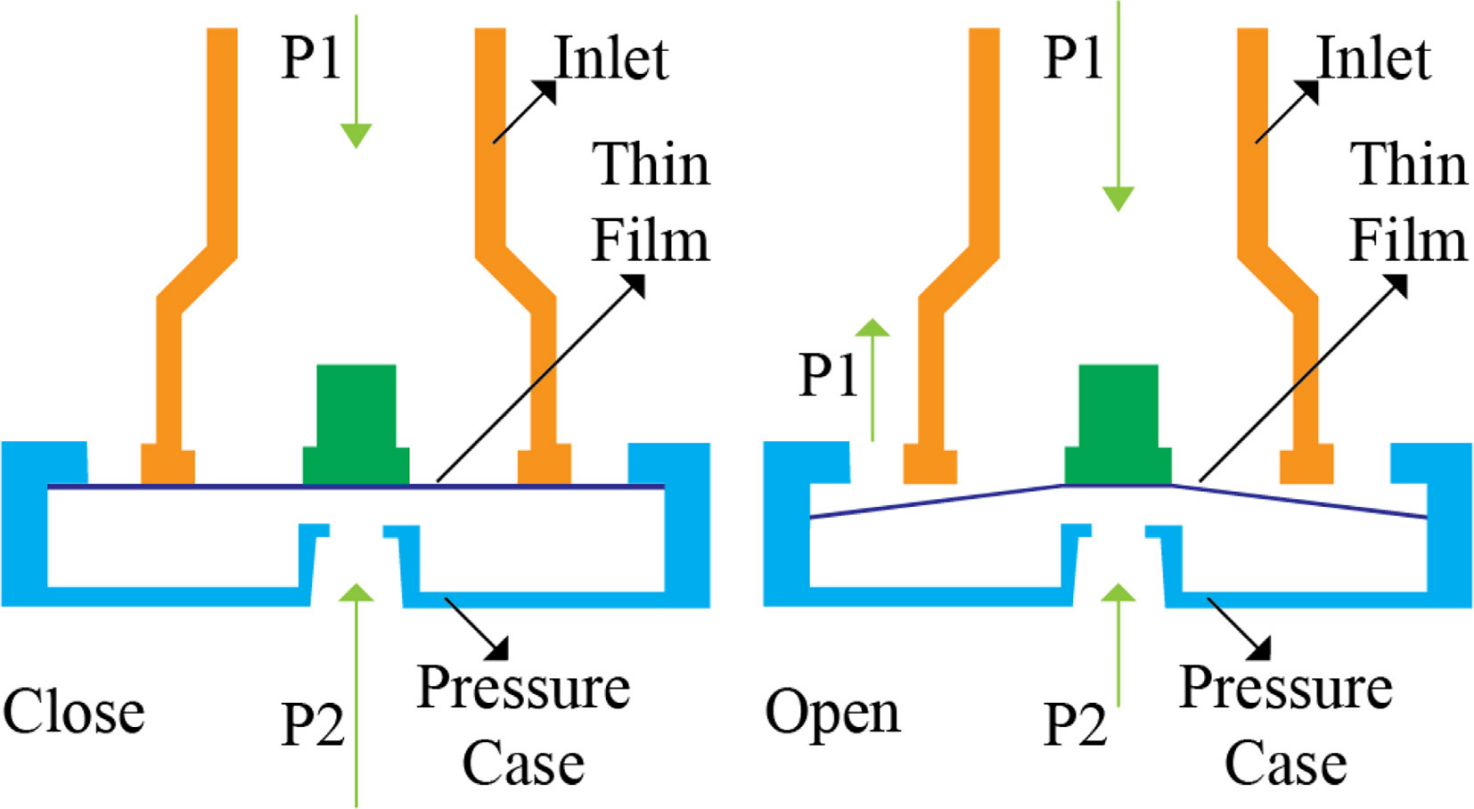
Free-body diagram of the PEEP valve.

Fig. 8 shows the free-body diagram of the PEEP valve during both the inhale and exhale states to the left and right respectively. Both the pressure case and inlets are 3D printed using PLA and a thin film made up of silicone sheet is added in between both the parts. During the inhale state, high pressure is exerted (*P*2) through the pressure component taken from the PRM and the thin film is pushed tightly towards the inlet as there is a low pressure (*P*1) coming from the patient. However, quite the opposite happens when the patient is in an exhale state and the pressure *P*1 now is much higher than that of *P*2, leading to pushing down the film and letting the air out.

**Fig. 9:**
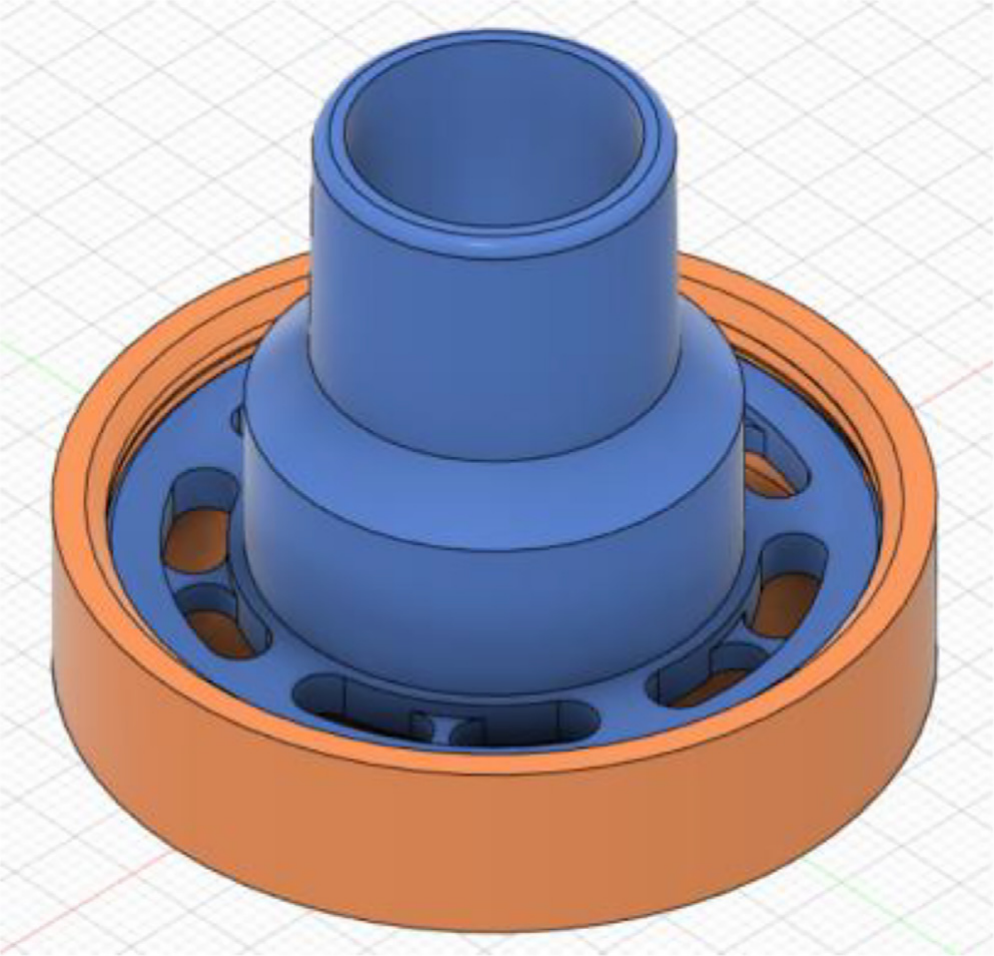
Tailored PEEP valve.

This novel design and mechanism of the PEEP valve (Fig. 7) operate without the aid of any electromechanical components and this enhances the reliability of the entire system. In the scenario of failure, the valve would just open and avoid suffocation of the patient. Moreover, designing the control algorithm becomes easier as only one variable; the speed of the motor needs to be regulated.

**Fig. 10:**
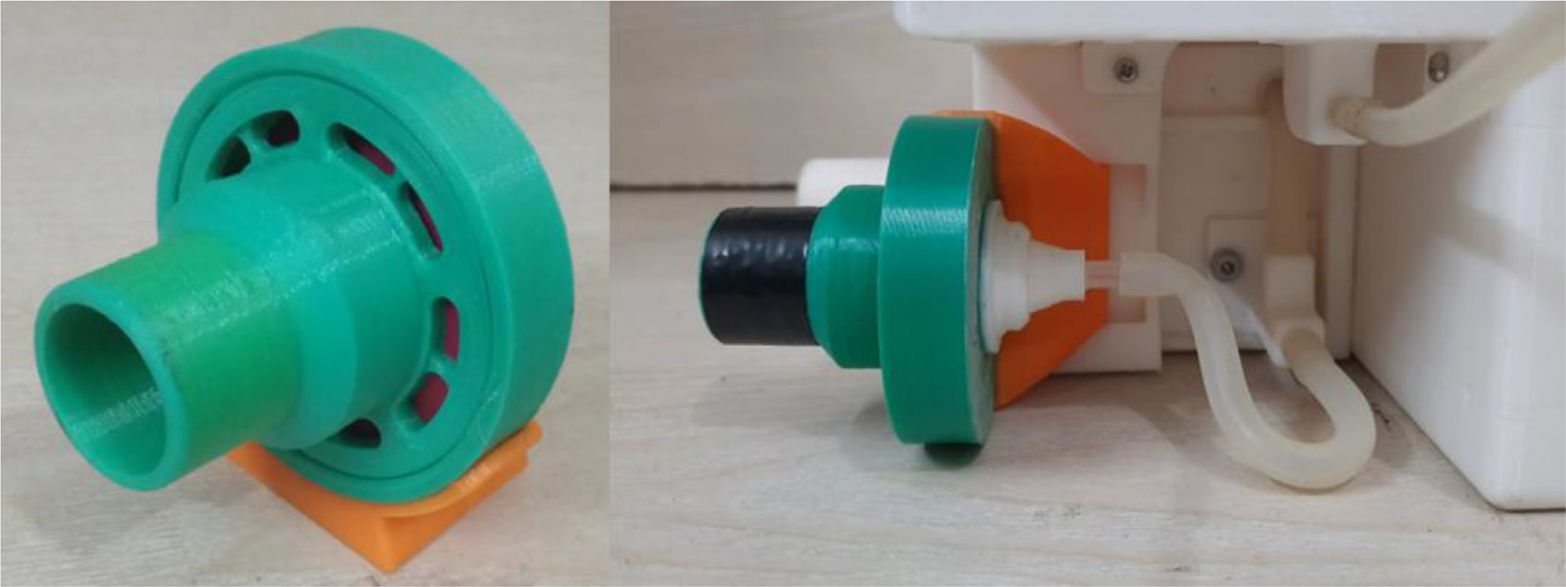
PEEP valve after assembly.

The complication of the supply chain is reduced due to the use of a lower number of parts; eventually reducing the cost of production. Nevertheless, this design decreases the time taken for assembly. Fig. 10 shows the final assembly of the valve.

### Electrical and electronics

**Fig. 11:**
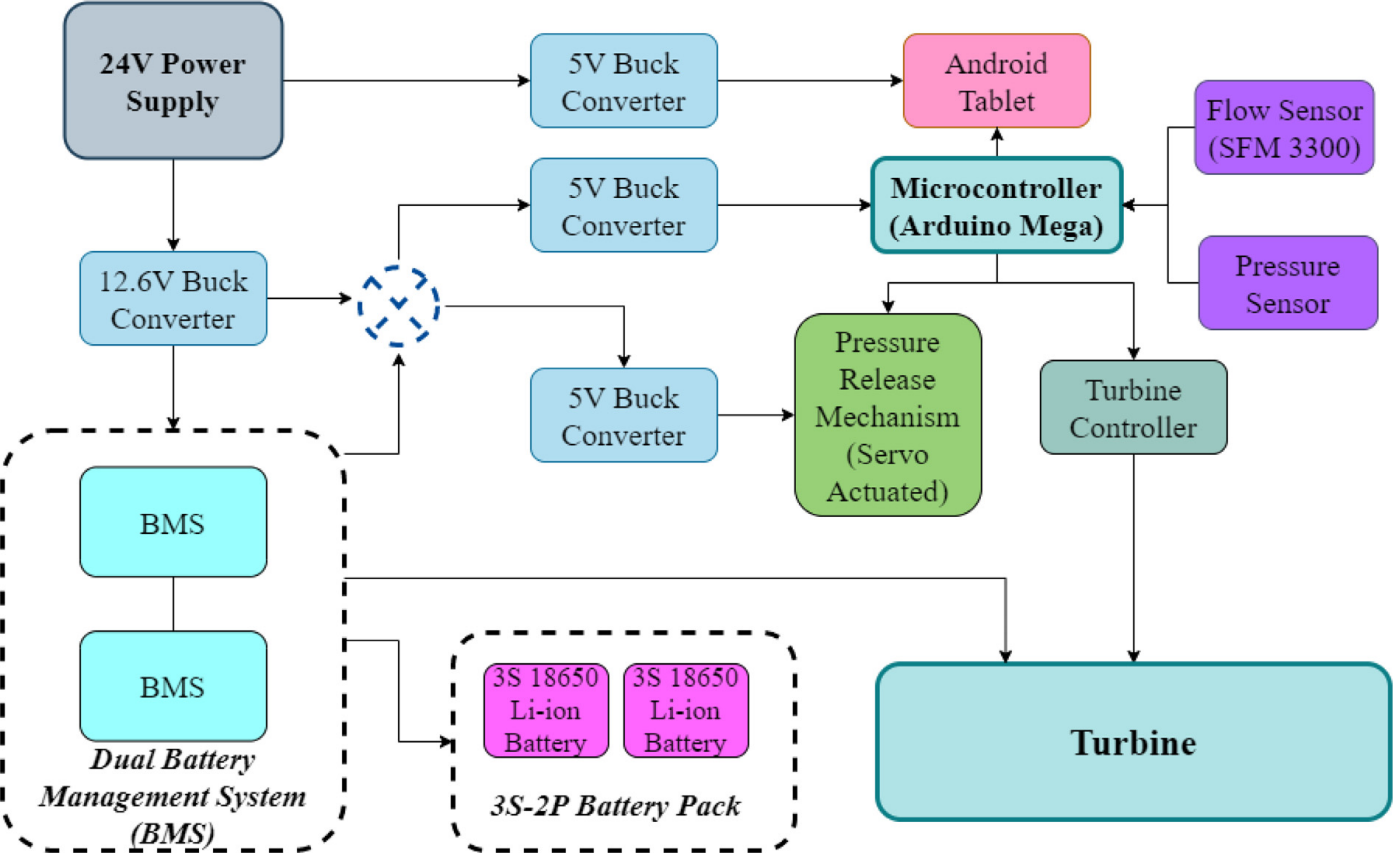
Electronics Modules’ Integration.

To run the mechanical system seamlessly a robust circuit was integrated with the electronic modules (Fig. 11). Generic modules were chosen in the development because these were abundantly available in the market, which would eventually make the development process and supply chain much less complicated if it enters the production phase.

A 24 V power supply was used to drive and charge the system when connected with the mains power. One 12.6 V buck converter (LM2596) was used to power the battery management systems (BMS), two are used in parallel to charge two 3S 18,650Li-ion battery packs.

Three separate 5 V buck converters (XL4015) have been used to power the microcontroller (Arduino Mega), android tab, and the servo. This isolation of converters increases the sturdiness of the device. Two low-powered sensors such as the flow sensor (SFM3300) [9] and differential pressure sensor (MPX2010DP) [2] feed analog data into the Arduino.

**Fig. 12:**
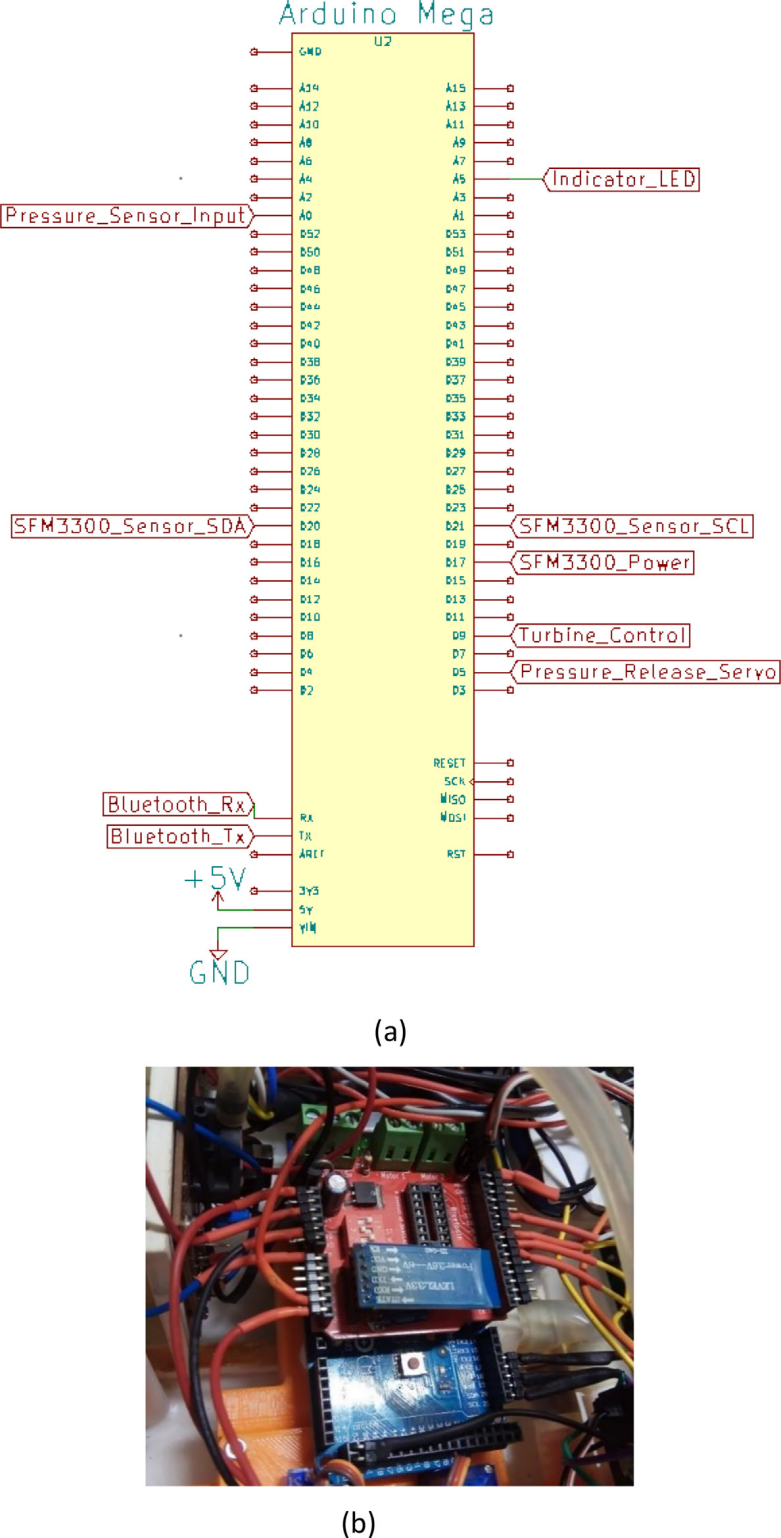
(a) Embedded system’s circuit schematic with the nodes labeled to be connected at respective places and (b) picture of the embedded system connected in the device.

**Fig. 13:**
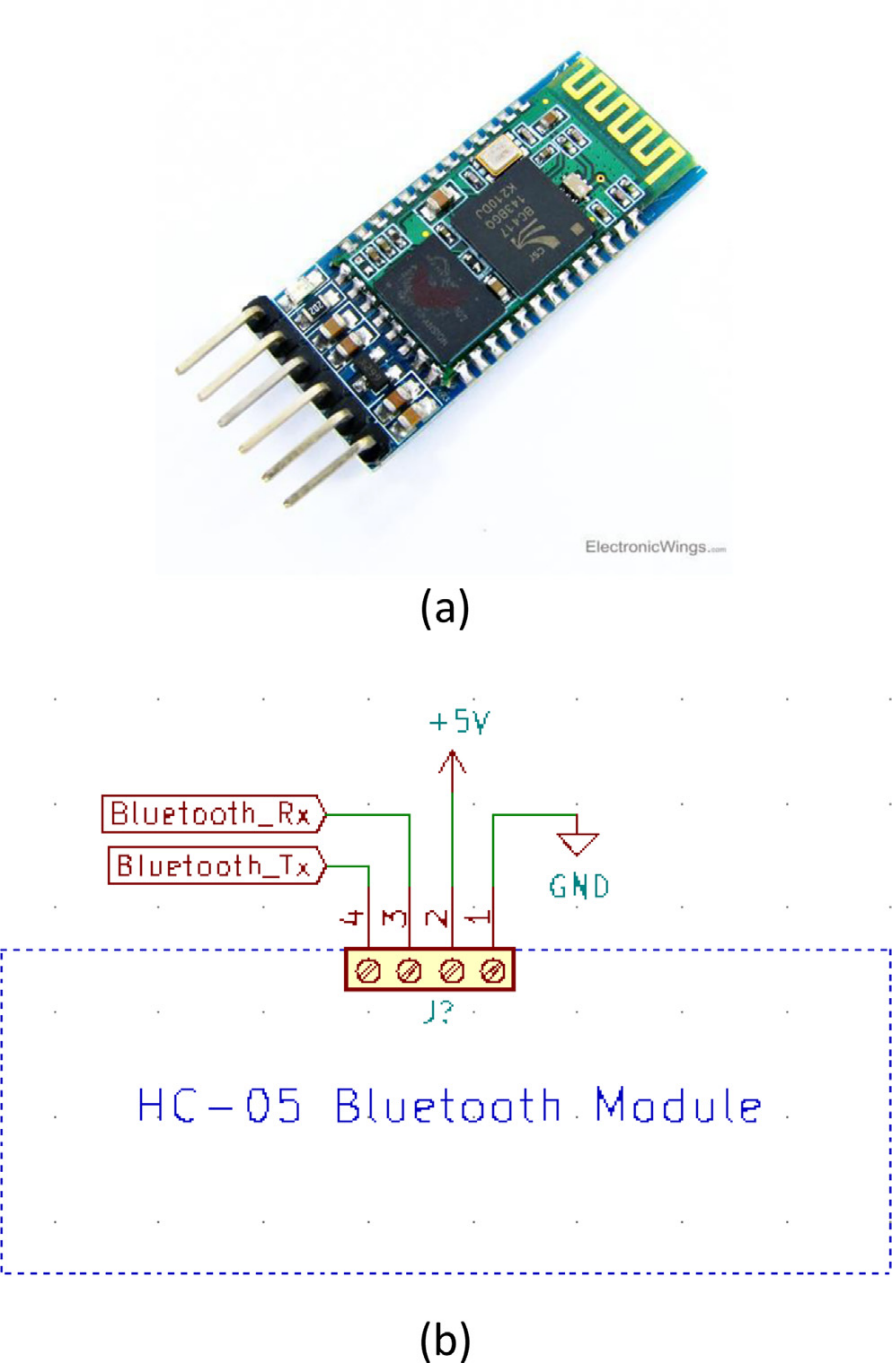
(a) HC-05 Bluetooth module [11] and (b) schematic of the module.

(a) Key modules.
  (i) Microcontroller:

Arduino Mega [10] is being used as the processing unit of the device for its wide availability, robustness, and number of digital pins available. It acts as the brain of the system taking in data from all of the sensors that are connected and producing appropriate control signals to control the turbine and servo. Besides that, data transmission through Bluetooth is also conducted by this microcontroller (Fig. 12).

(ii) Bluetooth module:

The user inputs are sent from the Android application to the microcontroller via a Bluetooth module (HC-05) [11] (Fig. 13a), which are then inputted into different control algorithms and other processes. Our app handled the serial communication seamlessly without any failure at a baud rate of 9600 where the predetermined data frame comprising of multi-variant values was lined up in single strings. After that string is segmented based on the preset data length of each variable and portrayed on the necessary graphs which could optimize its scale and provid better visibility to perceive useful information. One can also directly connect the device with our ventilator through the USB port. The schematic of the turbine controller is demonstrated in Fig. 13b.

(iii) Servo:

It’s mandatory to increase and decrease pressure during inhale and exhale respectively in a fast manner and to serve that very purpose, a servo-actuated system using MG995; operating at 5 V and having a stall torque of 9.79 kg/cm [12] was designed. Rising the pressure very quickly at the targeted value was achieved by maintaining the turbine’s speed to a minimum, so that, it does not have to start from zero.

**Fig. 14:**
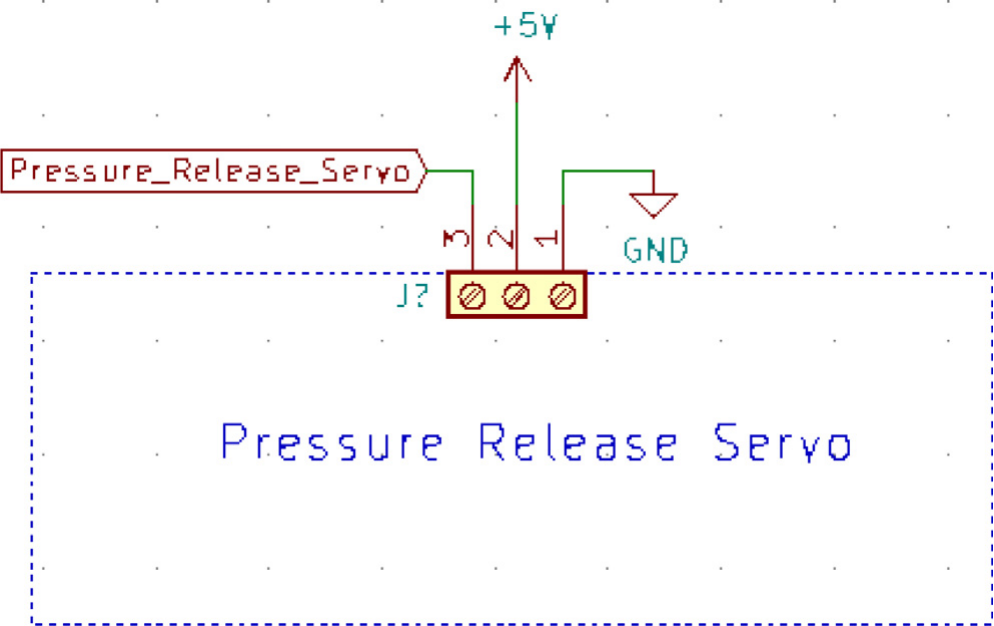
Pressure release servo schematic.

One of the key attributes for our system to be efficient is due to our servo-based pressure release mechanism’s low power requirement. Upon experimentation on the certain conditions; explained in Table 1, we found that in 50 % duty cycle mode and continuous mode the energy consumption of our servo-based system is 19.84 times and 36.46 times more efficient. The schematic of the turbine controller is demonstrated in the Fig. 14.

(iv) Turbine controller:

**Table 1:** Power comparison of Pressure Release Mechanisms.

Type	Solenoid Based Pressure Release Mechanism		Servo Based Pressure Release Mechanism	
Test Duration	5 min		5 min	
On/Off Duration	0.25 Hz (50 % Duty Cycle)	Continuous On	0.25 Hz (50 % Duty Cycle)	Continuous On
BPM	15	–	15	–
I: E	1:1	–	1:1	–
Average Energy Consumption (Wh)	1.042	1.611	0.050	0.043

**Fig. 15:**
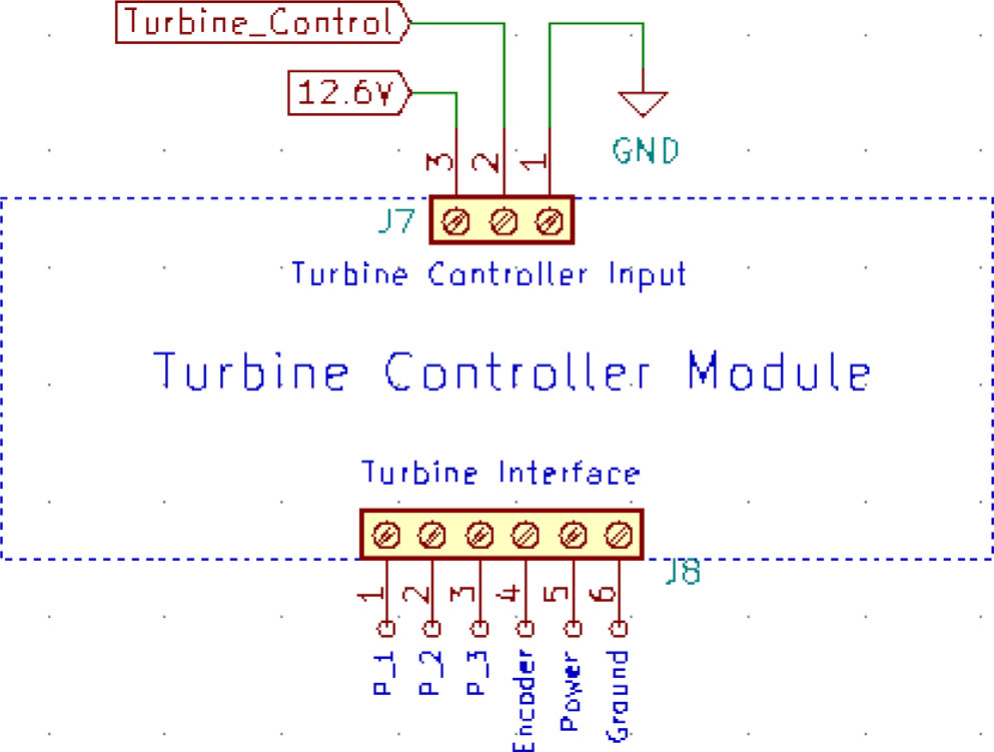
Turbine controller schematic.

The previously mentioned turbine comes with an integral turbine controller that can easily be used to control the PWM signals from the micro-controller. This is more like an electronic speed controller (ESC) with extended functionalities that remove all the complexity that is often present in controlling a motor/turbine. Hence, easy integration of the control algorithms is possible. The schematic of the turbine controller is demonstrated in Fig. 15 below.

(a) Sensors.
  (i) MPX2010DP:

MPX2010DP is a differential pressure sensor [2] that takes in data from the pressure sense pipe mentioned in Fig. 1 and feeds analog data into the Arduino for the firmware to work. However, the full-scale span of the pressure sensor is 2 mV, so it needs to be amplified (Fig. 9) before sending it to Arduino and eventually converting it to a cmH2O pressure unit using Eq.(2). The equation can be found in [13].

**Fig. 16:**
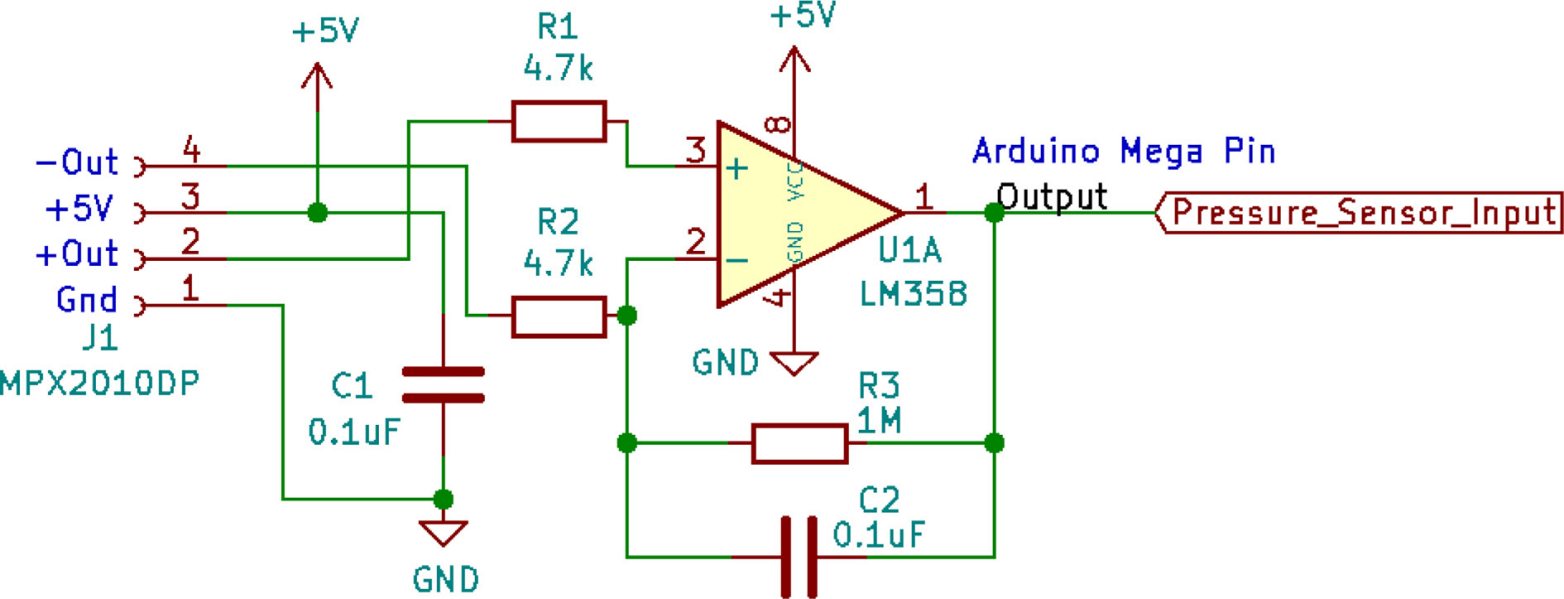
Amplifier (LM358) circuit schematic for MPX2010DP pressure sensor.

An operational amplifier (LM358) is used for sensor signal amplification. Fig. 16 demonstrates the circuit used. It uses a very generic yet effective circuit.

**Fig. 17:**
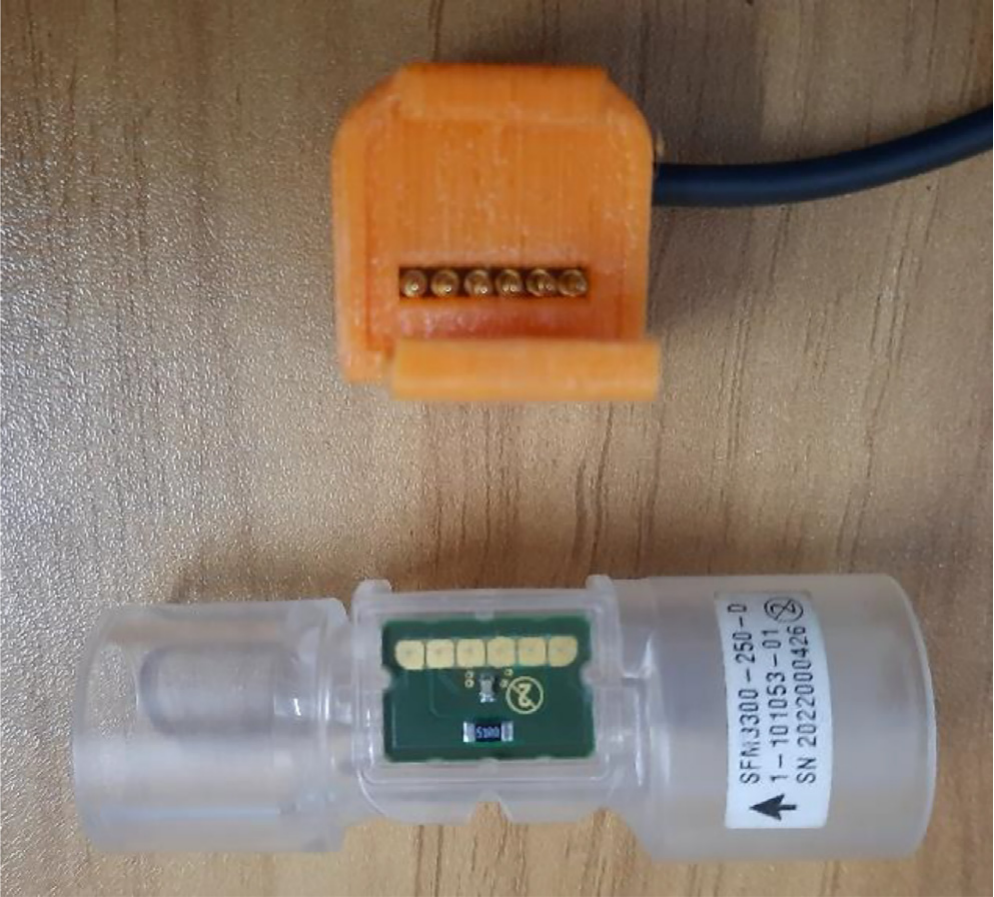
SFM 3300 flow sensor with custom 3D printed casing.

**Fig. 18:**
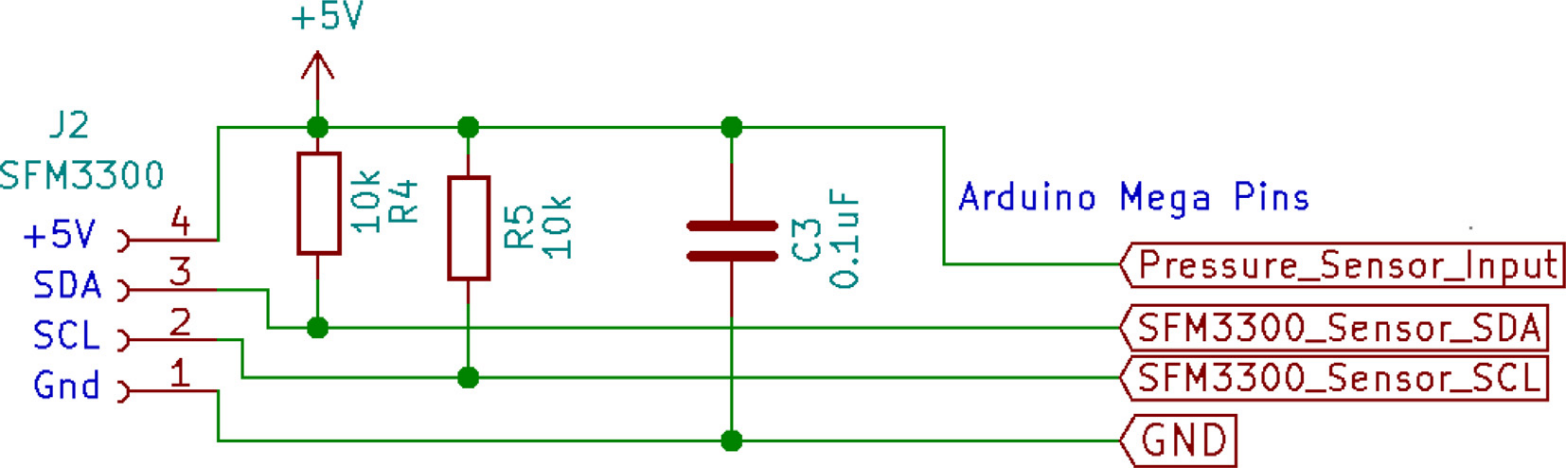
Circuit for SFM3300 with pull-up resistors.

**Fig. 19:**
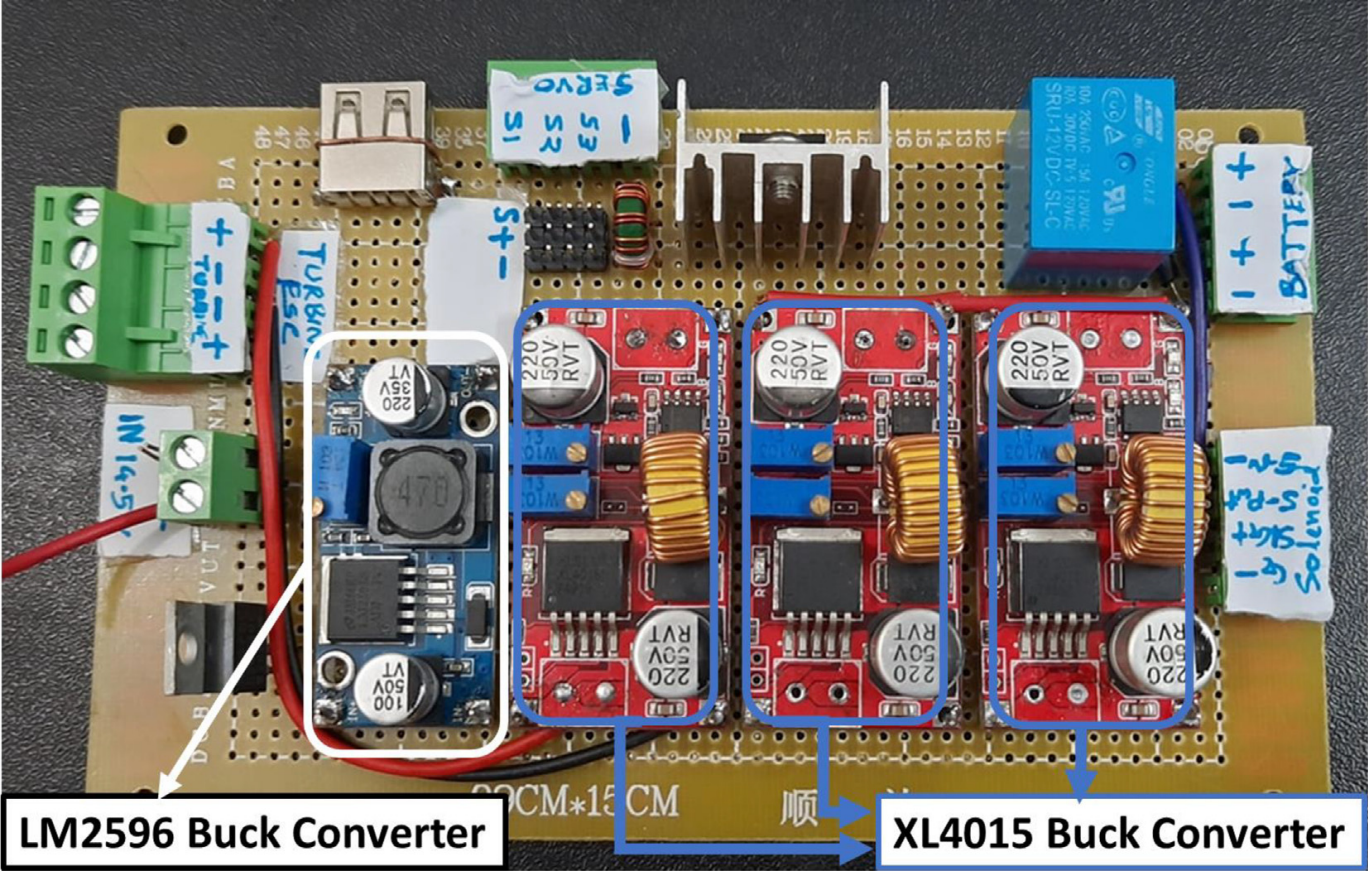
Power Distribution Board.

Upon not coming across any ready-to-be-used formula for converting an analog value to cmH2O, an experiment was set up to find out the constant; 0.20916, used in the Eq. (2). A *t*-connector [14] was used as a junction for 3 ports. One of those ports was connected with a syringe, one end with the pressure sensing outlet of MPX2010DP and finally, the third end was connected with a 4 mm pipe. The pipe was then put underwater and held by a measuring cylinder. A ruler was held in parallel to the measuring cylinder by its side with a clamp to measure the height difference that occurred when pressure was exerted by the syringe. When 1 cmH2O displacement of water was observed, the change in the analog value of the differential pressure sensor was recorded. This experiment was repeated 20 times and the mean value was finally taken to calculate the constant that has been used in the Eq.(2).

(2) $$pressure\left(cmH2O\right)=\;\left(Analog\;-\;AnalogOffset\right)*\;0.20916\;$$

(ii) SFM 3300:

SFM3300 sensor (Fig. 17) does not require any calibration but an external circuit Fig. 18] is needed for I2C communication. On top of that, SFM3300 [12] draws less than 50mW of power; draining only a 10 mA current at 5 V, enabling it to be powered by the microcontroller’s digital pin only.

c. Power modules:

A modular design that is based on electronic modules that can easily be picked up from the market is used here. A Vero-board design (Fig. 19) based on Fig. 11 is used so that it can also eliminate the dependency on manufacturing PCBs. But the design can surely be scaled to be manufactured using PCBs. Moreover, the use of electronic modules instead of individual SMD or THT components has enabled our system to be manufactured even in situations where expensive and complex pick and place machines are not available.

**Fig. 20:**
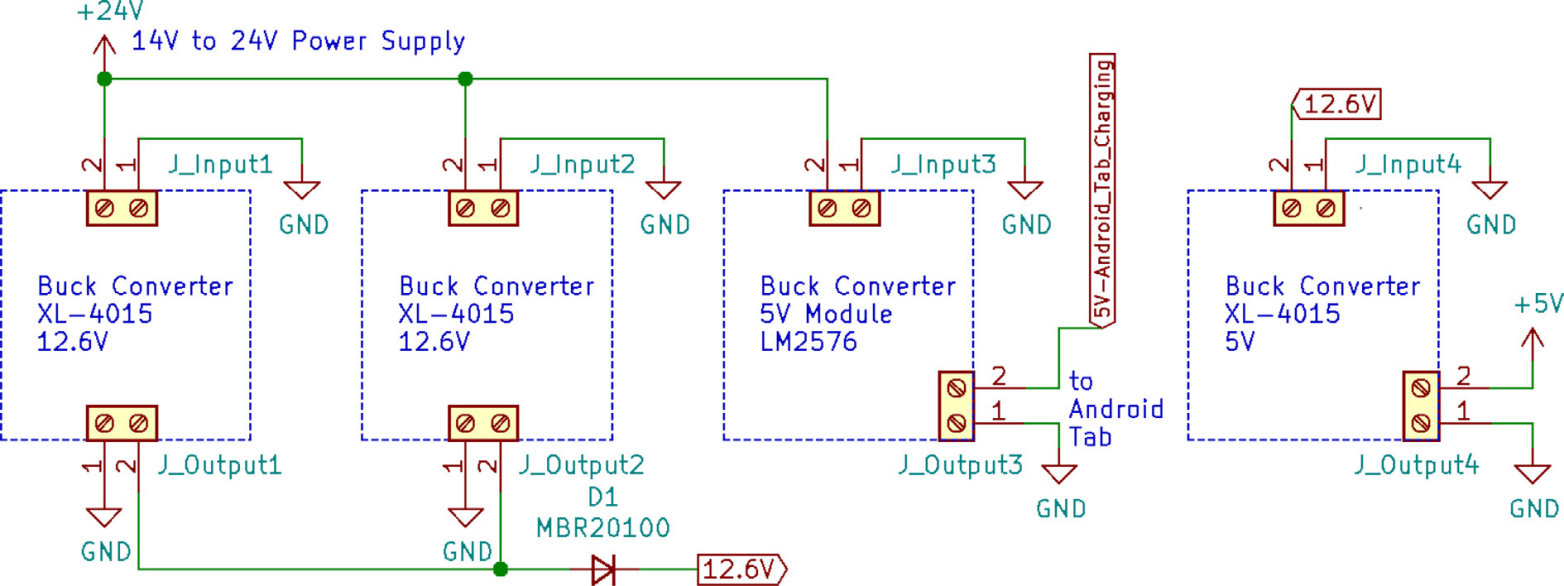
Schematic of the power distribution board.

The schematic of the connections of the module used in (Fig. 19) is demonstrated in the Fig. 20.

(i) Buck Converters:

Two types of modules; LM2596 and XL4015 buck converters are used in the power distribution board as can be seen in Fig. 17. Both of these converters are connected to a 24 V power supply.

**Fig. 21:**
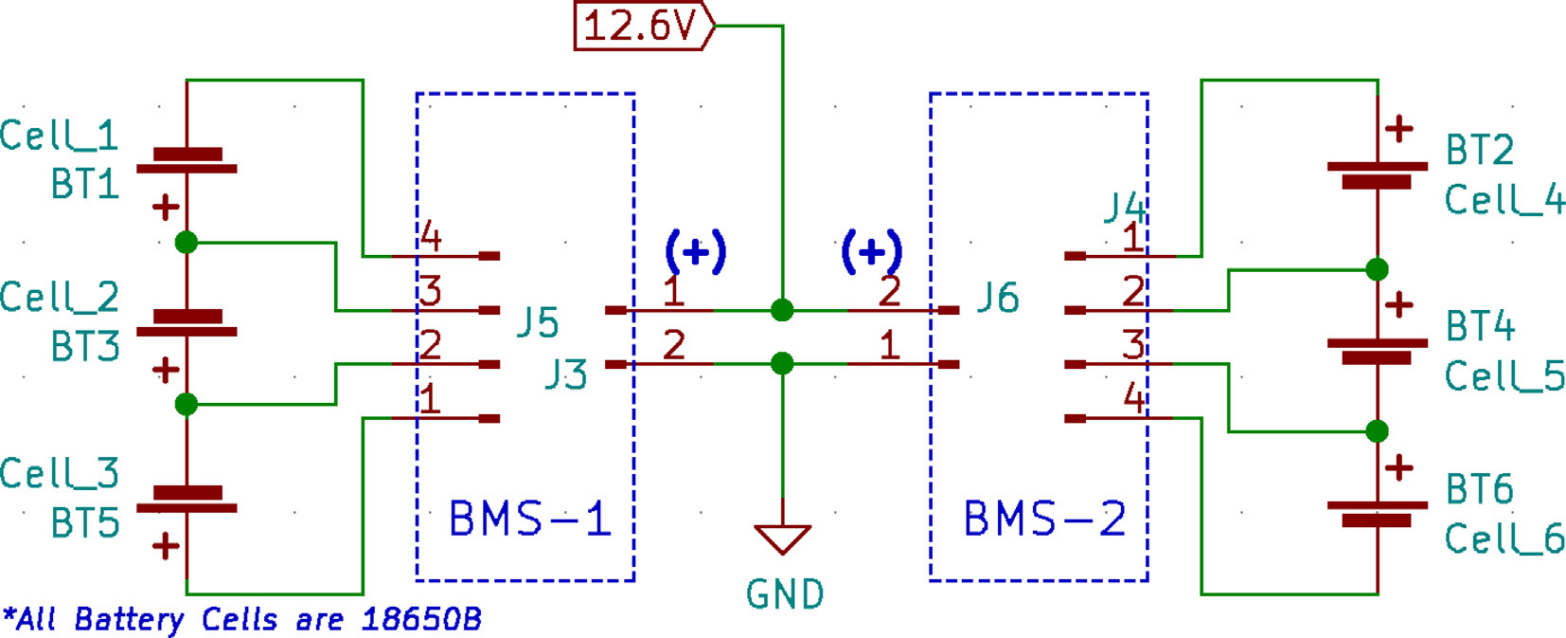
Schematic of the BMS.

• LM2596: It is a DC-DC step-down module. This converter’s output is regulated to 12.6 V to power the dual BMS and eventually charging the battery pack. Module specifications are given below:
  o Input voltage 3.2 V to 40 V
  o Output voltage 1.25 V to 35 V
  o Output current 3A (max)
  o Conversion efficiency 92 percent (the highest)
  o Output ripple less than 30 mV
  o Switching frequency 65KHz.
• XL4015: It is a 180KHz fixed frequency PWM buck (step-down) DC/DC converter, capable of driving a 5A load with high efficiency, low ripple, and excellent line and load regulation.
• Three identical power converters are used to provide power to three main modules of the system:
  (a) Android Tablet
  (b) Microcontroller
  (c) Other systems including (turbine, BMS, etc.)
• Module specifications are given below:
  o Input voltage range:8-36VDC
  o Output voltage range:1.25-36VDC adjustable
  o Output current: 0-5A
  o Output power: 75 W
  o High efficiency up to 96 %
(ii) BMS:

Two BMS modules have connected that work independently with two battery packs so that even if one of the BMS modules fails, there is still another as backup, hence increasing reliability further and decreasing the charging time. Fig. 21 shows the schematic of the dual BMS.

(iii) Battery packs:

Panasonic NCR18650B batteries [15] had been used as backup power instances of portable operation and during power outage/instability. A 3S2P configuration as shown in Fig. 21 used, ultimately getting 6800mAh, 12.6 V, and 85.68Wh. Our system is expected to run for 5.6 h at a stretch with the settings mentioned in Table 2. In Table 2, we have demonstrated a test that was conducted for 10 mins. Using formula of average power consumption by dividing the energy consumed by time, 15.27 W has been calculated for an hour with respect to the energy consumed in 10 mins.

**Table 2:** Ventilator system test summary.

Test Duration (mins)	10
BPM	10
Inspiration Time, Ti (s)	2
Pressure (cm H_2_O)	35
Energy Consumed (Wh)	2.544

However, using lower pressure settings will enable the device to run even longer, for example, it can run for a maximum of 8 h under a 20 cm H_2_O pressure setting.

### Modes of ventilation

Intensive care units (ICU) admitted patients are often given mechanical ventilation to reduce work of breathing (WOB), improve oxygenation, or correct respiratory acidosis without damaging the lungs while enabling the respiratory muscles to rest [14].

**Fig. 22:**
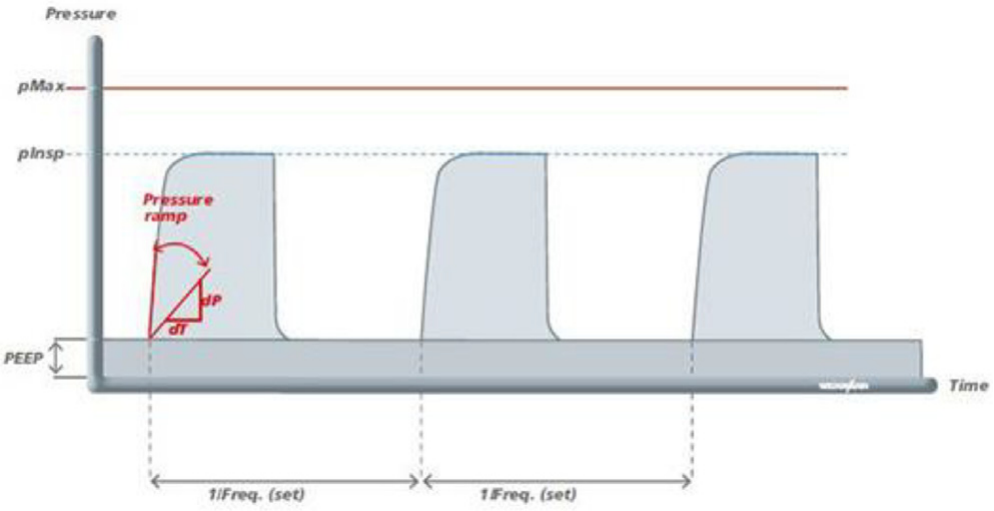
Pressure changes in PCV mode [17].

Considering the latest and most prominent modes, we have developed our system with the following modes:

(a) Pressure Regulated Volume Control (PRVC) mode-
  It is a combined volume and pressure regulated mode of ventilation that attempts to reach the targeted (user inputted) tidal volume (V_T_) with the lowest possible pressure.
  PRVC helps prevent volutrauma and barotrauma [16] by limiting the delivery pressure as it is an adaptive control form of ventilation that permits involuntary tuning of targets (pressure versus volume) through the compilation of data from the previous breathing cycles. Flow rates are adjusted to reach patients’ demands even when their lung compliance changes.
  PRVC adjusts flow rates to adapt with demand when lung compliance changes; subsequently achieving a normal breathing pattern. This mode is often the go-to mode for infants with ARDS. Besides that, PRVC provides the comfort of pressure ventilation for patients with reliable minute ventilation.
(b) PCV (Pressure Control Ventilation)-
  It’s pressure-controlled ventilation where the desired Peak Inspiration Pressure (PIP) is taken in from the doctor and the required tidal volume (V_T_) is delivered to achieve that pressure. Besides that, the PEEP is also maintained as the minimum pressure (Fig. 18). Mandatory breathes are provided to the patients but breath can be initiated by the patient if required; intermittent breathes [17]. However, considering patients’ safety, a maximum pressure (P_Max_) is hardcoded into the system.
(c) SIMV (Synchronous Intermittent Mandatory Ventilation)- In this mode, ventilators synchronize with the patient spontaneous breaths while maintaining the mandatory breathing. Synchronization is either flow or pressure triggered. In this ventilator, if patient breathing effort is detected within the synchronization time window and the flow or pressure is more or equal to the setpoint, then the ventilator provides the next breath earlier than the machine trigger breath to synchronize with patient effort (see Fig 22).

### Software

(a) Android Application –

The efficient development of the app; sizing only 5 MB, made it possible to run using low resources, subsequently decreasing the price of the device used to operate. Amongst all the options, Java was chosen as the language to be used to develop because it is reliable, has available libraries, has easy debugging, and adaptability to changes. The scale of the graphs was adjusted automatically by the system to provide better visibility.

Besides that, debugging is also expected to be easier due to the development being done on the Android platform on a Java codebase. Our app handled the serial communication seamlessly without any failure at a baud rate of 9600 where the predetermined data frame containing multi-variant values was lined up in single strings. Afterward, that string is segmented based on the preset data length of each variable and portrayed on the necessary graphs which could optimize its scale and provided better visibility to perceive useful information.

An implementation of the multithreading [18] was inevitable because of our application’s asynchronous [19] nature, which enabled us to maintain uninterrupted serial communication between the hardware and the software while performing background tasks. Firstly, software needs to establish communication with hardware and persist its connection for further data transmission. Secondly, there are many modes of the system like ‘PRVC’, ‘PCV’, and ‘SIMV’ that the software must be able to change in the runtime. Besides, the system must support initiating alarm in critical situations and snoozing it manually for example, when the value of ‘Tidal Volume’, ‘PIP’, or ‘PEEP’ is above or below the optimum limit.

To facilitate all the asynchronous activities, the ‘Thread Looper Handler Architecture’ [20] of android was put into use. When the software creates a socket [21] connection with the hardware, the software always receives and sends data in the form of a stream. Subsequently, a thread is assigned to manipulate the stream as the data in the steam is always in ASCII form. In, android it is not ideal to make the UI thread busy for performing heavy tasks as the UI might become unresponsive. So, we incorporate a handler [20] that can efficiently send data to the UI thread as well as receive it. The handler allows sending and processing of Message and Runnable objects associated with a thread’s MessageQueue. It will deliver messages and runnable to that message queue and execute them as they come out of the message queue.

The user interface was designed in a way that operators can easily observe the critical data on the display from a distance of 2 m at a glance and can conduct an operation with minimum clicks. Here, not only the alarms can only be seen clearly but also the interactive UI permits the users to change the conditions given for the alarms; both upper and lower bounds, providing more confidence to run the ventilator. Alarm settings can be turned on/off from the android app GUI while other settings like BPM, Ti and Tv can all be set using sliders. Nevertheless, debugging string can also be turned on for the developers, it shows the string being received through the Bluetooth module.

The application is built to be run on most of the tablets found on the market. However, this would be the minimum specification requirements needed to run the system smoothly is given below:

• Processor Type: Quad-Core
• Processor Speed: 1.5 GHz
• RAM: 2 GB DDR3
• Storage: 16 GB
• Display Size: 10 Inch
• Resolution: 1280*800 IPS
• OS Version: Android 9.0
• Bluetooth Functionality
• Dimension: 240 × 170 × 9 mm
(b) Firmware:

Firmware plays a vital role in proper integration between the software and hardware. The system must run uninterruptedly maintaining the desired motor speed of the turbine and performing other necessary actuations while taking useful inputs from the different sensors and GUI simultaneously. The user inputs are sent from the Android application to the microcontroller via a bluetooth module (HC-05) [11], which are then inputted into different control algorithms and other processes.

Control algorithms; Proportional Integral Derivatives (PID) and feed-forward, have been designed and embedded into the firmware for the system to become robust to changes and provide effective outputs while operating the modes discussed before.

**Fig. 23:**
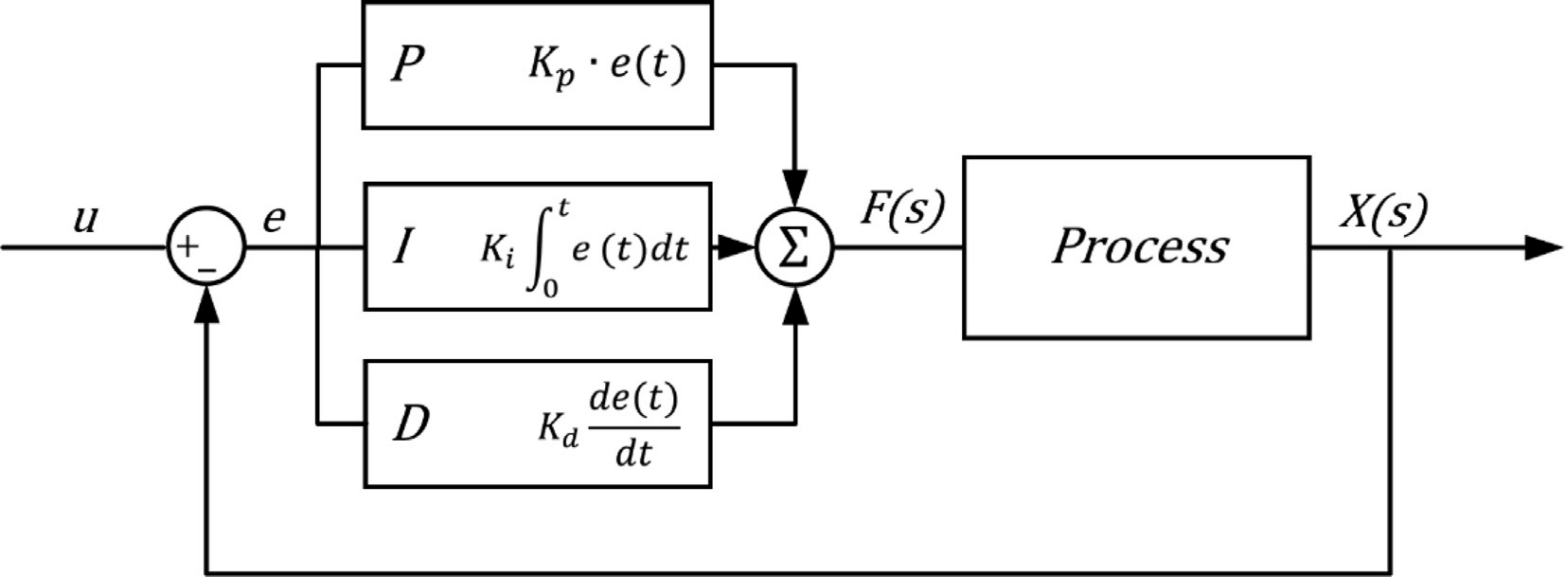
PID controller architecture.

**Fig. 24:**
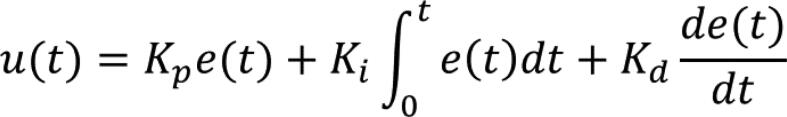
Control equation of PID [11].

PID controller is a closed-loop control system with a feedback control loop that evaluates the error based on the setpoint provided to the system (Fig. 23). To minimize the error, each part of the controller of P, I and D work hand in hand and then output is generated with each iteration while sending a feedback signal back to the system. Fig. 24 represents a control equation for PID, where the error [e(t)] is manipulated through the three constants; K_p_, K_i_ and K_d_. Not all of the PID is used; for instance, we have just used a ‘P’ controller in our ventilator.

Equation (3) was implemented to regulate the motor speed. The controller adjusts the value of the ‘volumeError’ in this formula. The value of Kp is determined after numerous trials and errors.

**Fig. 25:**
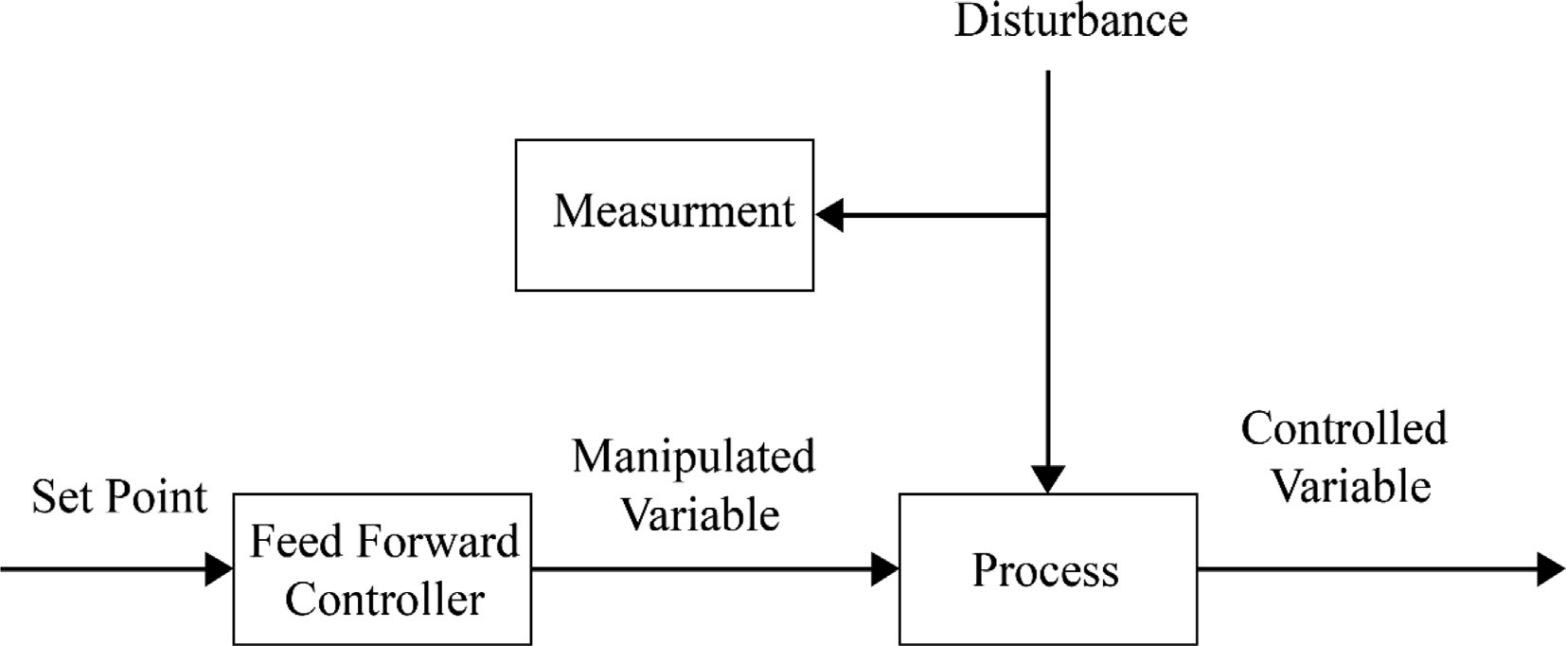
Feedforward Control Algorithm.

A set of pre-determined values for the motor speed is given into the feedforward algorithm (Fig. 25) for the range of inputs that can be taken from the operator. This ensures the system reaches the targeted value quickly. The value of ‘motorSpeed’ was the input given into the system using Eq.(3).

(3) motorSpeed=motorSpeed+volumeError∗Kp

Firmware is solely responsible for conducting all the calculations, switching between different modes while maintaining all the safety procedures, and finally triggering necessary alarms if necessary.

While processing calculations on embedded systems we need to keep in mind a lot of things, such as the memory occupied to run each of the functions, not to run out of the heap memory, etc. to run programs efficiently. Breathe cycle time is a very important factor for the system to proceed to the next steps is calculated by Eq.(4) and it is a summation of both inspiration and expiration times (as in Eq.5).

(4) $$\mathrm{BreatheCycleTime},BCT\left(s\right)=\frac{60}{\mathrm{BPM}}$$

(5) $$BCT\left(s\right)=Insptime\left(s\right)+Expitime\left(s\right)$$

A predetermined data string frame is used for communication between the firmware and the Android tablet, for example, “v061502.0042090050025150702″. Such a string is then broken-down using substring [22] to retrieve important data and put it into respective variables for the system to process later on.

(i) Sensor data filtration methodology.

Data is being retrieved continuously from the system but state-of-the-art hardware is, there will always be some noise that calls for some filtration before being used in any algorithm. The filtration algorithm is designed in the following steps:

a. Data array- Sensor data (x) is accumulated into an array sequentially according to its array size.
b. Mean ($\overset{¯}{x}$) is then calculated from the summation of the array values and data size using Eq.(6).

(6) $$\overset{¯}{x}=\frac{\sum x}{n}$$

c. Standard deviation ‘stdDev’ in Eq.(7) helps us to find out the extent of spreading of the sensor values and it is calculated using this formula Eq.(6).

(7) $$\mathrm{stdDev}=\sqrt{\frac{\sum {\left(x_{i}-\overset{¯}{x}\right)}^{2}}{n}}$$

d. The standard deviation is then used to draw a virtual boundary/ envelope around the mean data; giving a better sense of the data to be filtered. We will be calling this envelope ‘stdShrink’, which is calculated using Eq.(8). The shrinkPercent can be tweaked to get the desired stdShrink.

(8) $$\mathrm{stdShrink}=stdDev-\left(stdDev*shrinkPercent\right)$$

Fig. 25 shows better representation of the data that needs to be filtered. Existing sensor data are then checked if they are within the ‘DataBand’; $\overset{¯}{x}\pm stdShrink$. Data falling within this band would be summed to a new variable called filtered.

e. Finally, another mean is again calculated from the filtered data and then this means shall be used by other modules of the system. These steps enable us to not only filter unwanted data spikes and noises created by the sensor, hence, assuring value with higher accuracy.
  (ii) Modes of Ventilation:

To provide a seamless change in modes, the android app is used as a primary GUI where the mode is selected by the doctors.

a. PRVC-

In this mode, firstly the targeted volume, breaths per minute (BPM), inspiration time (T_I_), PEEP value, and alarm settings are taken into the system via the data string.

Upon entering the inhale state, the motor starts with a speed; feed-forward value from the feed-forward algorithm, that had been hardcoded into the embedded system. The motor speed is then adjusted every cycle by running the PID algorithm with a feed-forward algorithm in addition. Afterward, PIP and other alarm parameters are checked and respective alarms are triggered if necessary.

During the exhale state, the motor starts with a feed-forwarded motor speed to maintain the specific PEEP value PID is again put into work. Alarm conditions are checked again and triggered accordingly.

Finally, if the conditions for the synchronized intermittent mandatory ventilation (SIMV) mode are met, the spontaneous breath will be provided.

b. PCV-

During this mode, the motor again starts with an initial speed that has been feed-forwarded beforehand, and here this value dynamically changes in every loop. Within inhale state, PID is used to maintain the motor speed Eq.(3) to keep the desired pressure by comparing raw pressure sensor data. The PIP is determined using the ‘Sensor Data Filtration Methodology’ [2.4.b.i]. The algorithm is then recalculated following the newly found value from the filtered sensor dataset. The new base value (feed-forward) for the motor is calibrated and the motor speed is maintained accordingly using PID. Other alarm parameters and patient circuit disconnection are checked and are triggered if necessary.

During exhale state, the motor starts with a feed-forwarded motor speed to maintain the specific PEEP value set by the healthcare professional. The motor speed that is needed is maintained using a PID algorithm. Subsequently, alarm conditions are checked again and triggered accordingly.

If the conditions for the SIMV mode are met, the breath will be triggered.

c. SIMV-

With the inputs of T_i_ and respiratory rate from the doctor, the breath cycle is calculated using Eq.(4). Then the ‘n’ is calculated from the breath cycle using Eq.(9). We have considered the sync time to be after 90 % of the breath cycle time but this can easily be changed in the code that we have provided. To calculate the time window for the SIMV, ‘syncTime’ is calculated using Eq.(10).

(9) nonSyncTime=breathCycleTime∗0.9

(10) SyncTime=nonSyncTime-Ti

**Fig. 26:**
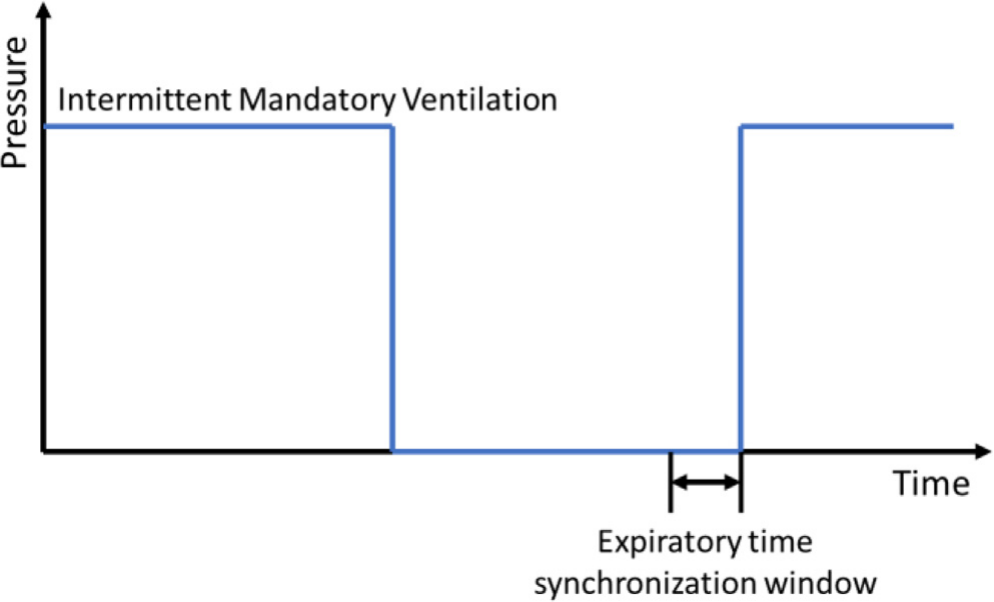
SIMV synchronization window [23].

If a patient tries to breathe within this ‘syncTime’ (Fig. 26), the flow rate is checked and if it is greater than 20 ml, breath is given. Value for the flow rate for the trigger is taken from the doctor. The sensitivity of the trigger can be tuned by adjusting the value of the flow rate. The same type of mechanism can be implemented using a Pressure sensor. However, it was implemented only at the expiratory time synchronization window.

d. Patient Circuit Disconnection Algorithm:

During the inhale state if PIP is found to be lesser than 5 cmH2O, a disconnection is triggered. Here, the flow increases as the pressure cannot be built in an open patient circuit. Both audio and visual alarms will be triggered.

As a safety feature, the motor speed is decreased to the minimum level, however, when the patient circuit is reconnected, it can be detected.

Finally, a system was developed with easy operation, low start-up time, and fast calibration of the pressure sensor. However, an air relay can be used for calibrating the pressure sensor after a certain interval to improve the performance further.

## Design files

**Table:** 

**Assembly Unit**	**Design file name**	**File type**	**Open-source license**
Casing Base	base 11	CAD files (STEP)	CC BY-SA 4.0
	base 12	CAD files (STEP)	CC BY-SA 4.0
	base 21	CAD files (STEP)	CC BY-SA 4.0
	base 22	CAD files (STEP)	CC BY-SA 4.0
	PSU Holder Tab v1	CAD files (STEP)	CC BY-SA 4.0
	Front Extension 2 v1	CAD files (STEP)	CC BY-SA 4.0
	Pressure Hose Attachment v1	CAD files (STEP)	CC BY-SA 4.0
	Air Filter Vent v1	CAD files (STEP)	CC BY-SA 4.0
	PSU mount v1	CAD files (STEP)	CC BY-SA 4.0
	Holder	CAD files (STEP)	CC BY-SA 4.0
Casing Top	top 11	CAD files (STEP)	CC BY-SA 4.0
	top 12	CAD files (STEP)	CC BY-SA 4.0
	top 21	CAD files (STEP)	CC BY-SA 4.0
	top 22	CAD files (STEP)	CC BY-SA 4.0
	Side Vent v1	CAD files (STEP)	CC BY-SA 4.0
	LCD Holder 1	CAD files (STEP)	CC BY-SA 4.0
	LCD Holder 2	CAD files (STEP)	CC BY-SA 4.0
	LCD Holder 3	CAD files (STEP)	CC BY-SA 4.0
	debug cover	CAD files (STEP)	CC BY-SA 4.0
	charging port	CAD files (STEP)	CC BY-SA 4.0
Tab Holder	front 11	CAD files (STEP)	CC BY-SA 4.0
	front 12	CAD files (STEP)	CC BY-SA 4.0
	back 11	CAD files (STEP)	CC BY-SA 4.0
	back 12	CAD files (STEP)	CC BY-SA 4.0
	Tab Cover 1	CAD files (STEP)	CC BY-SA 4.0
	Tab Cover 2	CAD files (STEP)	CC BY-SA 4.0
	Button1 v1	CAD files (STEP)	CC BY-SA 4.0
	Button2 v1	CAD files (STEP)	CC BY-SA 4.0
	3 mm Pin	CAD files (STEP)	CC BY-SA 4.0
Peep Valve Holder Extension	Front Extension 1 (Base)	CAD files (STEP)	CC BY-SA 4.0
	Front Extension 1 (Attachment	CAD files (STEP)	CC BY-SA 4.0
Tab Support	Holder1	CAD files (STEP)	CC BY-SA 4.0
	Holder2	CAD files (STEP)	CC BY-SA 4.0
	Holder3	CAD files (STEP)	CC BY-SA 4.0
	Support	CAD files (STEP)	CC BY-SA 4.0
Pressure Release Mechanism	EOM(Base)	CAD files (STEP)	CC BY-SA 4.0
	EOM (Servo Bracket)	CAD files (STEP)	CC BY-SA 4.0
	EOM (Push Body)	CAD files (STEP)	CC BY-SA 4.0
	Exhaust Valve Gate v1	CAD files (STEP)	CC BY-SA 4.0
	Outlet Body	CAD files (STEP)	CC BY-SA 4.0
	Adapter_14mm (Long) v1	CAD files (STEP)	CC BY-SA 4.0
	Adapter14mm v1	CAD files (STEP)	CC BY-SA 4.0
	Outlet Base	CAD files (STEP)	CC BY-SA 4.0
	Stopper Bolt	CAD files (STEP)	CC BY-SA 4.0
	Stopper Gasket	CAD files (STEP)	CC BY-SA 4.0
	Silicon Seal	CAD files (STEP)	CC BY-SA 4.0
	Screw	CAD files (STEP)	CC BY-SA 4.0
	EGV Base	CAD files (STEP)	CC BY-SA 4.0
	MG995 v1	CAD files (STEP)	CC BY-SA 4.0
	Servo Horn v1	CAD files (STEP)	CC BY-SA 4.0
	Exhaust Gate Valve Coil Spring v1	CAD files (STEP)	CC BY-SA 4.0
One Way Valve	One Way Valve Inlet	CAD files (STEP)	CC BY-SA 4.0
	One Way Valve Outlet	CAD files (STEP)	CC BY-SA 4.0
HME Filter	HME Body	CAD files (STEP)	CC BY-SA 4.0
	HME Cover	CAD files (STEP)	CC BY-SA 4.0
	HME Inlet Mesh	CAD files (STEP)	CC BY-SA 4.0
Turbine and Proportional Valve Mechanism	WM7040 Pump v1	CAD files (STEP)	CC BY-SA 4.0
	Inlet Body v1	CAD files (STEP)	CC BY-SA 4.0
	Proportional Valve Body	CAD files (STEP)	CC BY-SA 4.0
	Proportional Valve Cover	CAD files (STEP)	CC BY-SA 4.0
	Adapter_14mm (Short) v1	CAD files (STEP)	CC BY-SA 4.0
	Adapter_14mm (Pump Outlet) v1	CAD files (STEP)	CC BY-SA 4.0
	Micro Tower 9 g v1	CAD files (STEP)	CC BY-SA 4.0
	Hose_OXY	CAD files (STEP)	CC BY-SA 4.0
	Hose_Release	CAD files (STEP)	CC BY-SA 4.0
Peep Valve Assembly	peep inlet	CAD files (STEP)	CC BY-SA 4.0
	peep outlet	CAD files (STEP)	CC BY-SA 4.0
	Peep Extension	CAD files (STEP)	CC BY-SA 4.0
	Peep Valve Attachment	CAD files (STEP)	CC BY-SA 4.0
	Peep Hose	CAD files (STEP)	CC BY-SA 4.0
Oxygen Inlet Nozzle	OXY Nozzle Body	CAD files (STEP)	CC BY-SA 4.0
	OXY Nozzle Holder	CAD files (STEP)	CC BY-SA 4.0
Battery Pack	3S Battery Holder v1	CAD files (STEP)	CC BY-SA 4.0
	PANASONIC_NCR-18650B v1	CAD files (STEP)	CC BY-SA 4.0
Power Supply	SMPS v1	CAD files (STEP)	CC BY-SA 4.0
Android Tab	Tab v1	CAD files (STEP)	CC BY-SA 4.0
Switch, Connector and Module	Rotary Encoder Module v1	CAD files (STEP)	CC BY-SA 4.0
	Knob 20 mm v1	CAD files (STEP)	CC BY-SA 4.0
	Switch DPDT v1	CAD files (STEP)	CC BY-SA 4.0
	Power Socket v1	CAD files (STEP)	CC BY-SA 4.0
	Push Button v1	CAD files (STEP)	CC BY-SA 4.0
	Indicator v1	CAD files (STEP)	CC BY-SA 4.0
	DC Power Socket v1	CAD files (STEP)	CC BY-SA 4.0
	DMX 5pin v1	CAD files (STEP)	CC BY-SA 4.0

## Bill of materials

**Table:** 

**Designator**	**Component**	**Number**	**Cost per unit (USD)**	**Total cost (USD)**	**Source of materials**
WM7040 Pump v1	Turbine	1	53	53	https://www.aliexpress.com/item/10000048326249.html?
	SFM3300-250-D Flow Sensor	1	45	45	https://www.digikey.com/en/products/detail/sensirion-ag/SFM3300-250-D/9857673
	Pressure Sensor- MPX2010DP	1	19	19	https://www.digikey.com/en/products/detail/nxp-usa-Inc/MPX2010DP/410890?
	Arduino Mega	1	9.49	9.49	https://www.aliexpress.com/item/32850843888.html?
	Buck Converter - XL4015	3	1.3	3.9	https://www.aliexpress.com/item/32651786894.html?
	Buck Converter - LM2596	1	1	1	https://www.aliexpress.com/item/4000064597454.html?
	Digital Encoder	1	1	1	https://www.aliexpress.com/item/10000000931574.html?
MG995 v1	MG995 Servo	1		2	https://www.aliexpress.com/item/4001223064938.html?
Micro Tower 9 g v1	Sg90 Micro Servo	1	1	1	https://www.aliexpress.com/item/1005002056653701.html?
	Op-Amp LM358	1	0.53	0.53	https://www.digikey.com/en/products/detail/texas-instruments/LM358PE4/1510269
	Diode MBR20100	1	1.07	1.07	https://www.digikey.com/en/products/detail/smc-diode-solutions/MBR20100/6022125
	4.7 K Ohms Resistor	2	0.26	0.52	https://www.digikey.com/en/products/detail/vishay-beyschlag-draloric-bc-components/MBB02070C4701FCT00/7351222
	10 K Ohm Metal Film Resistor	2	0.4	0.8	https://www.digikey.com/en/products/detail/vishay-beyschlag-draloric-bc-components/PR02000201002JA100/7351718
	1 M Ohm Resistor	1	0.26	0.26	https://www.digikey.com/en/products/detail/vishay-beyschlag-draloric-bc-components/MRS25000C1004FRP00/5063981
	Capacitor 104/100nF	2	0.45	0.9	https://www.digikey.com/en/products/detail/vishay-beyschlag-draloric-bc-components/K104K15X7RF53H5G/13279844
Tab v1	Android Tab 10.11″ Touch Screen	1	95	95	https://www.aliexpress.com/item/1005002310393179.html?
PANASONIC_NCR-18650B v1	18,650 Battery	6	3.65	21.9	https://www.aliexpress.com/item/4001217463600.html?
	BMS 3S1P	2	1	2	https://www.aliexpress.com/item/4000654183624.html?
3S Battery Holder v1	Battery Spacer	4	0.24	0.96	https://www.aliexpress.com/item/4001177937131.html?
	USB Cable 1 m	1	3	3	https://www.aliexpress.com/item/32950736308.html?
DMX 5pin v1	GX12-5 Connector	1	6	6	https://www.digikey.com/en/products/detail/mill-max-manufacturing-corp/816-22-006-10-001101/6149708
	Pogo Pins	1	4.76	4.76	https://www.aliexpress.com/item/1005002565524082.html?
	Solderless docking type 4P, 5.08MM screw terminal	3	0.5	1.5	https://www.aliexpress.com/item/1005002892133469.html?
DC Power Socket v1	DC Jack Female	1	0.2	0.2	https://www.aliexpress.com/item/32265708803.html?
	USB Type-A Standard Port Female	1	0.1	0.1	https://www.aliexpress.com/item/4000290521933.html?
	KF301-5.0-2P Green Connector	1	0.1	0.1	https://www.aliexpress.com/item/4001201565770.html?
	Straight Male Pin Header	1	0.1	0.1	https://www.aliexpress.com/item/32908642552.html?
	Male Header L Shape	1	0.8	0.8	
	L Connector				https://www.aliexpress.com/item/4000148427087.html?
Power Socket v1	Power Socket	1	0.3	0.3	https://www.aliexpress.com/item/1005002503501069.html?
	TO220 Heat Sink	1	0.5	0.5	https://bmabazar.com/shop/ventilator-patient-circuit/
	Heat Shrink	1	0.32	0.32	https://bmabazar.com/shop/viral-filter/
	Ventilator Patient Circuit	1	6	6	https://www.sunlu.com/products/new-pla-plus-pla-1-75-filament-1kg-2-2lbs?variant=31987368788054
	Viral Filter	1	5	5	https://www.aliexpress.com/item/33033655207.html?
	PLA Filament for 3D Printing	3	23	69	https://www.aliexpress.com/item/1005002511958246.html
Screw	Screw Assortment Kit	1	20	20	https://www.aliexpress.com/item/4000411749473.html?
	Epoxy Glue	5	0.91	4.55	https://www.aliexpress.com/item/1005001958579088.html?
Exhaust Gate Valve Coil Spring v1	Coil Spring:	1	2.29	2.29	https://www.aliexpress.com/item/32427862310.html?
	(Length 25 mm, Outer diameter 5 mm, Wire thickness 0.3 mm)				https://www.amazon.com/Medical-Platinum-Silicone-Tubing-Feet/dp/B0861LKZPC
	10 pcs Pack				https://www.ebay.com/itm/202643688057?
Stopper Gasket	Rubber O-ring kit	1	4.35	4.35	https://www.aliexpress.com/item/4000415201699.html?
	3 mm Linear Rod (2 Pcs kit, 3 mm*100 mm)	1	1.2	1.2	https://www.aliexpress.com/item/4000828822242.html?
Hose_OXY, Hose_Release	Silicon Tube 1 foot	2	1.2	2.4	https://www.aliexpress.com/item/4000652348770.html?
Silicon Seal	Silicone Sheet	1	8.95	8.95	https://www.aliexpress.com/item/10000048326249.html?
Peep Hose	4 mm Medical grade pipe	1	4.99	4.99	https://www.digikey.com/en/products/detail/sensirion-ag/SFM3300-250-D/9857673
	Heat Shrink Tube	1	2.2	2.2	https://www.digikey.com/en/products/detail/nxp-usa-Inc/MPX2010DP/410890?
Peep Valve Attachment, Front Extension 1 (Attachment)	Extension Tube (4 mm)	1	8.86	8.86	https://www.aliexpress.com/item/32850843888.html?

## Build Instructions

### Casing

1. Glue the edge of the base parts (base 11, 12 21 and 22) (see Fig. 27)

**Fig. 27:**
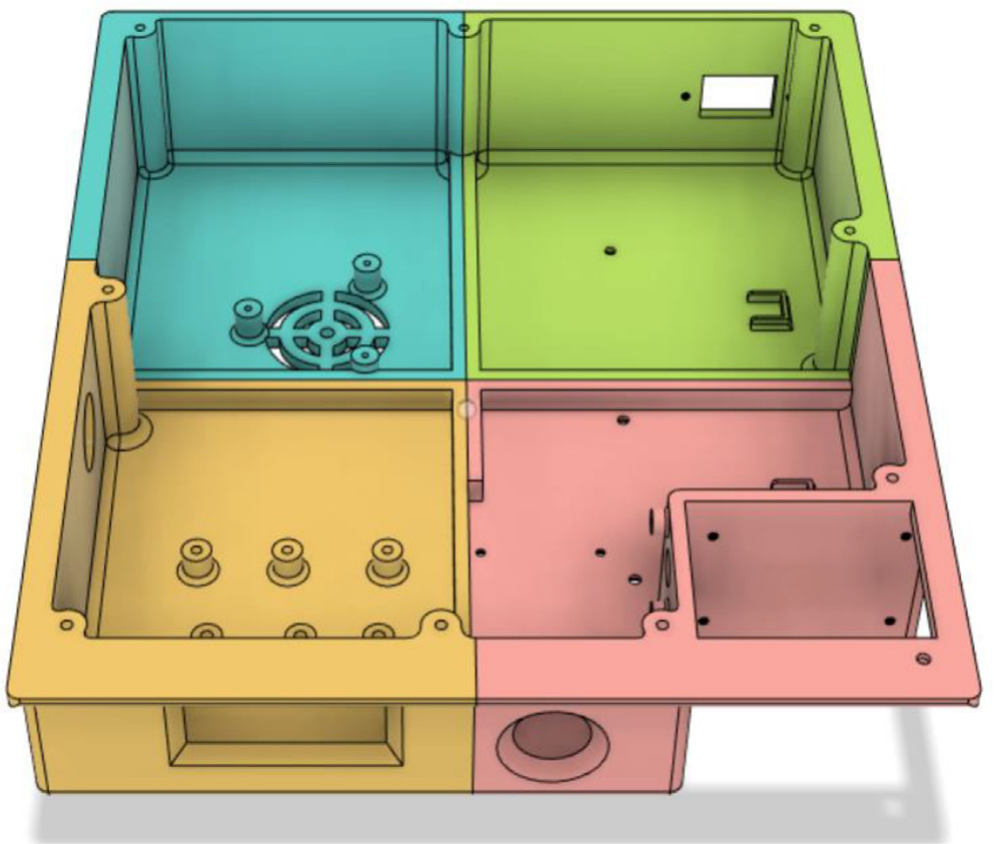
Base Parts.
2. Glue the bottom face of the ‘PSU Holder Tabs’ with the Casing Base (Base 11 and 22) (see Fig. 28)

**Fig. 28:**
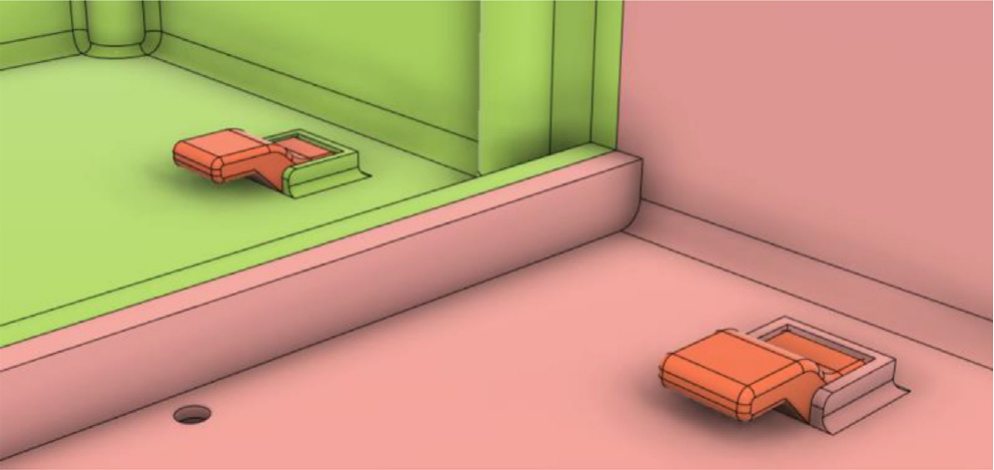
PSU Holder Tabs.
3. Glue the Front Extension 1 (Base) and Front Extension 1 (Attachment) together (see Fig. 29, Fig. 30)

**Fig. 29:**
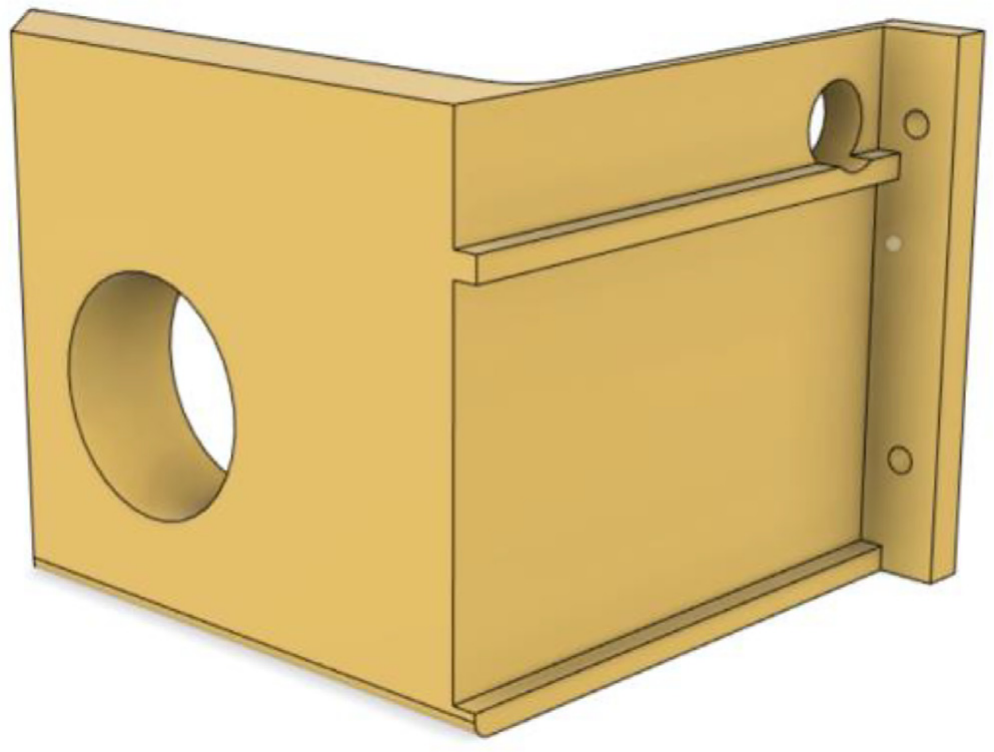
Front Extension 1 (Base).

**Fig. 30:**
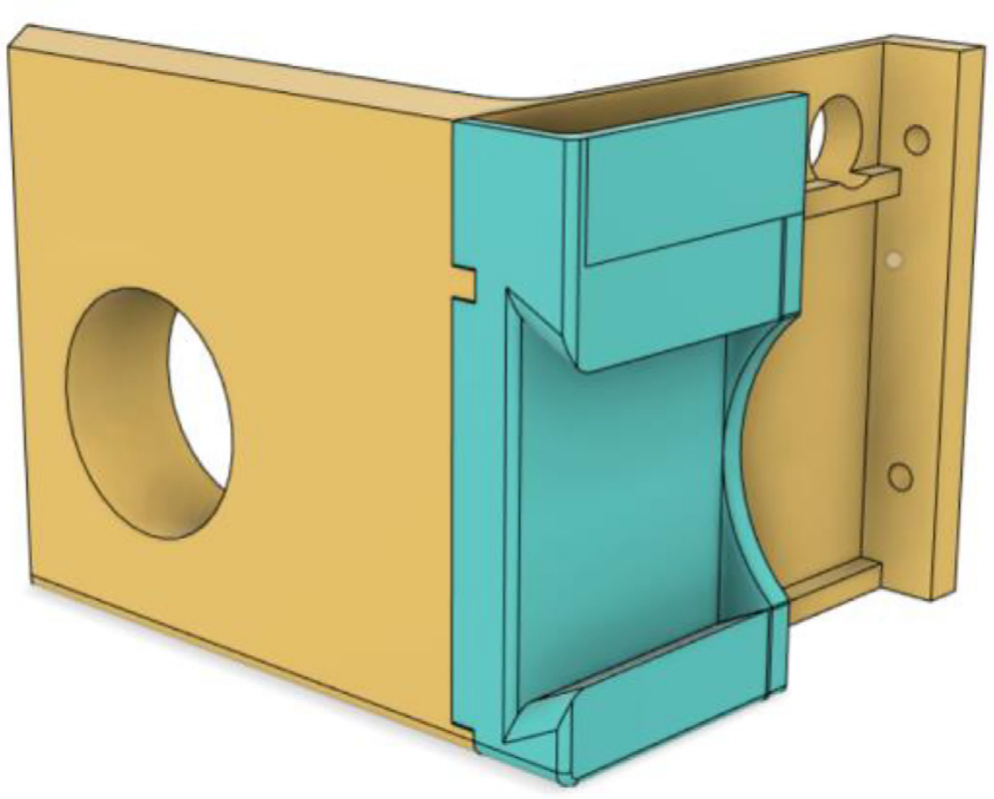
Front Extension 1 (Attachment).
4. Glue the inner parts of the Front Extension 1 as a whole to Base 11 (see Fig. 31)

**Fig. 31:**
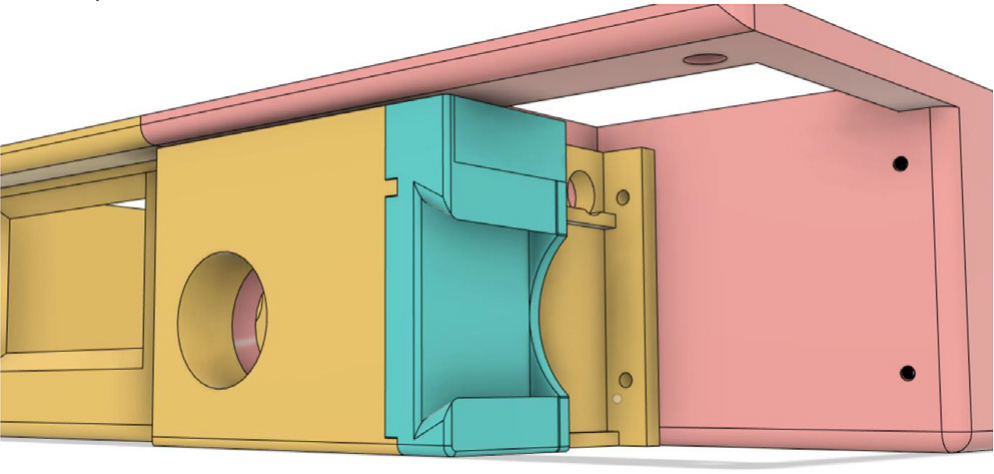
Front Extension 1 attached with the Base 11.
5. Attach the ‘Pressure Hose Attachment’ with the ‘Front Extension 2′ and then glue the edges of the ‘Pressure Hose Attachment’ to attach with the entire assembly with the ‘Base 11′ (see Fig. 32, Fig. 33)

**Fig. 32:**
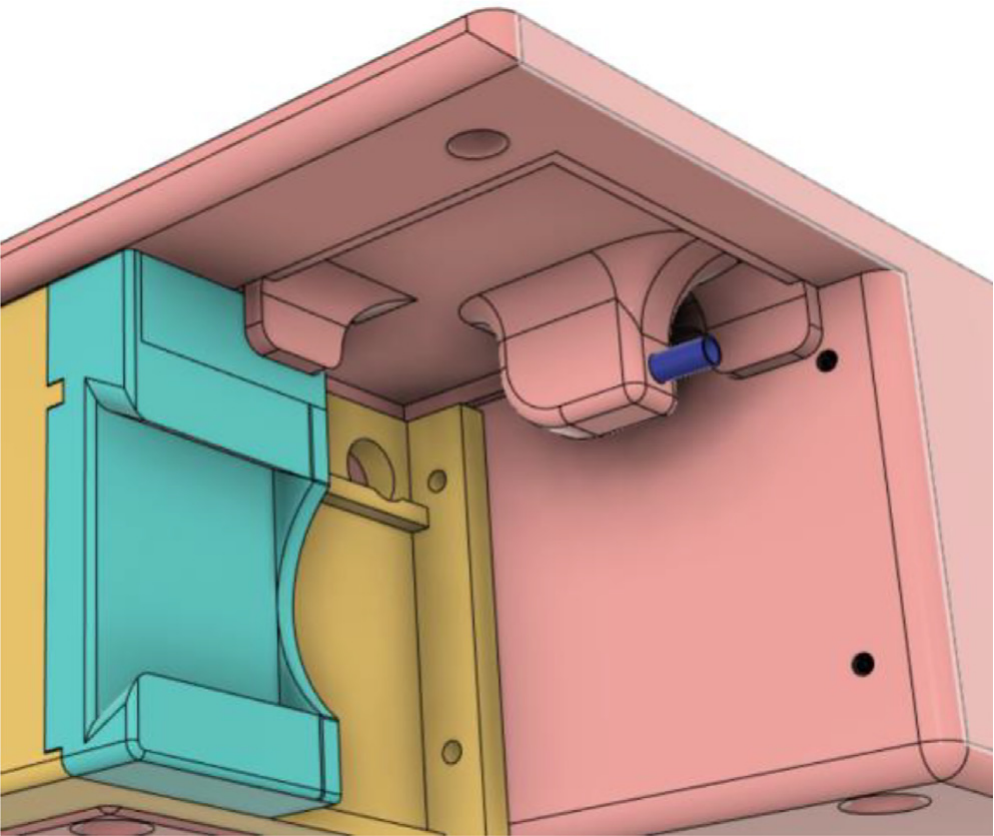
Bottom view of Front Extension 2.

**Fig. 33:**
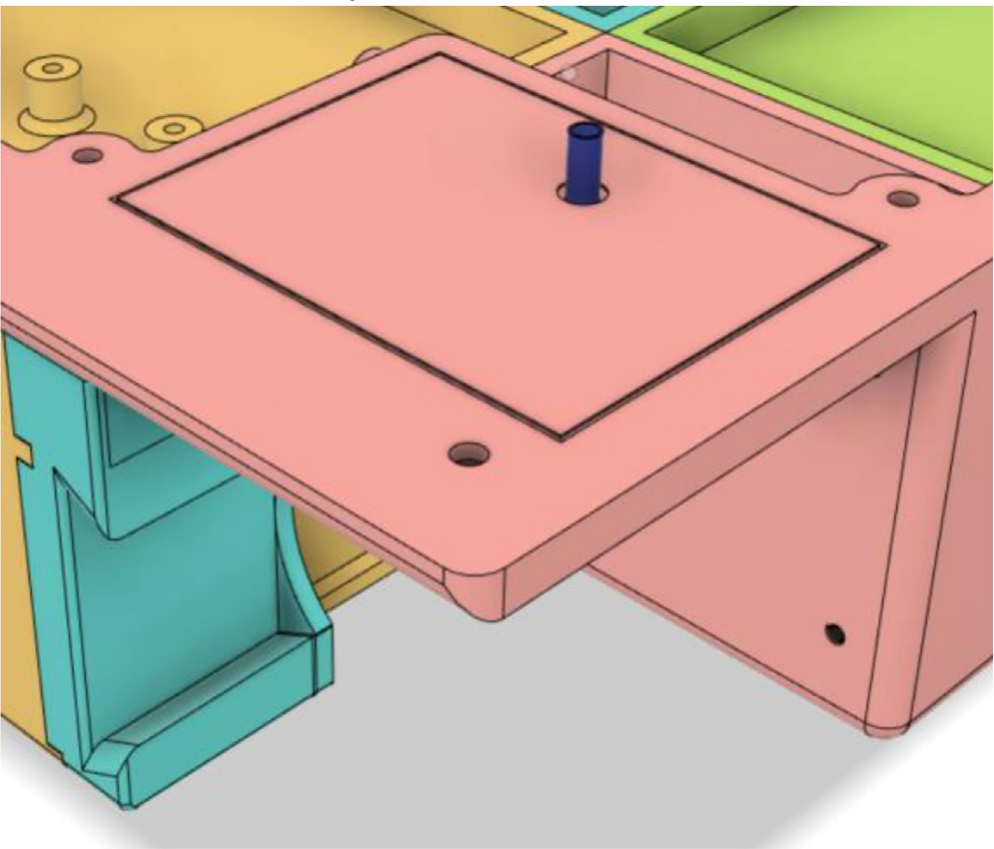
Top view of Front Extension 2.
6. Glue the edges of the ‘Air Filter Vent’ with the ‘Base 12′ (see Fig. 34, Fig. 35)

**Fig. 34:**
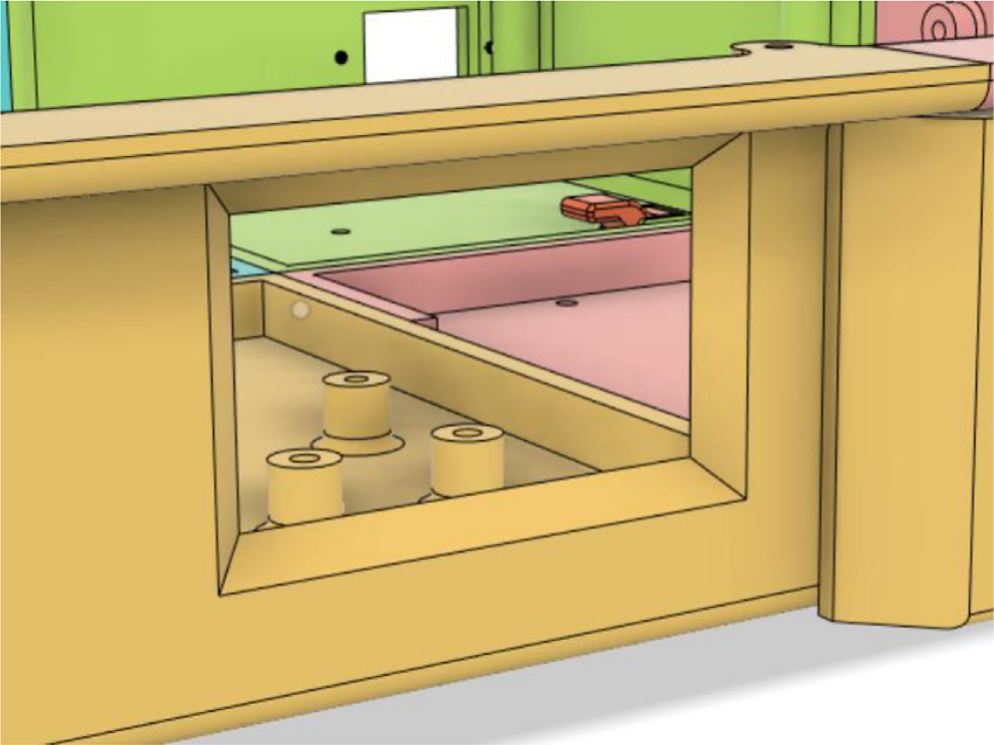
Without vent.

**Fig. 35:**
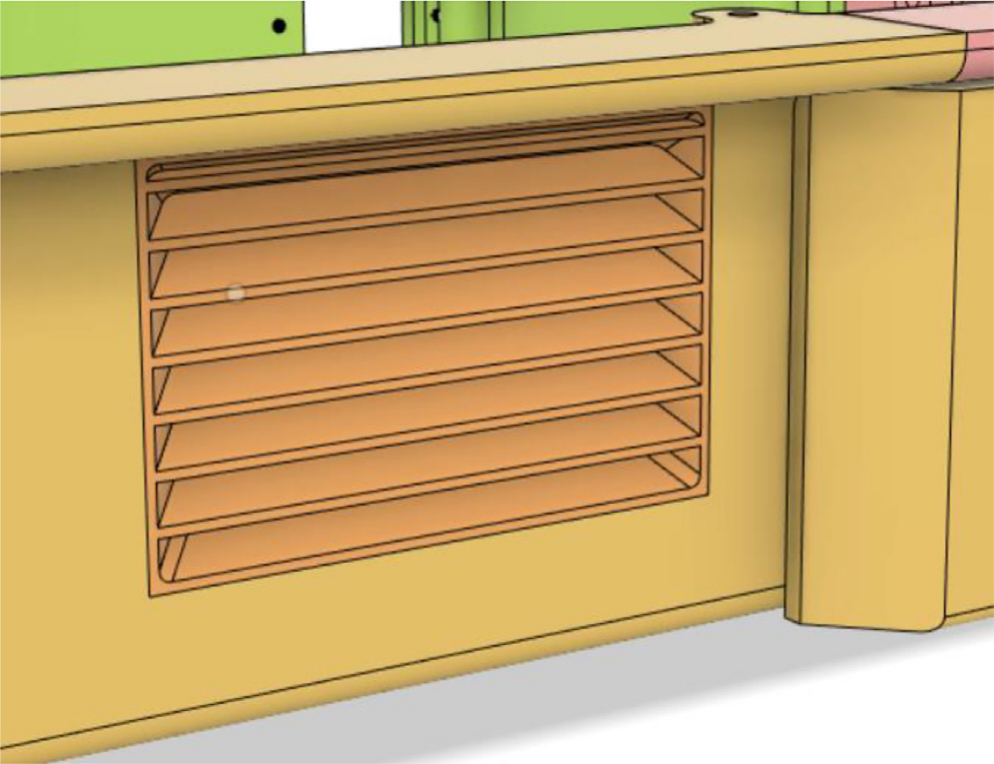
With vent.
7. Glue the edges of the Top Parts (Top 11, 12, 21 and 22) (see Fig. 36)

**Fig. 36:**
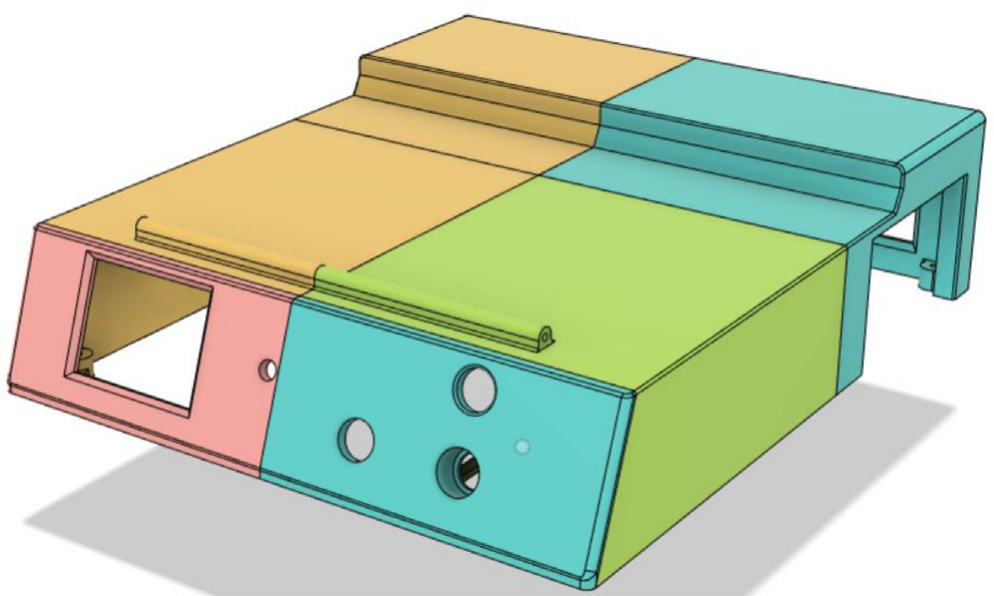
Top Parts glued together.
8. Glue the edges of the Side Vents to the Top Parts (Top 21 and 22) (see Fig. 37, Fig. 38)

**Fig. 37:**
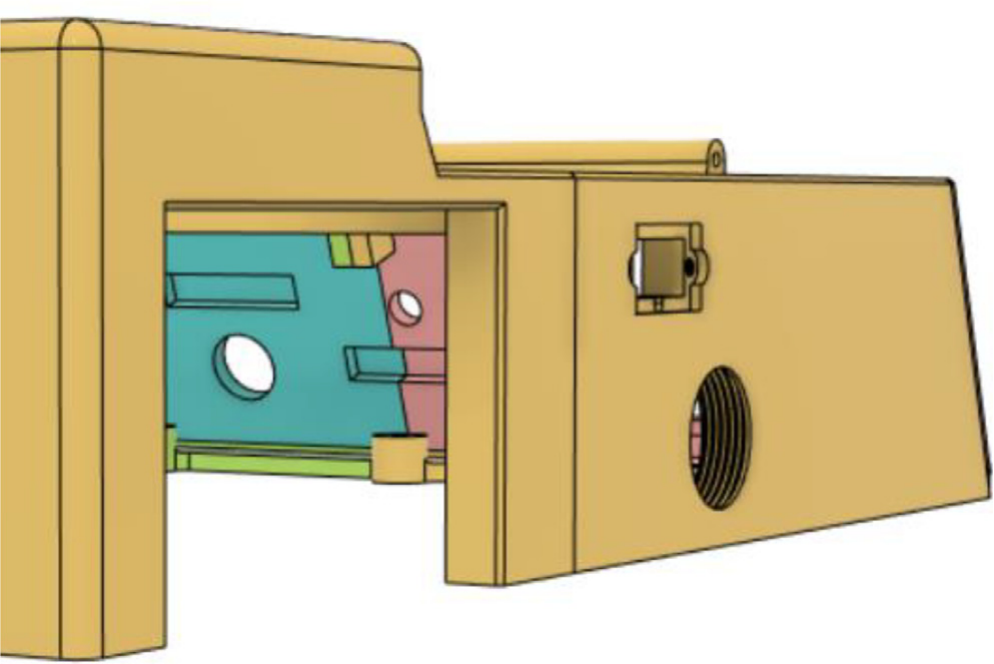
Without a side vent.

**Fig. 38:**
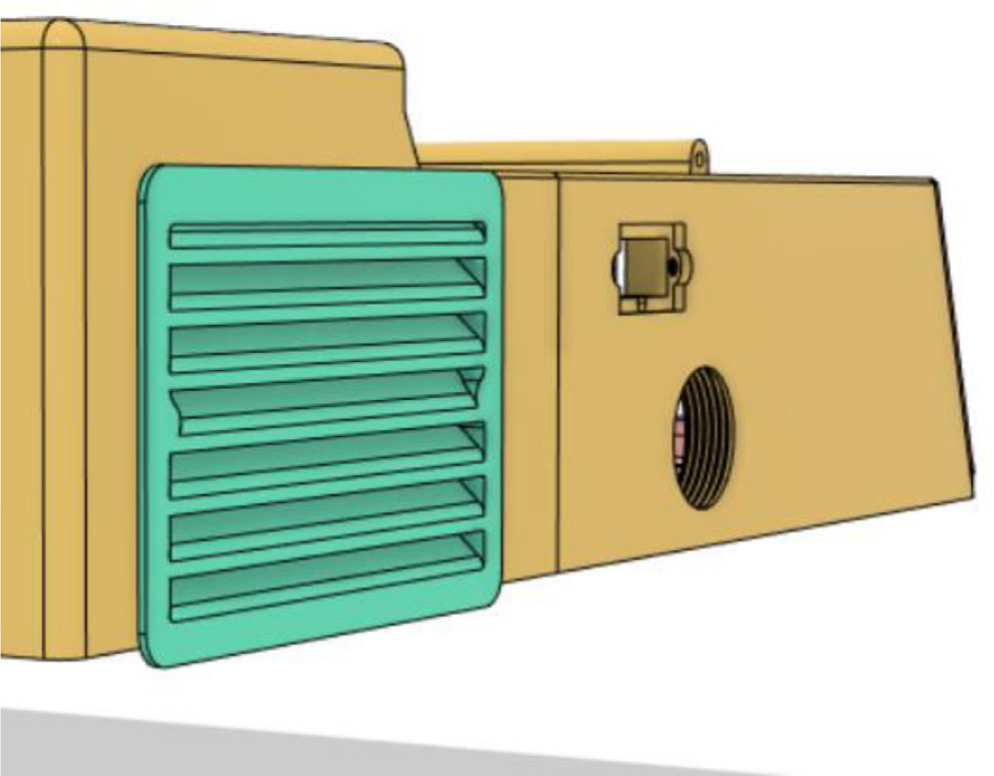
With side vent.
9. Glue the base of the LCD Holder Parts (1 and 3) with the Top 12 (see Fig. 39, Fig. 40)

**Fig. 39:**
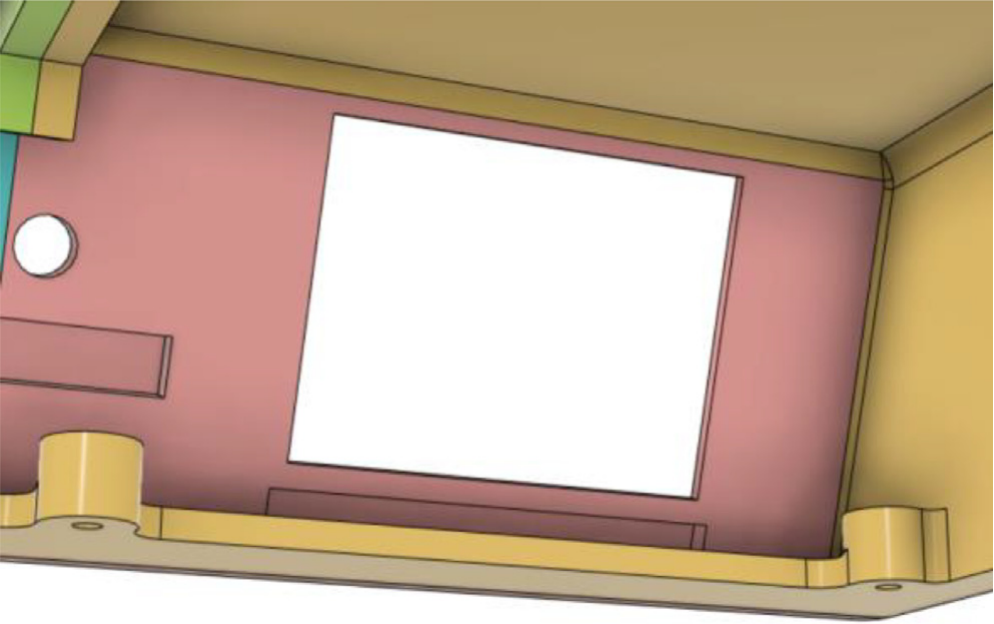
Without LCD Holder.

**Fig. 40:**
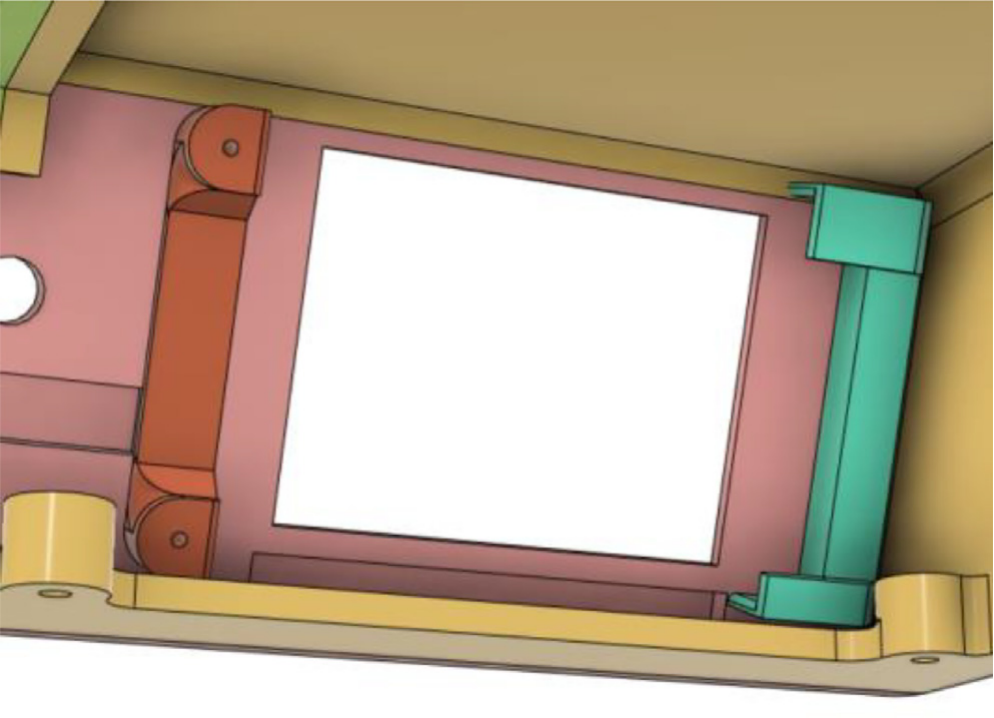
With LCD Holder.
10. Glue the Tab Covers: Front 11 (a and b) and Front 12 (a and b) (see Fig. 41)

**Fig. 41:**
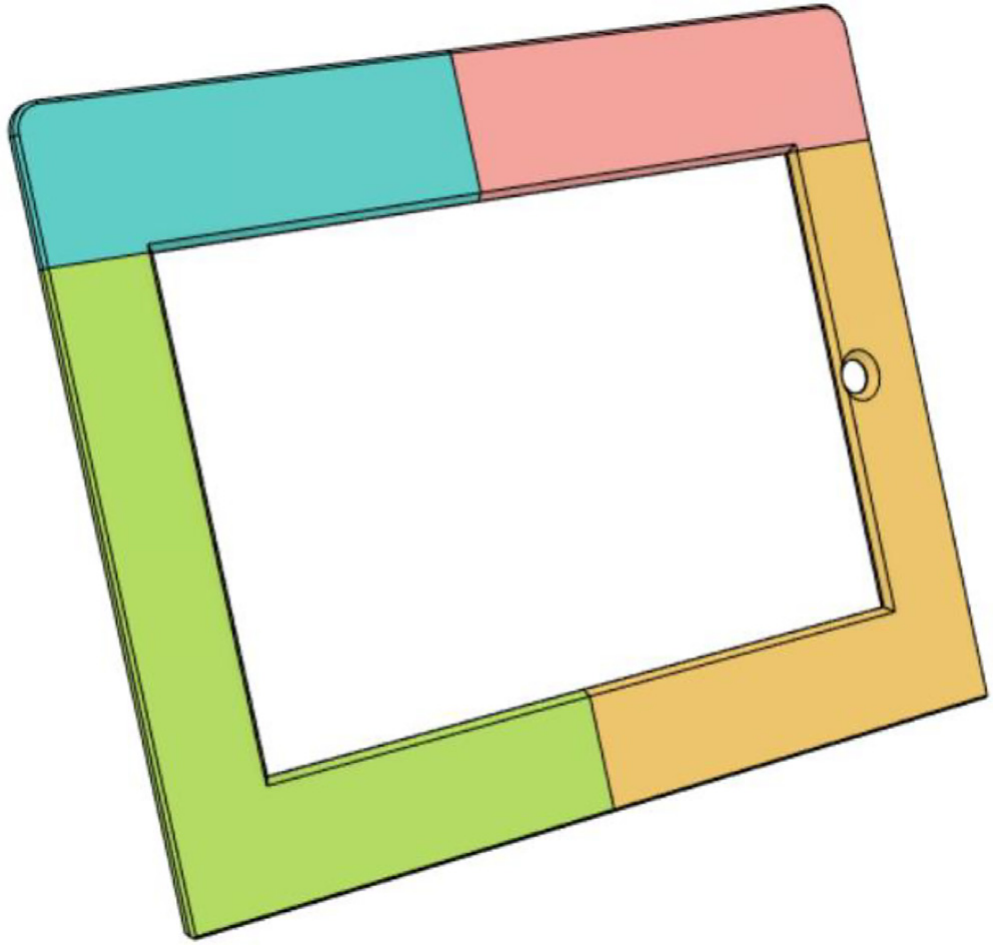
Front 11 and 12 are glued together.
11. Glue the middle edge Tab Covers: Back 11 and 12 (see Fig. 42)

**Fig. 42:**
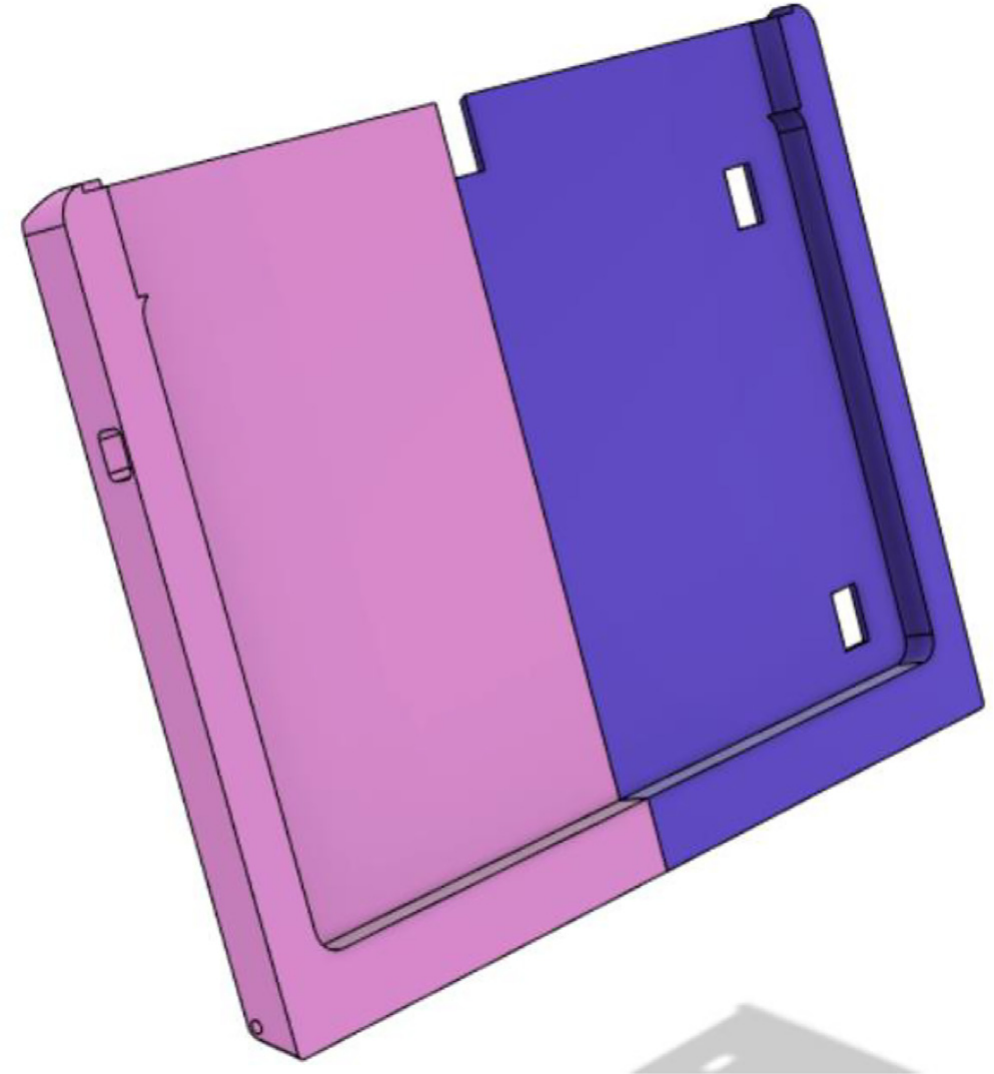
Back 11 and 12.
12. Glue the edges of the Tab Covers: Front and Back parts (see Fig. 43)

**Fig. 43:**
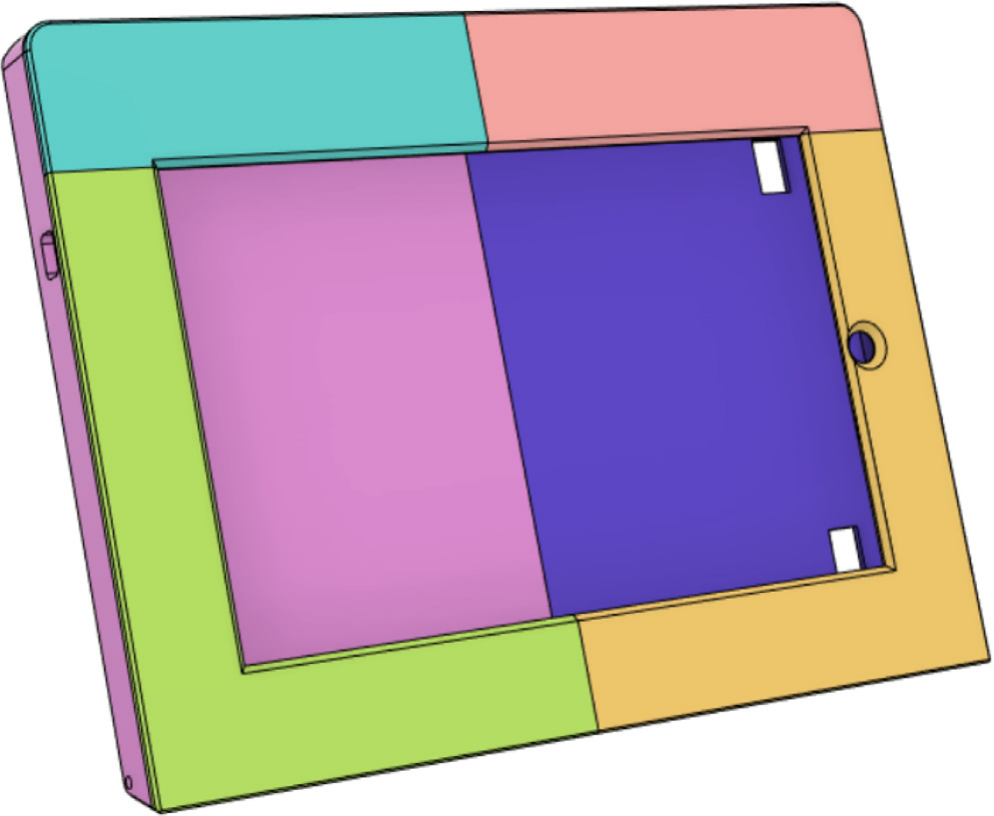
Front and Back Tab Covers.
13. Glue the edges of the Tab Cover Top parts (1 and 2) (see Fig. 44)

**Fig. 44:**
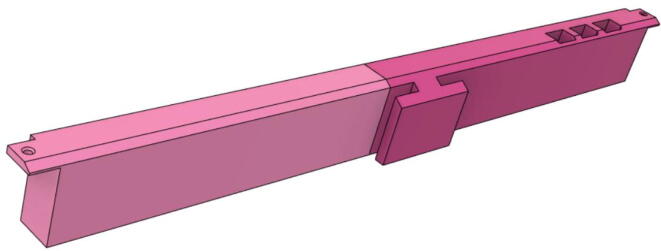
Tab Cover Top parts (1 and 2).
14. Let the glue dry and set the pieces together
15. Install the Power Socket with Base 22, Switch DPDT and DC Power Socket with Top 21 and Debug Cover with Top 22 (see Fig. 45, Fig. 46)

**Fig. 45:**
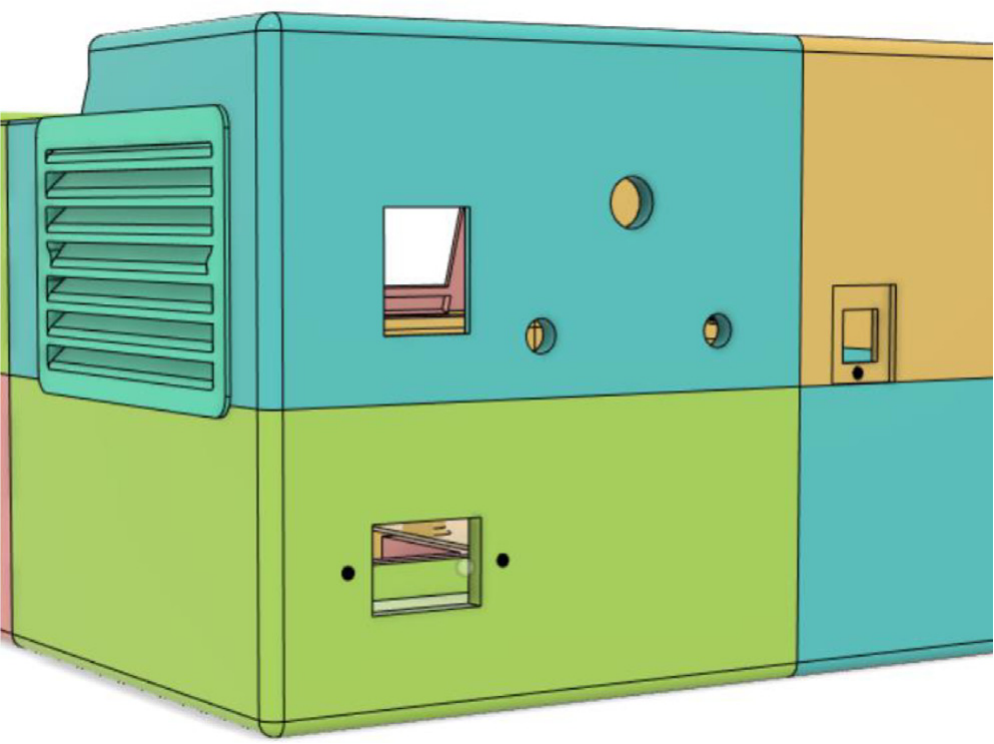
Rear view without ports and sockets.

**Fig. 46:**
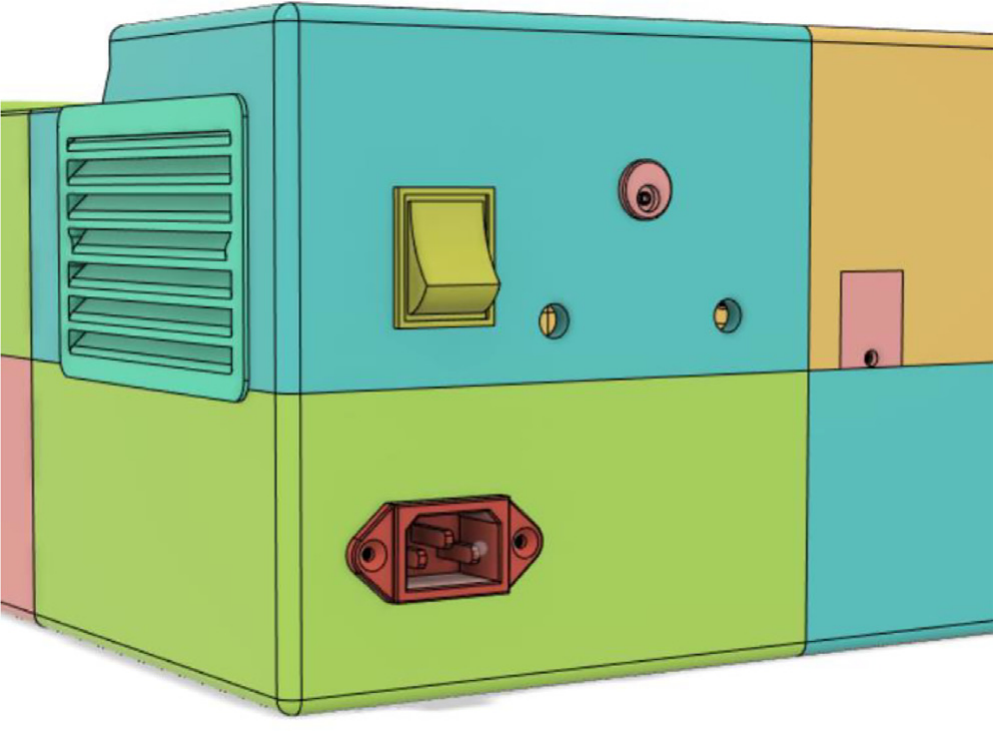
Rear view with installments.
16. Slide in the ‘2.8 TFT LCD Display’ into the LCD Holder 1 and then place LCD Holder 2 on the top left of the display and screw it into place (see Fig. 47, Fig. 48)

**Fig. 47:**
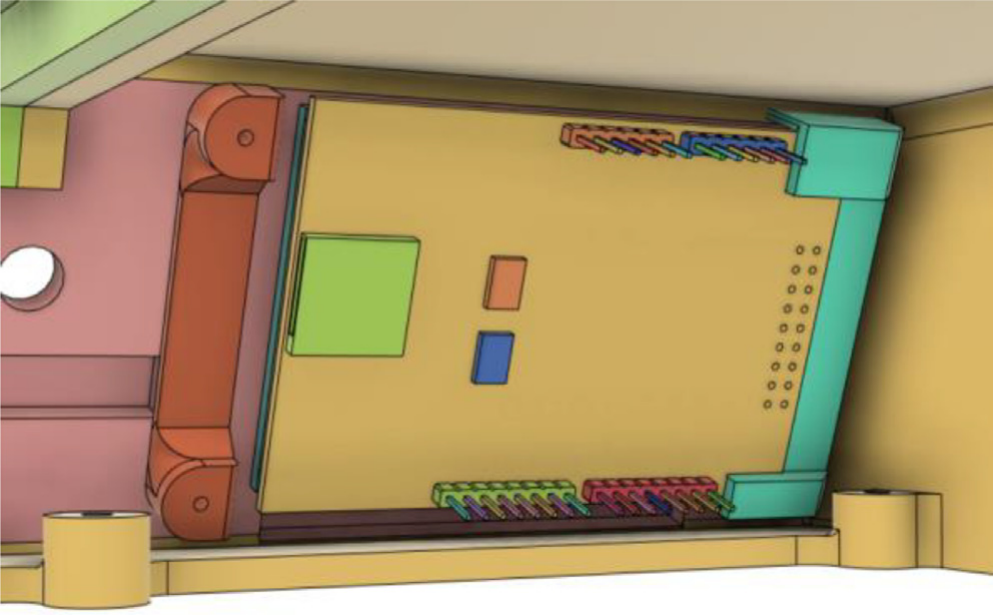
LCD slid into place.

**Fig. 48:**
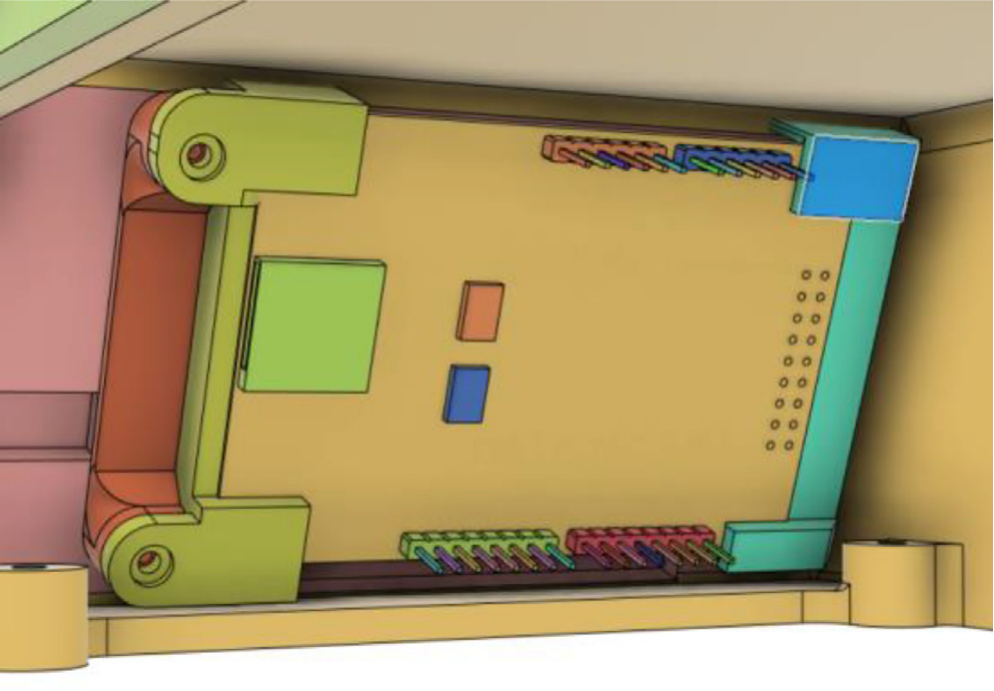
LCD Holder 2 on the top left of the display, ready to be screwed into place.
17. Install the ‘Rotary Encoder’, ‘Knob 20 mm’, ‘Indicator’, ‘Push Button’ and ‘DMX 5 pin socket’ with the Top 11 and 22 (see Fig. 49, Fig. 50, Fig. 51)

**Fig. 49:**
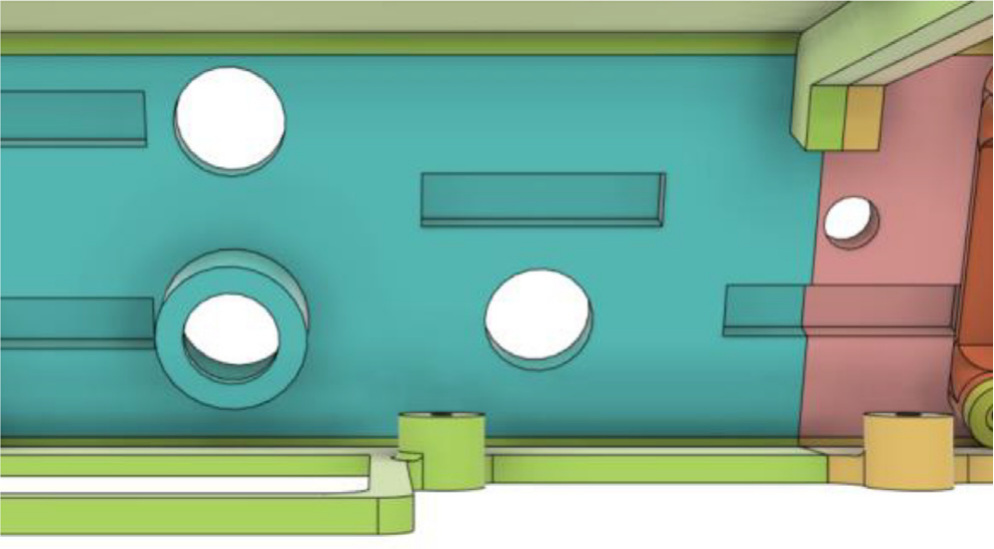
Without parts.

**Fig. 50:**
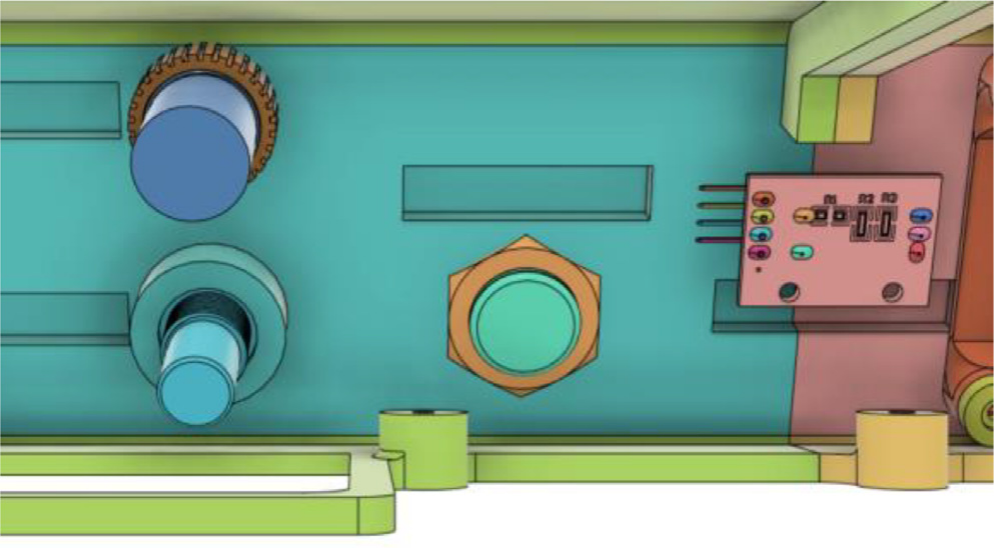
Installed parts (rear view).

**Fig. 51:**
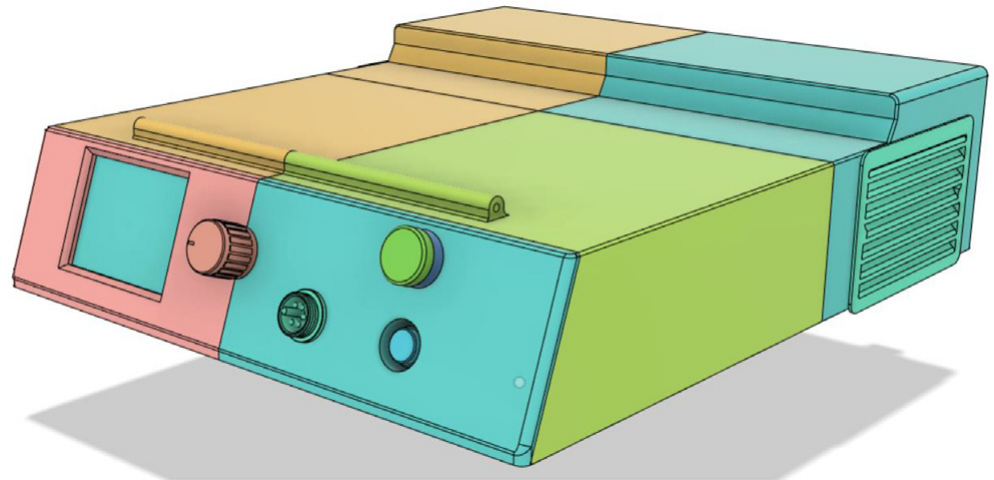
Installed parts (Front view).
18. Two 3 mm Linear Rods have been inserted into the Hinge part of Back11 and Back12 from the opposite sides for the Tab Cover to rotate about the linear rod's axis (see Fig. 52, Fig. 53)

**Fig. 52:**
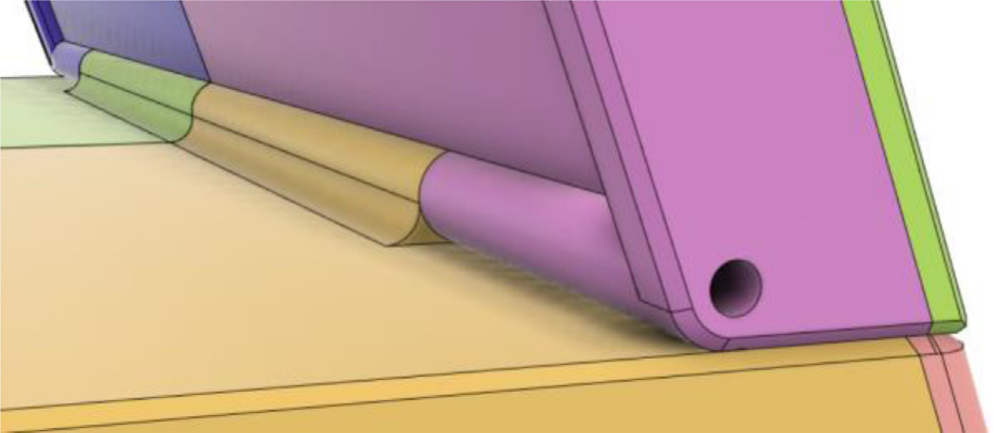
Without 3 mm linear rods.

**Fig. 53:**
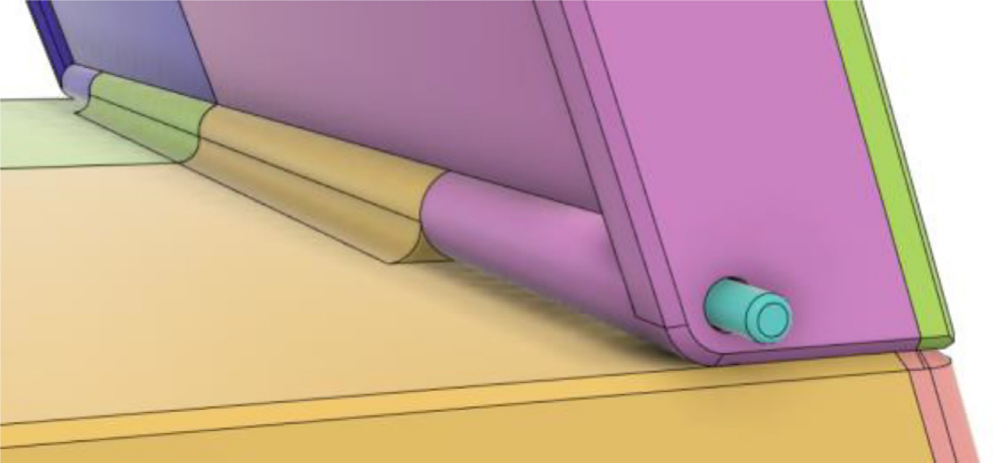
With 3 mm linear rods.
19. Insert the Tab into the Tab Cover Front and Back and place the buttons in the specific places and then screw down the Tab Cover Top (see Fig. 54, Fig. 55)

**Fig. 54:**
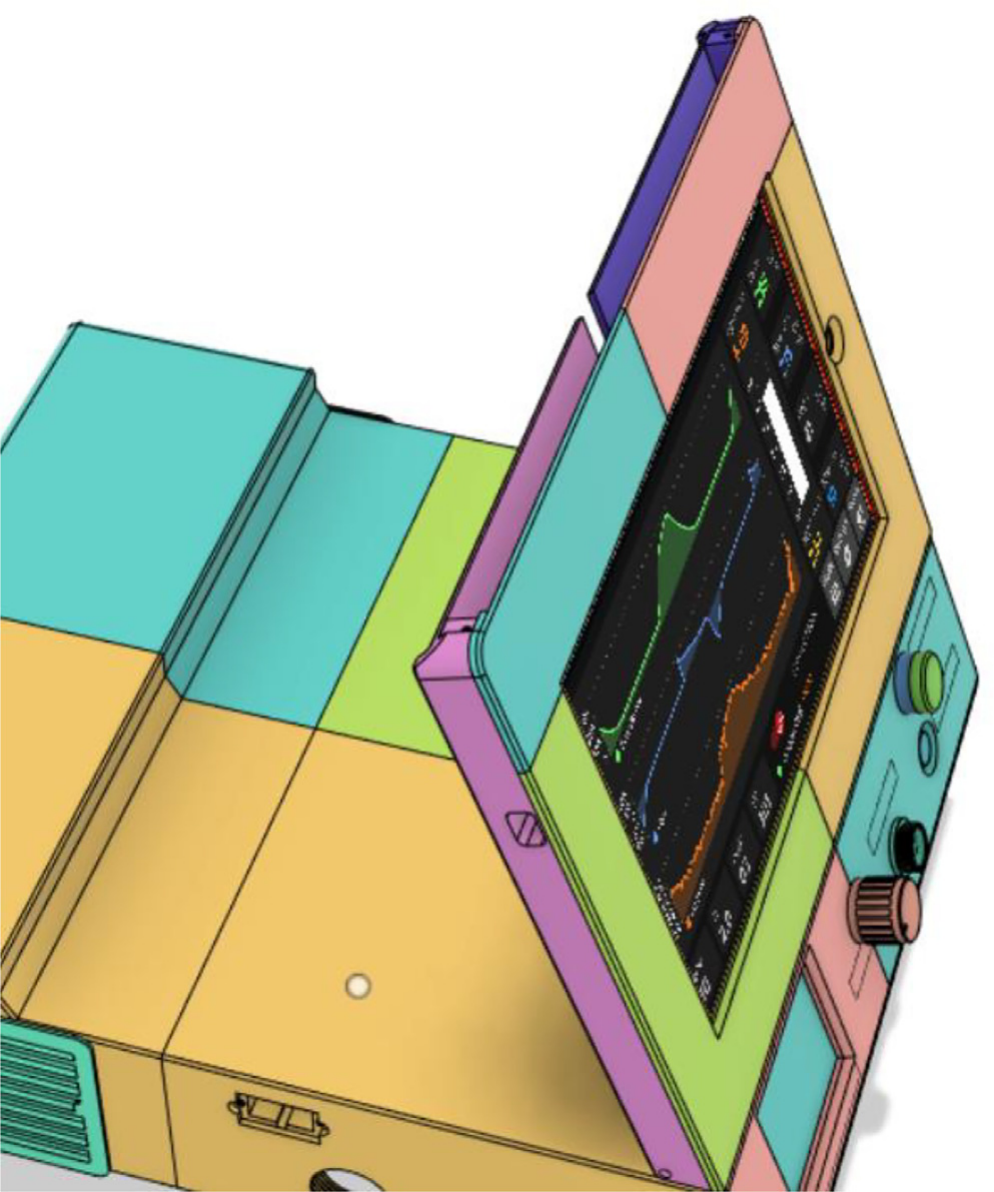
Tablet inserted into place.

**Fig. 55:**
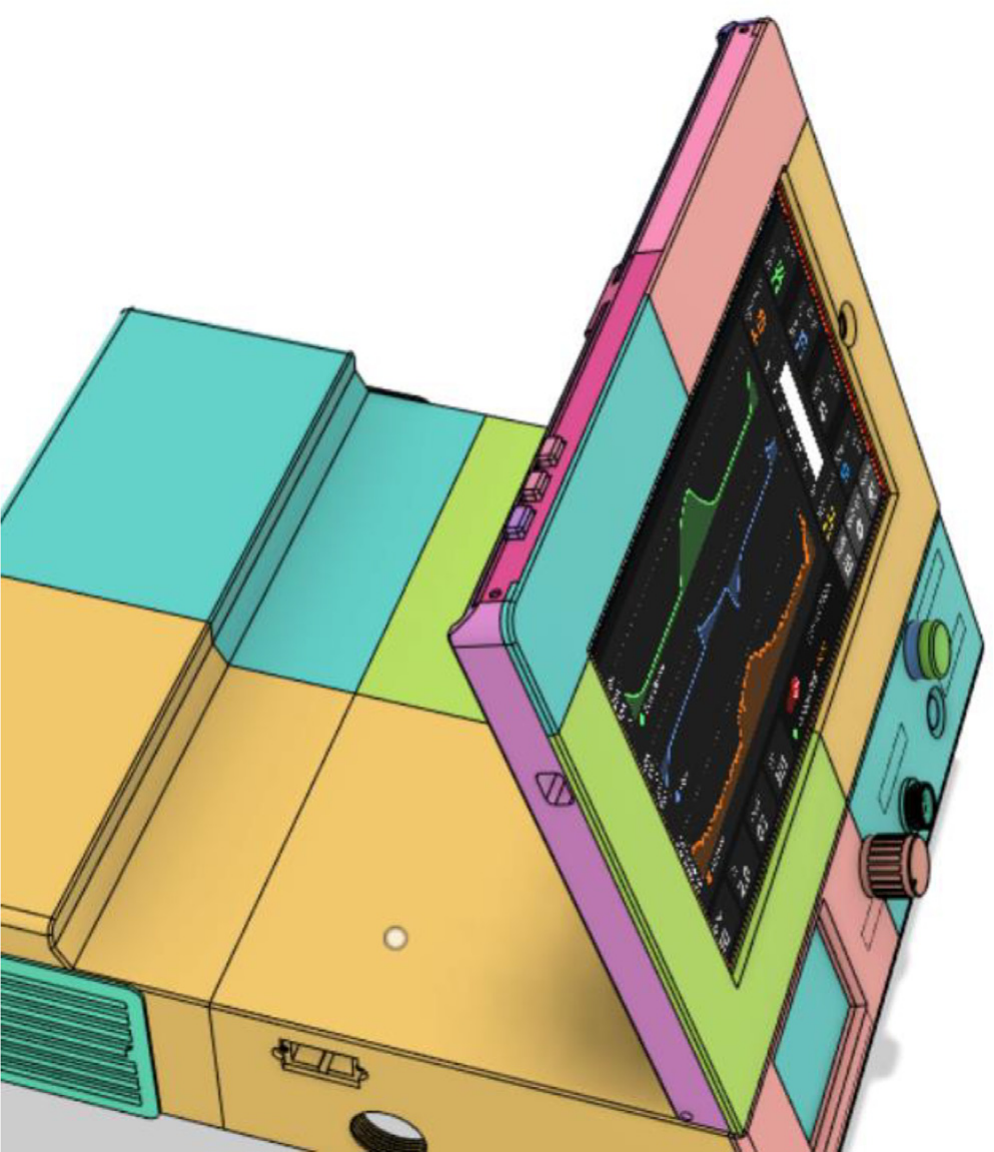
With Tab Cover Top attached to holder tablet into place.
20. Glue the Holder 2 with Top 11 and Top 21, Holder 1 with Back 11 and Holder 3 with Top 12 and Top 22 (see Fig. 56, Fig. 57).

**Fig. 56:**
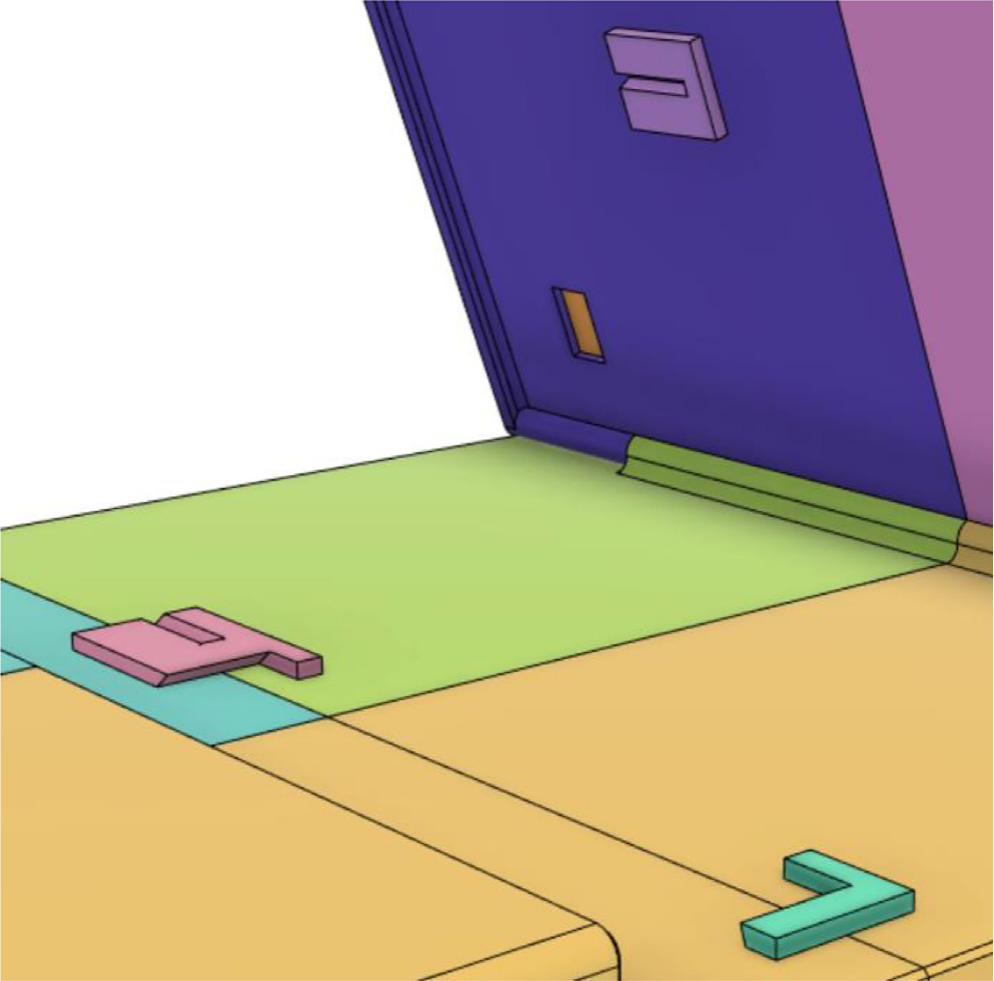
Holder 2 with Top 11 and Top 21, Holder 1 with Back 11 and Holder 3 with Top 12 and Top 22.

**Fig. 57:**
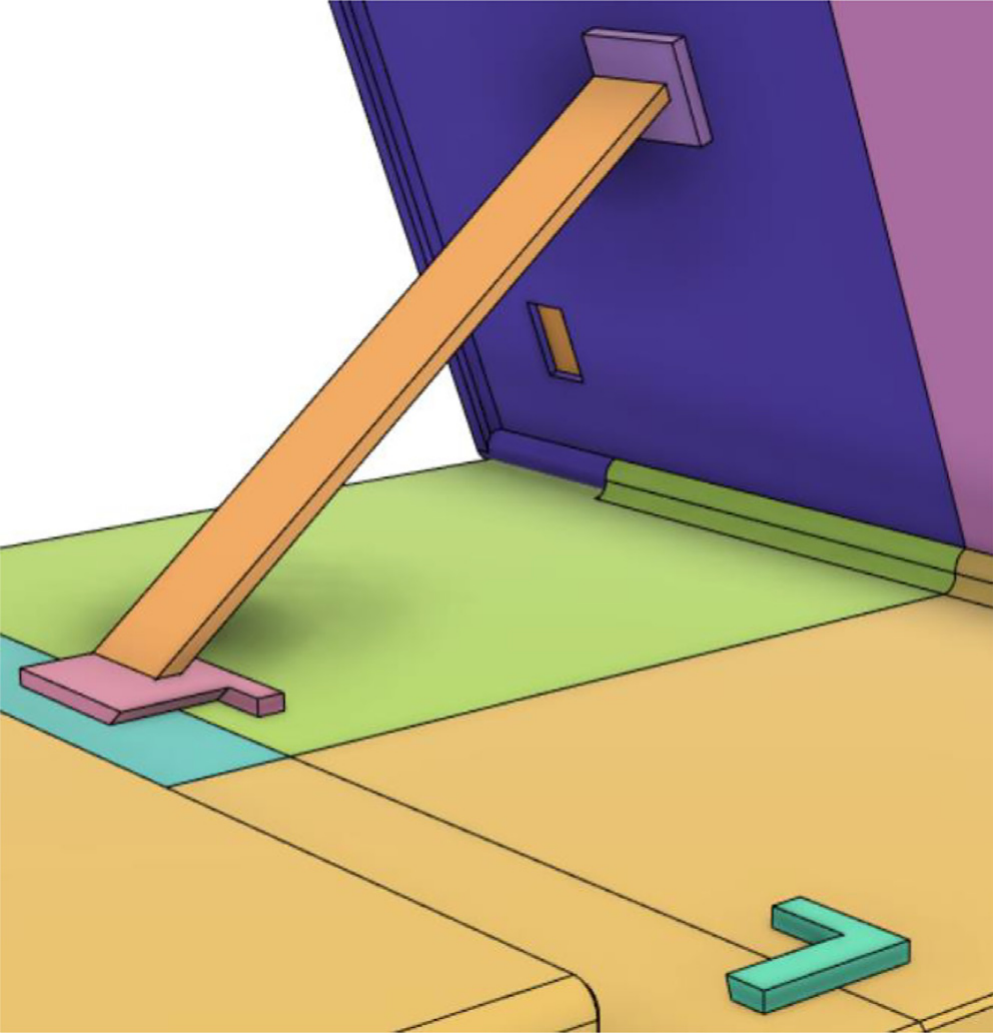
Support 1 placed in between Holder 1 and 2.
  Support 1 is used to hold the tablet at an angle for a better view of the display. Distance between Holder 1 and 2 and distance between 2 and 3 are kept the same so that the tablet support can be kept with the case when not in use.
21. Top Full Assembly (see Fig. 58, Fig. 59).

**Fig. 58:**
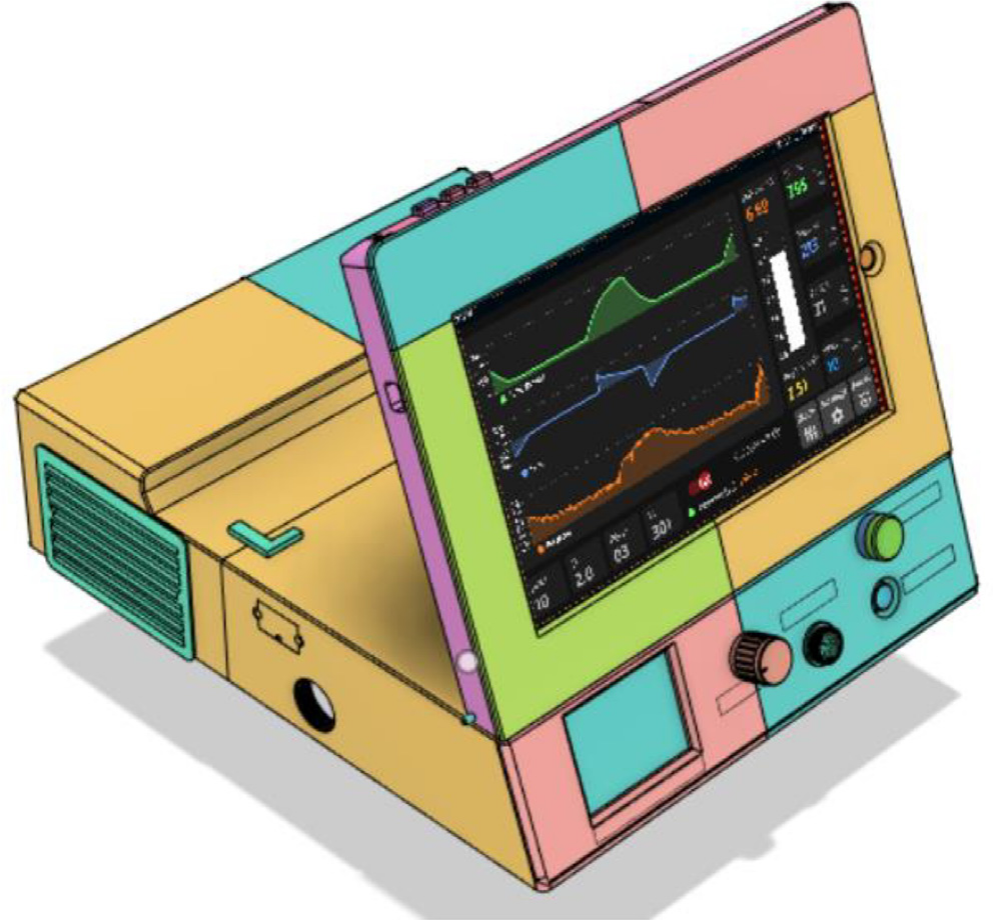
Top Assembly (Front View).

**Fig. 59:**
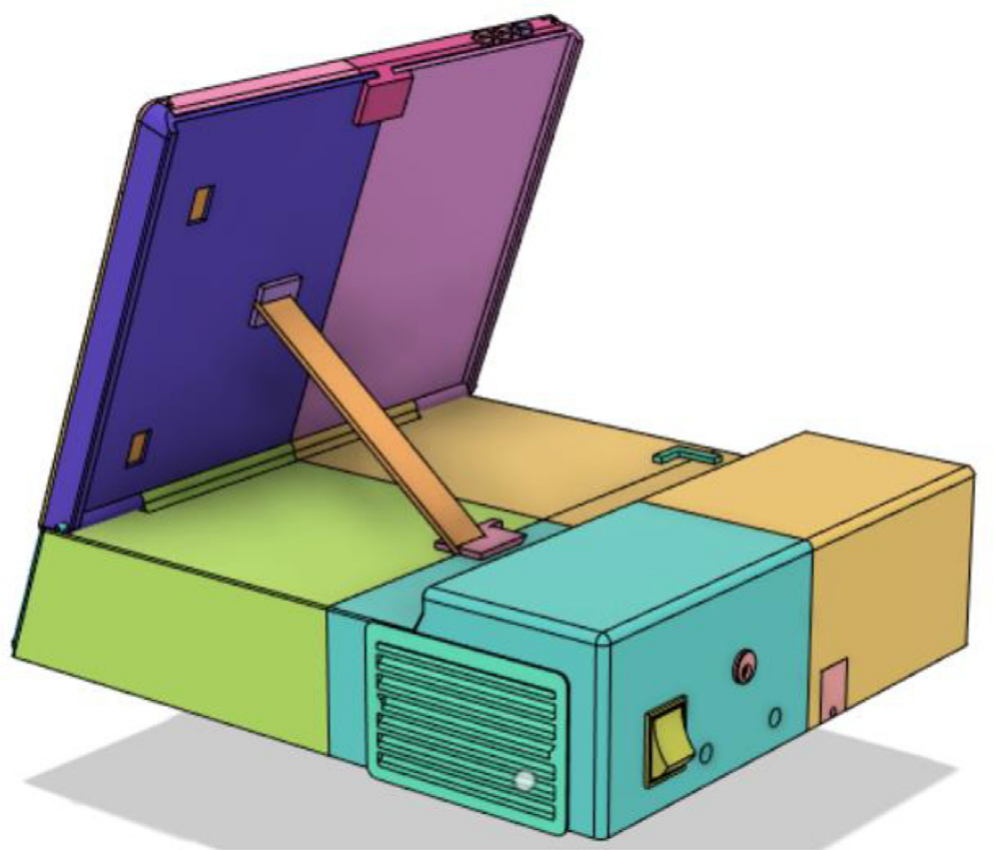
Top Assembly (Rear View).

### One-way Valve

**Fig. 60:**
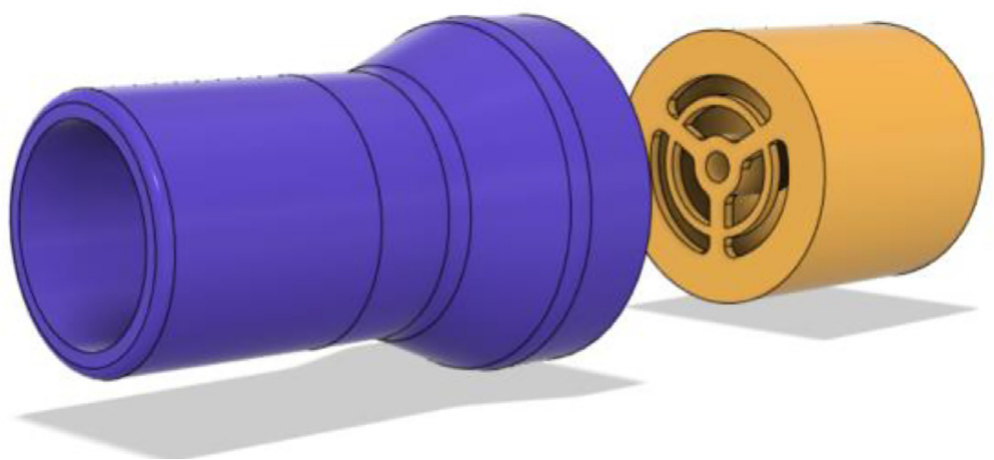
Parts before gluing them together.

1. Glue the flat base faces of One-way valve inlet and outlet (see Fig. 60, Fig. 61)

**Fig. 61:**
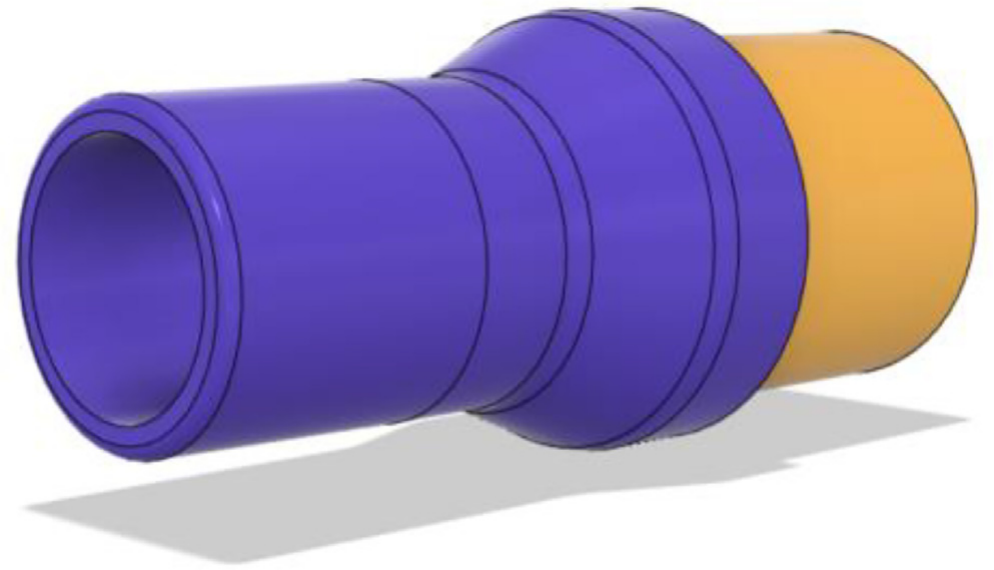
Parts after gluing them together.
2. Insert the Silicone Diaphragm through the Valve outlet side and place it on the mesh and screw it down (see Fig. 62, Fig. 63)

**Fig. 62:**
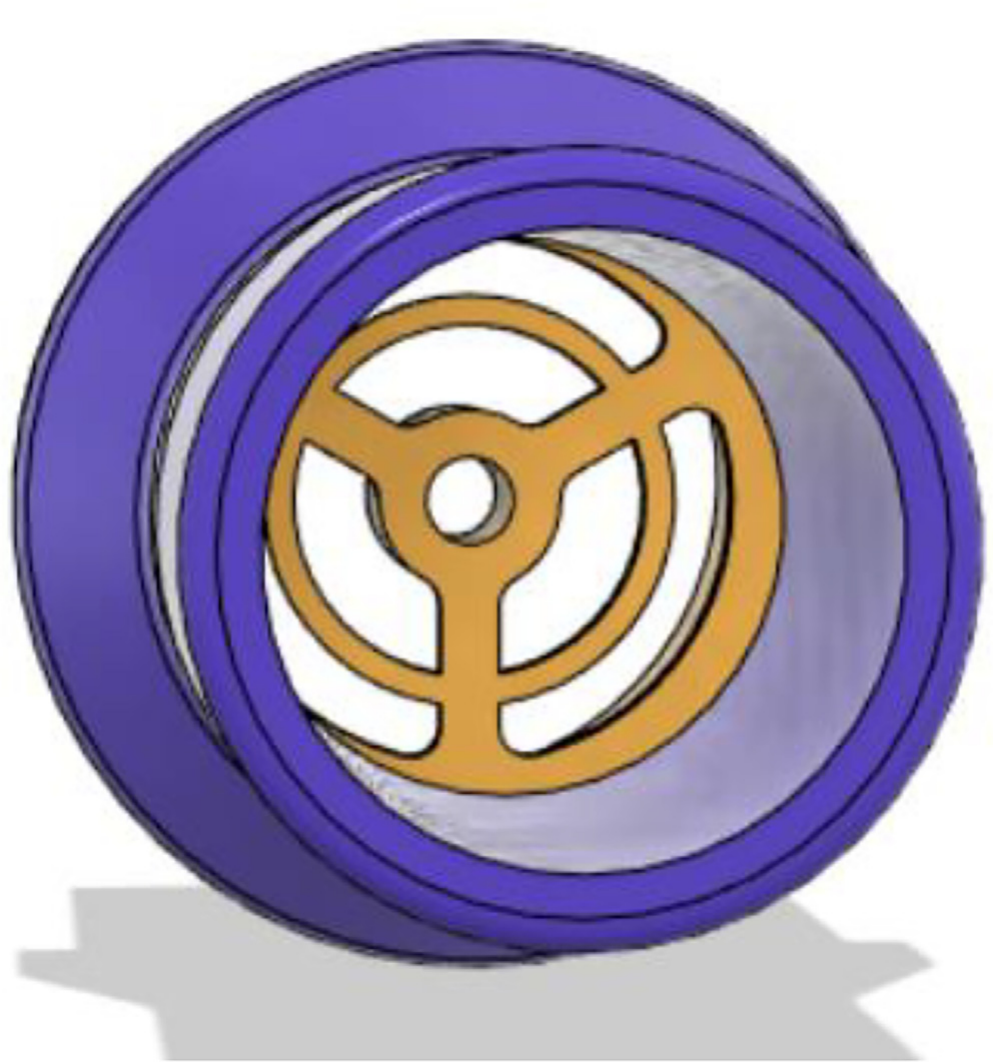
Before screwing in the diaphragm.

**Fig. 63:**
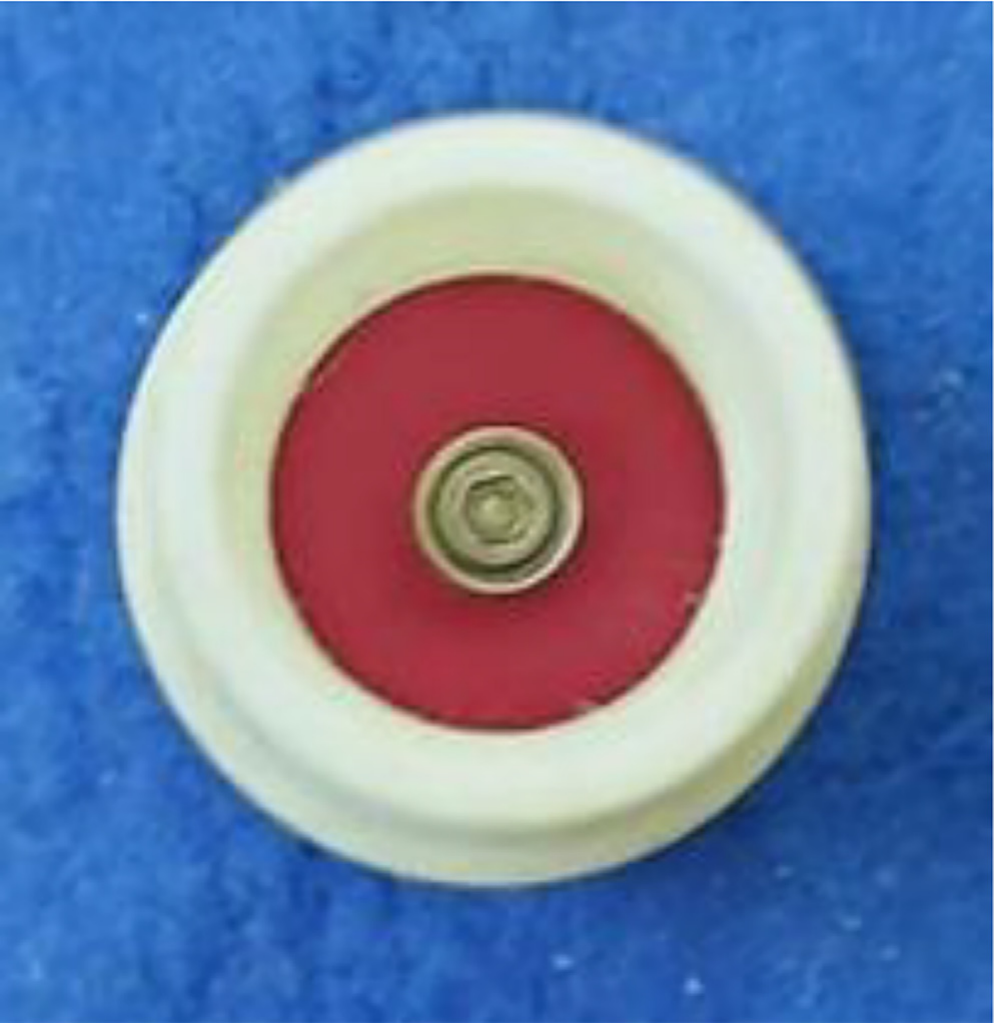
After screwing in the diaphragm.

### PEEP valve

**Fig. 64:**
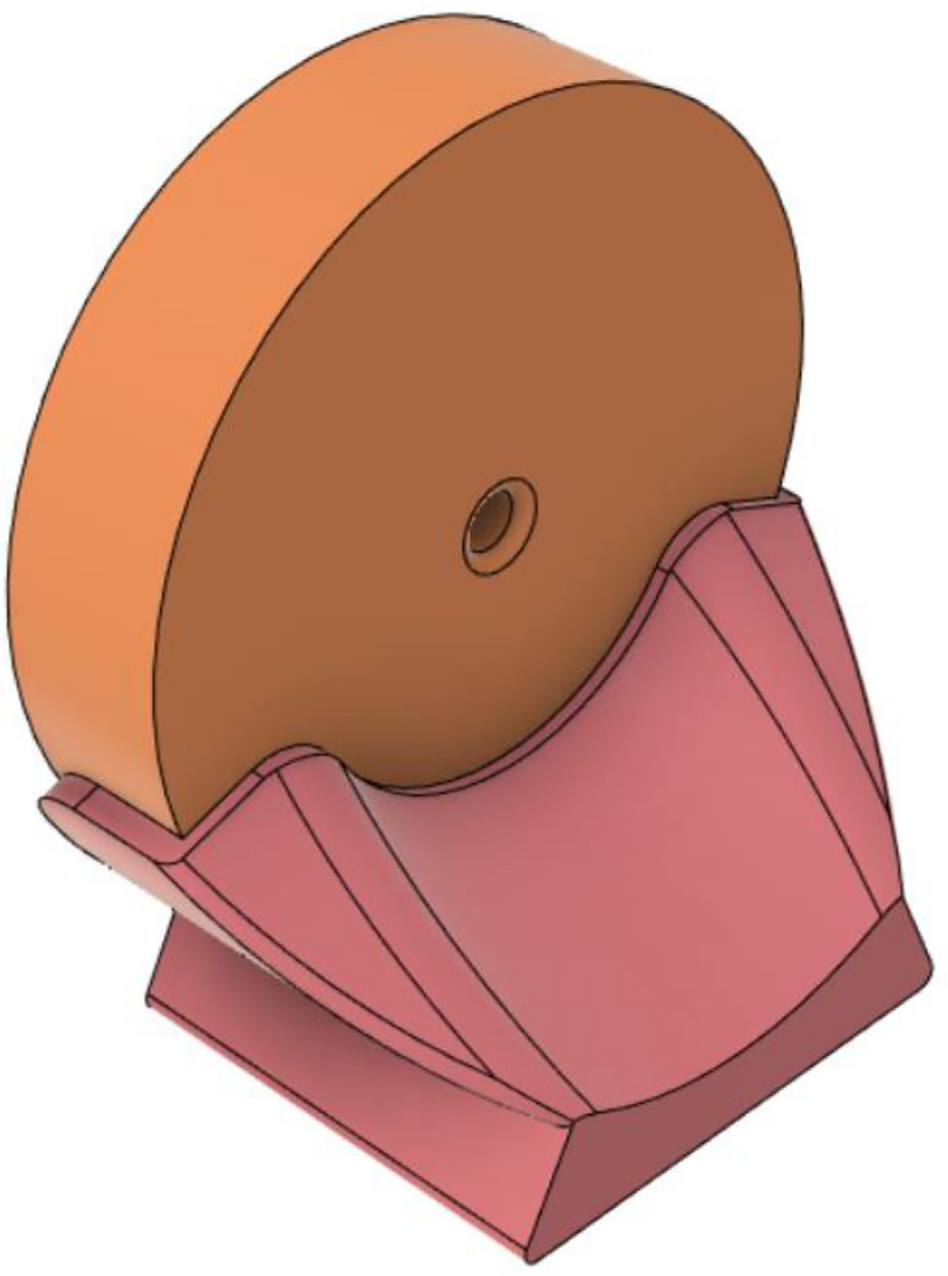
Gluing the outlet and attachment.

1. Glue the outer face of the outlet and the attachment (see Fig. 64, Fig. 65)

**Fig. 65:**
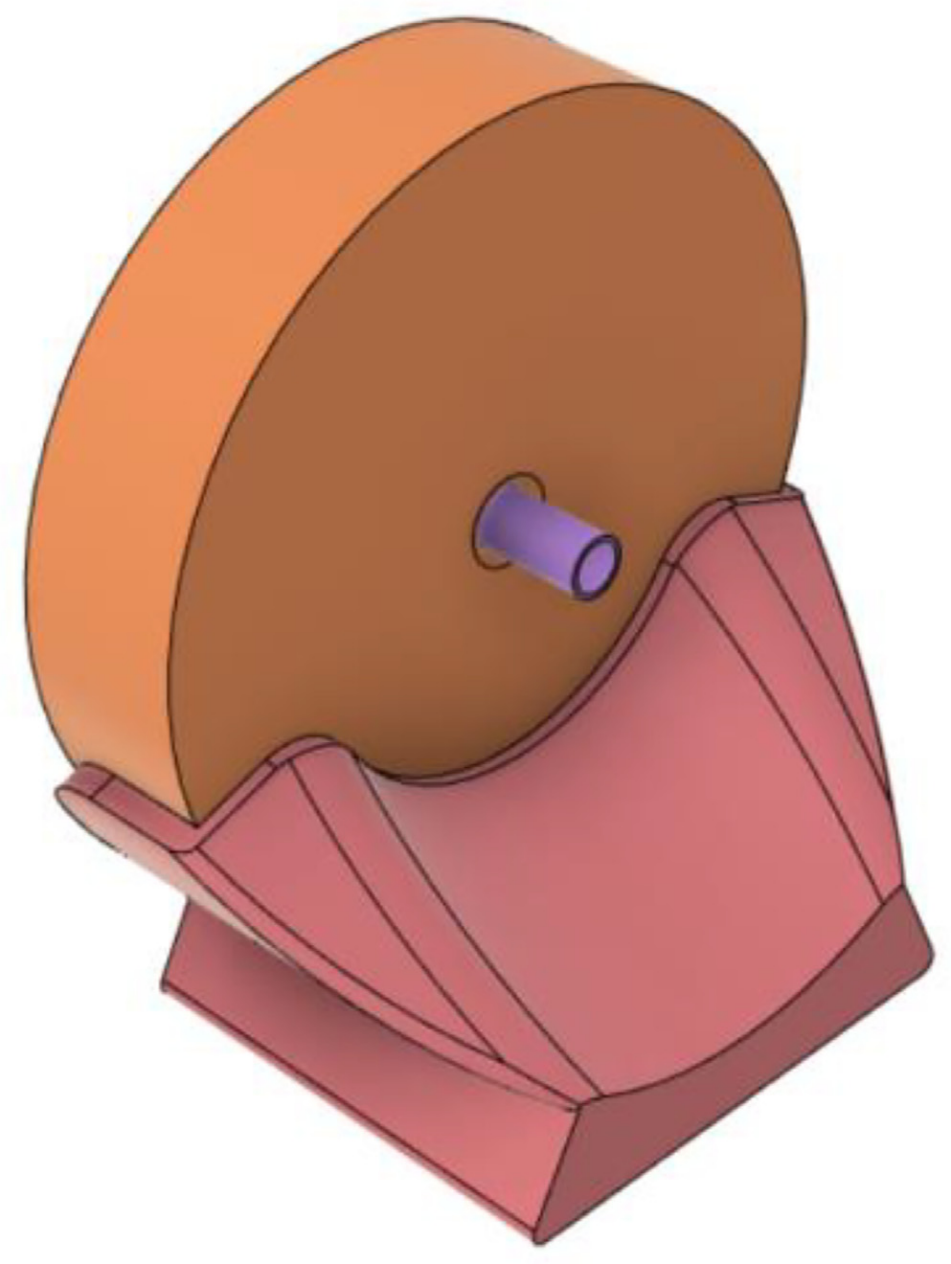
Attaching the Peep Extension with the outlet.
2. Place the Diaphragm on the Inlet and screw it (see Fig. 66, Fig. 67)

**Fig. 66:**
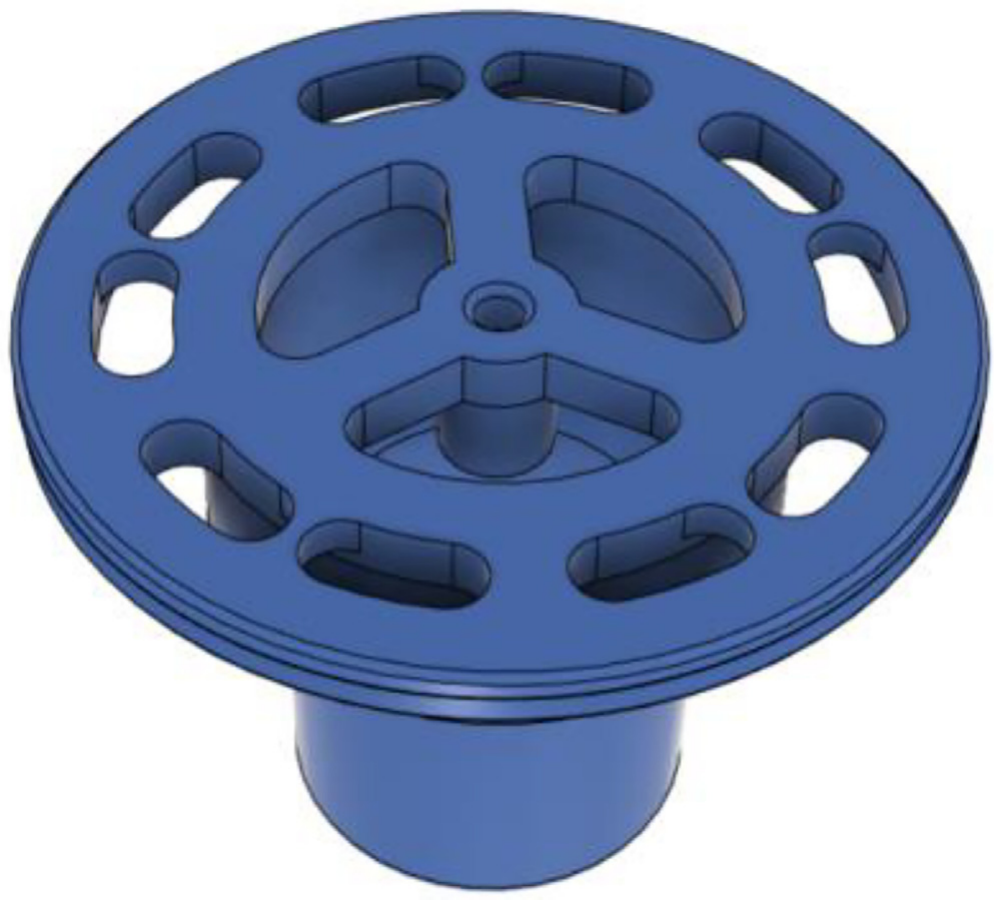
Before screwing the diaphragm.

**Fig. 67:**
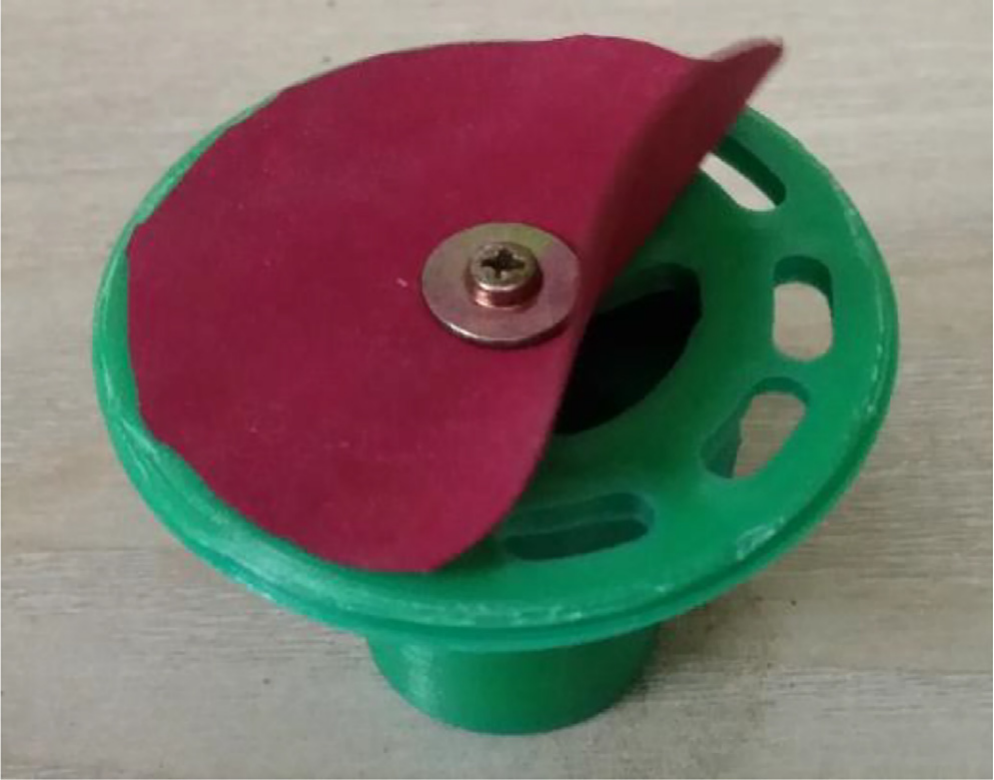
After screwing the diaphragm.
3. After the glue dries out, screw the Inlet and Outlet (see Fig. 68, Fig. 69)

**Fig. 68:**
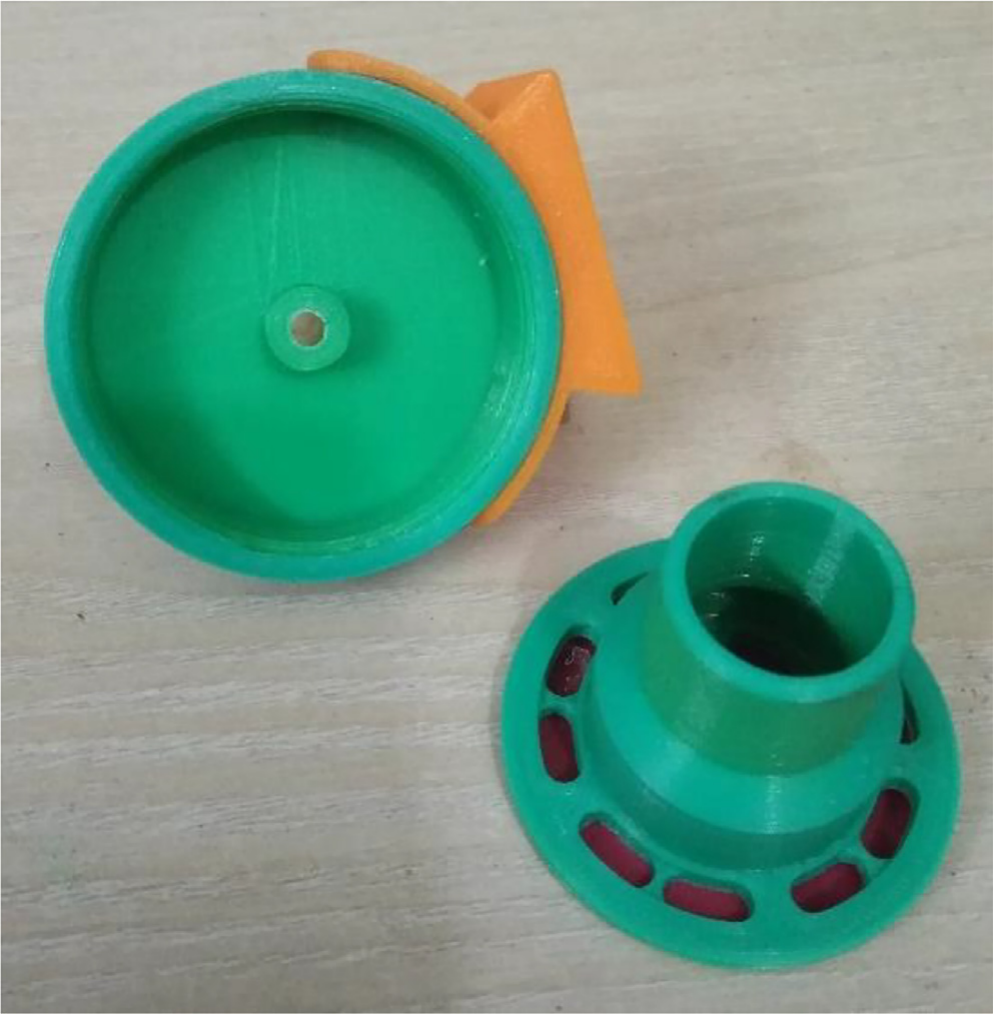
Before screwing the pieces together.

**Fig. 69:**
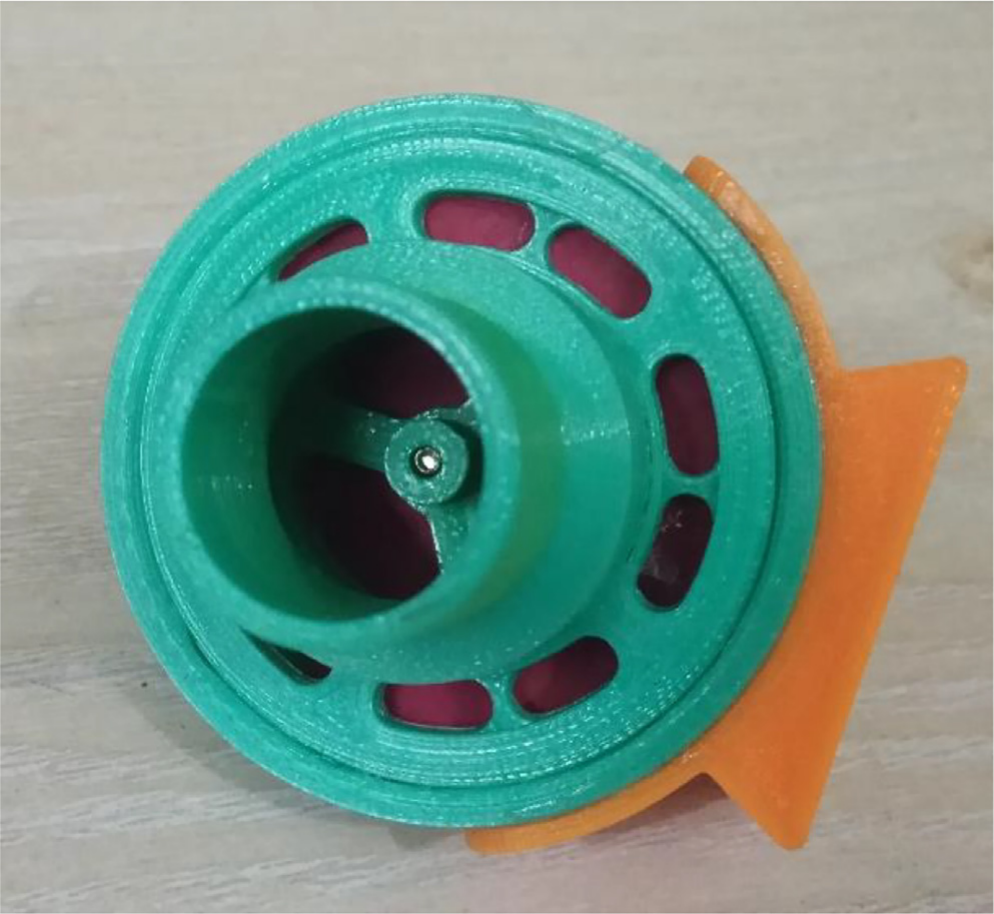
After screwing the parts.

### Pressure Release Mechanism (PRM).

**Fig. 70:**
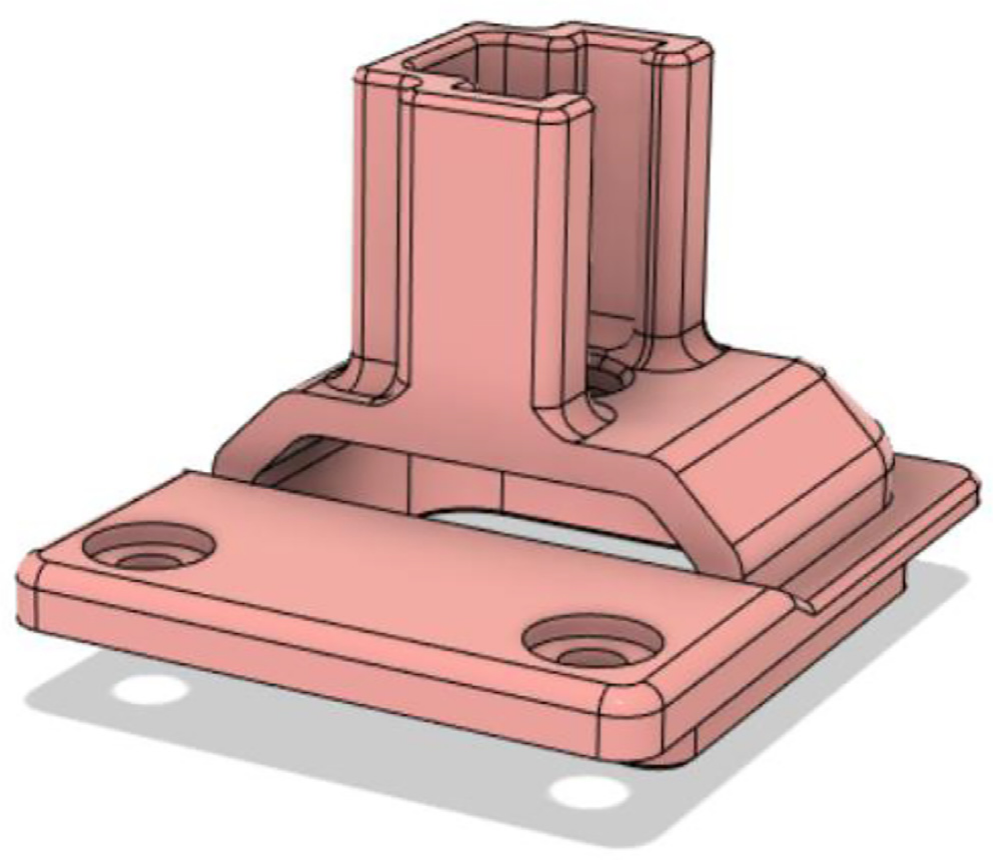
EOM (Base).

1. Glue the base of the EOM (Servo Bracket) and Base (see Fig. 70, Fig. 71)

**Fig. 71:**
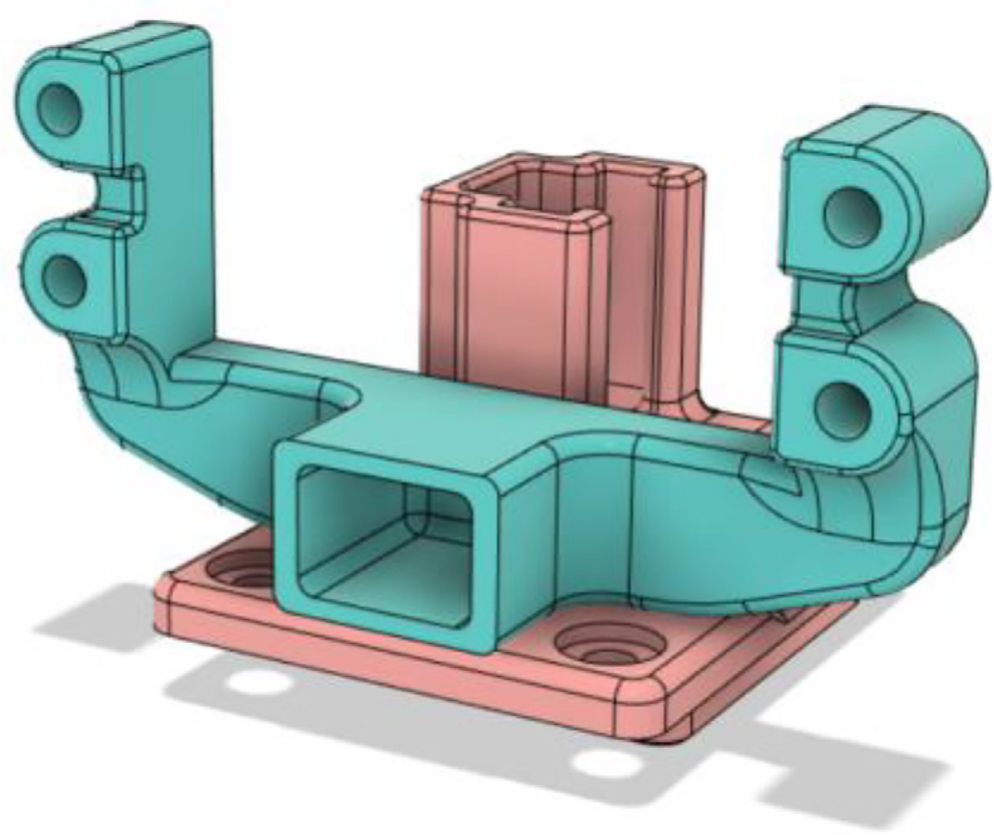
EOM (Servo Bracket) glued to EOM (Base).
2. Glue the edges of the exhaust valve gate and Outlet Body (see Fig. 72, Fig. 73)

**Fig. 72:**
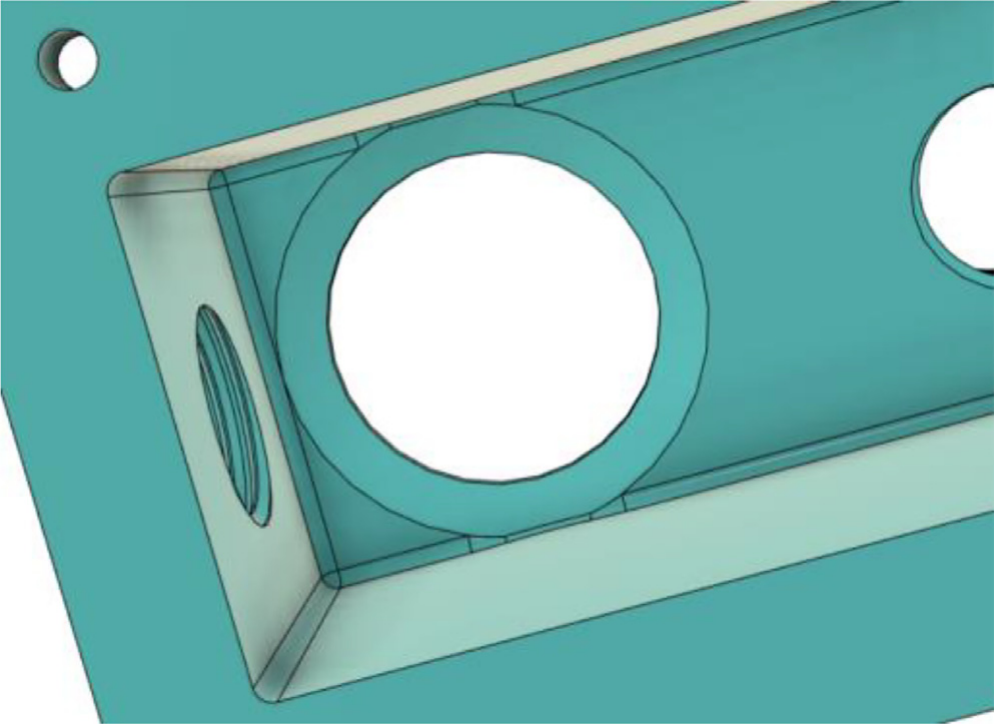
Outlet Body.

**Fig. 73:**
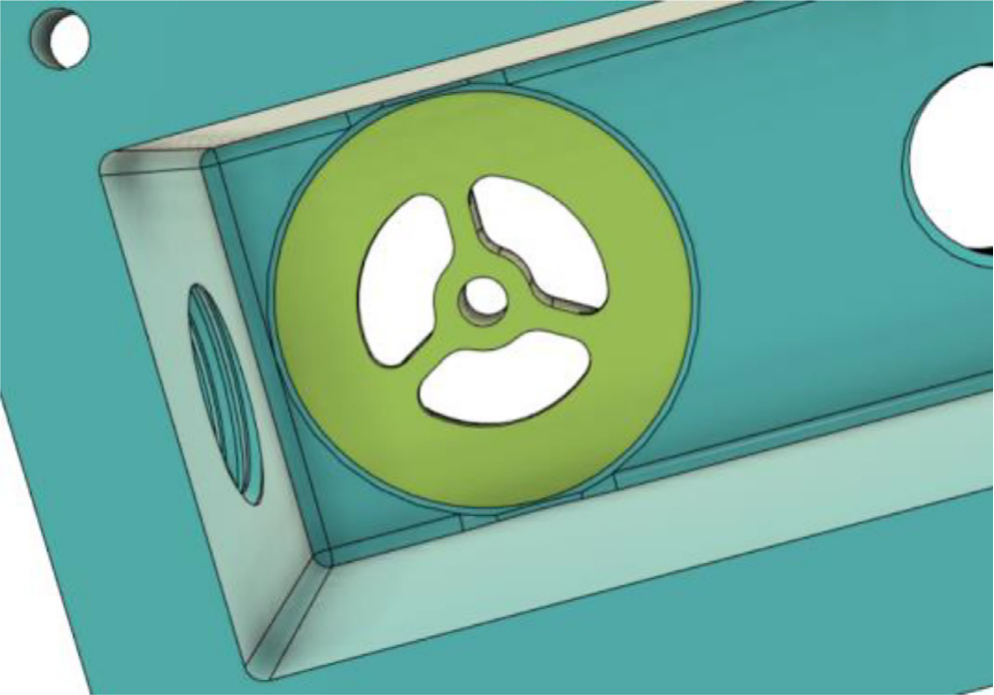
Exhaust Valve Gate glued to the Outlet Body.
3. Glue the Screw and EGV Base and then glue the Silicone Seal with the EGV Base (see Fig. 74, Fig. 75)

**Fig. 74:**
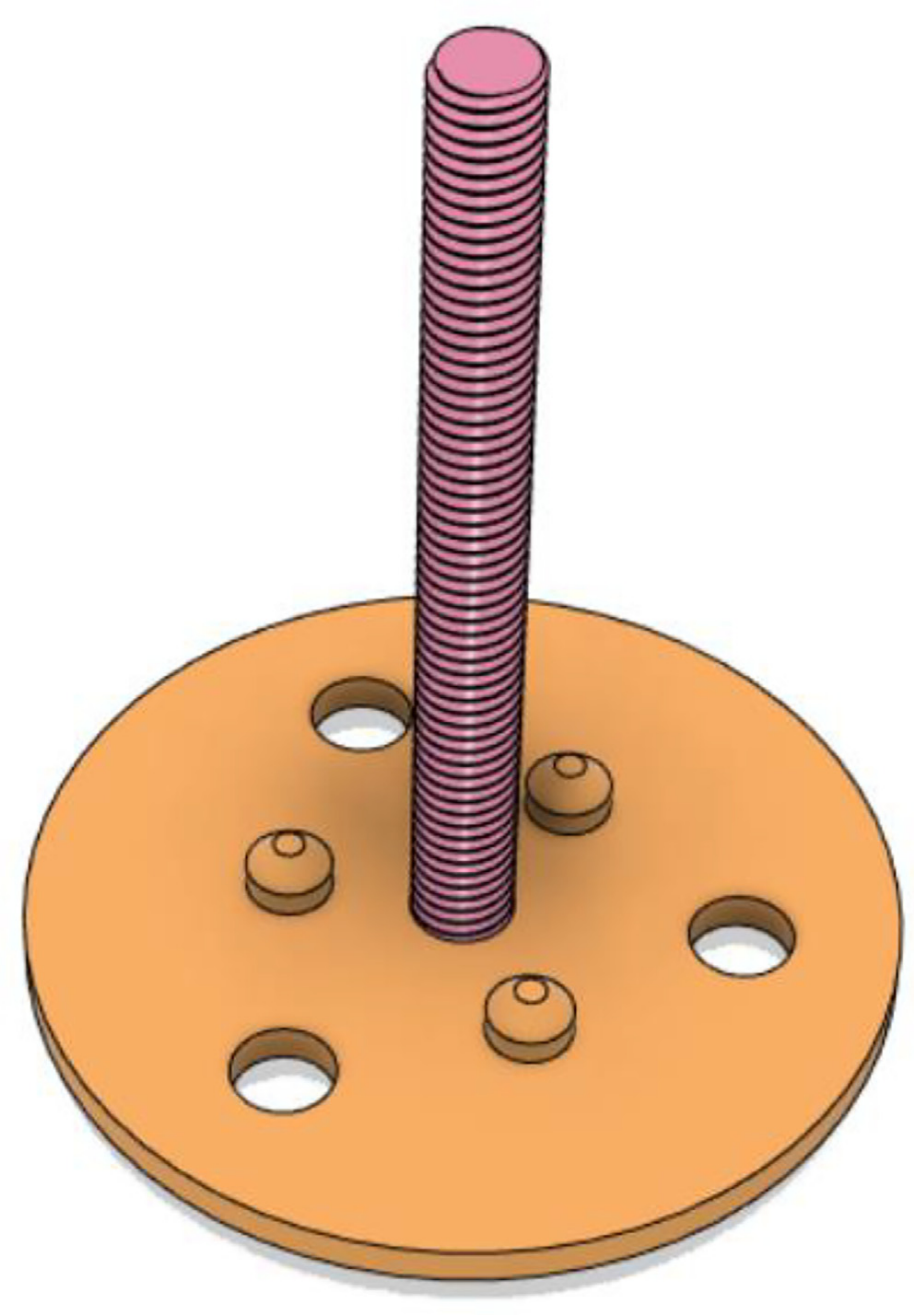
Screw and EGV Base glued together.

**Fig. 75:**
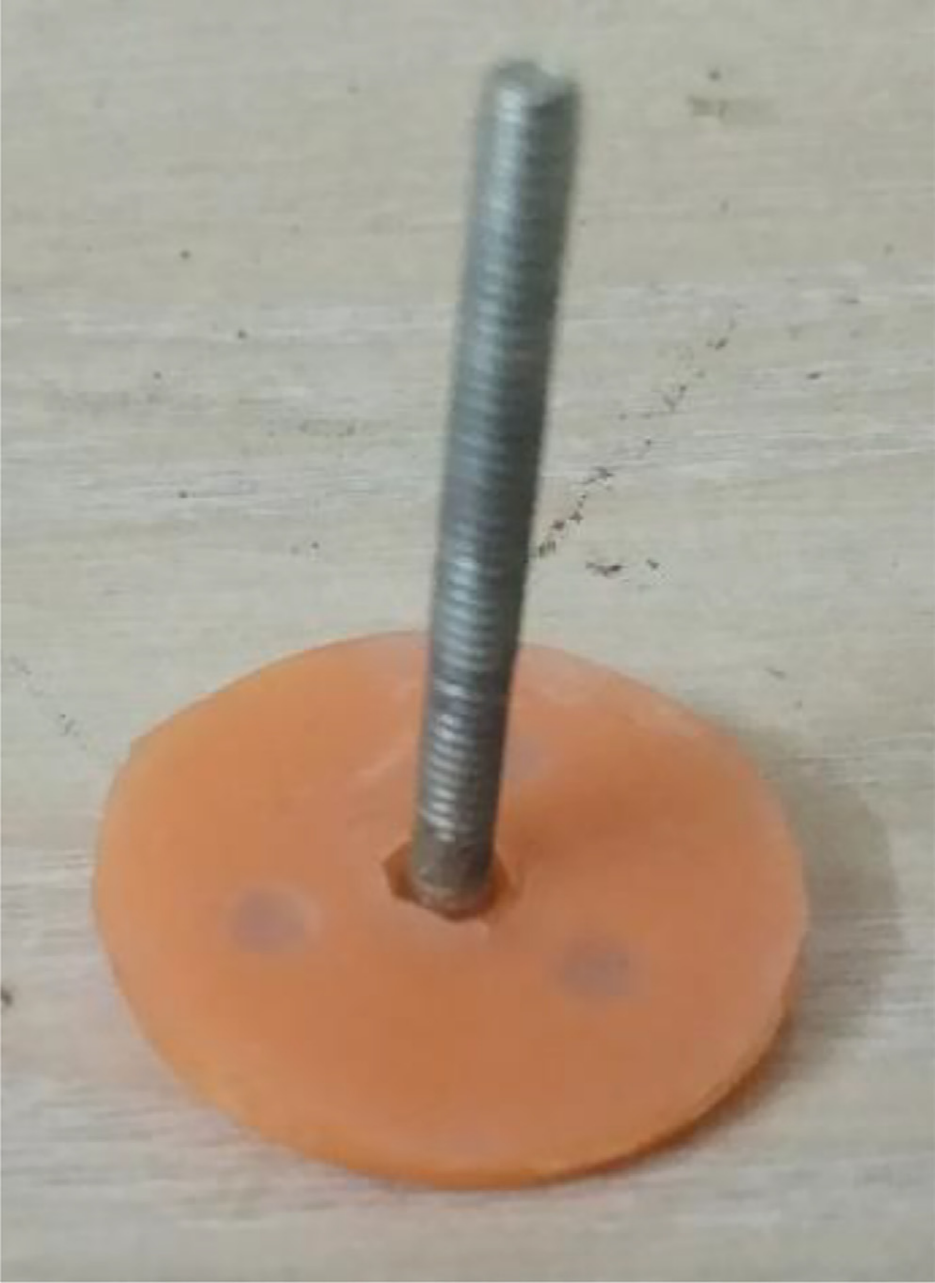
Silicone attached with the EGV Base.
4. Place the Stopper Gasket with the Stopper Bolt and screw it with the Outlet Body (see Fig. 76, Fig. 77)

**Fig. 76:**
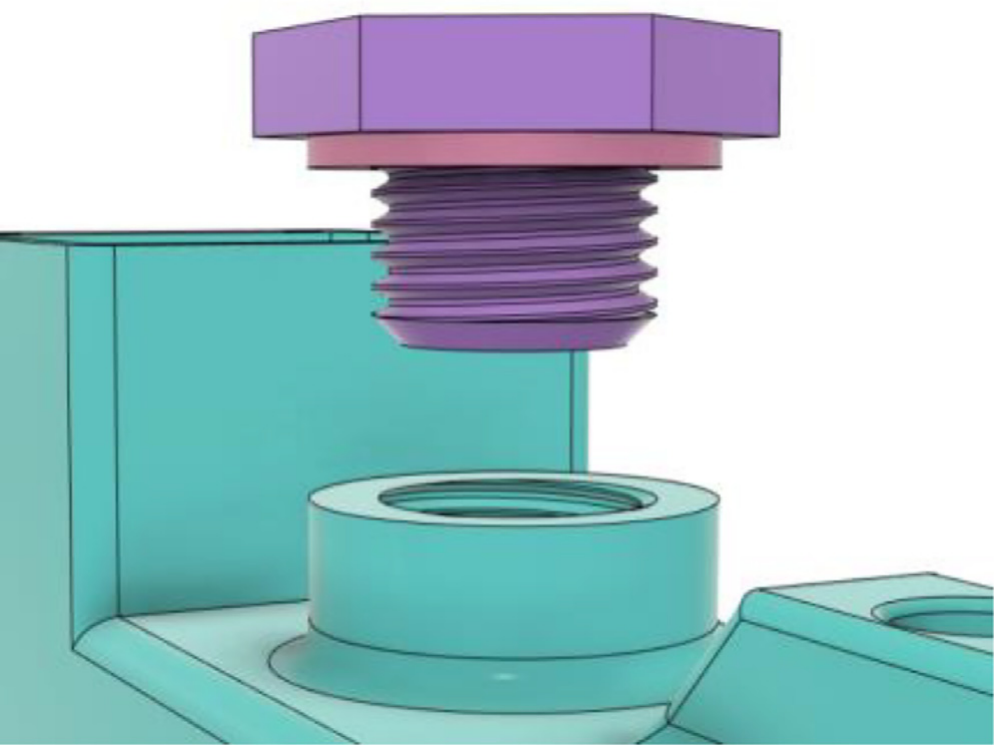
Place the Stopper Gasket on the Stopper Bolt.

**Fig. 77:**
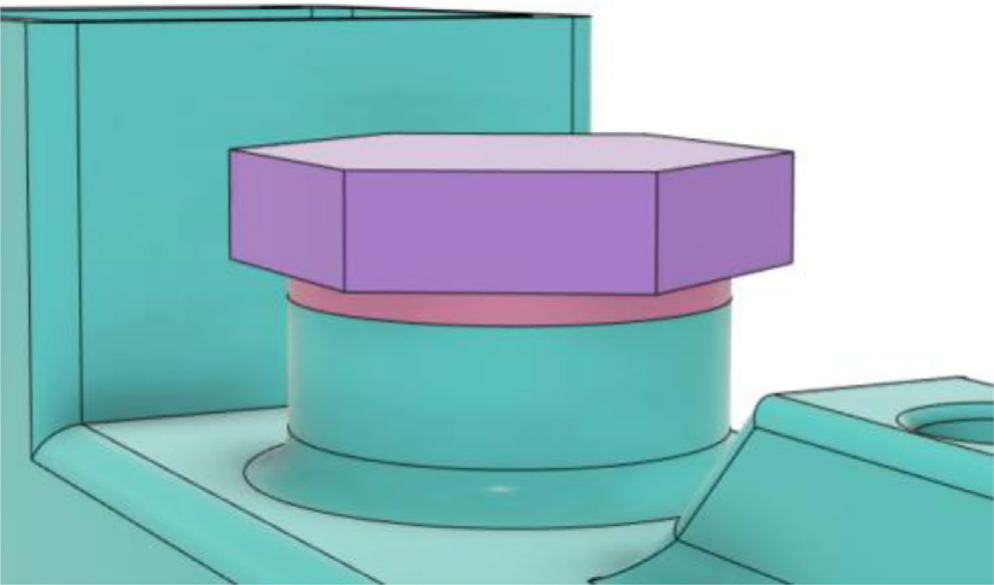
Screw the Stopper with the Outlet Body.
5. Screw in the 14 mm Adapters to the Outlet Body (see Fig. 78, Fig. 79)

**Fig. 78:**
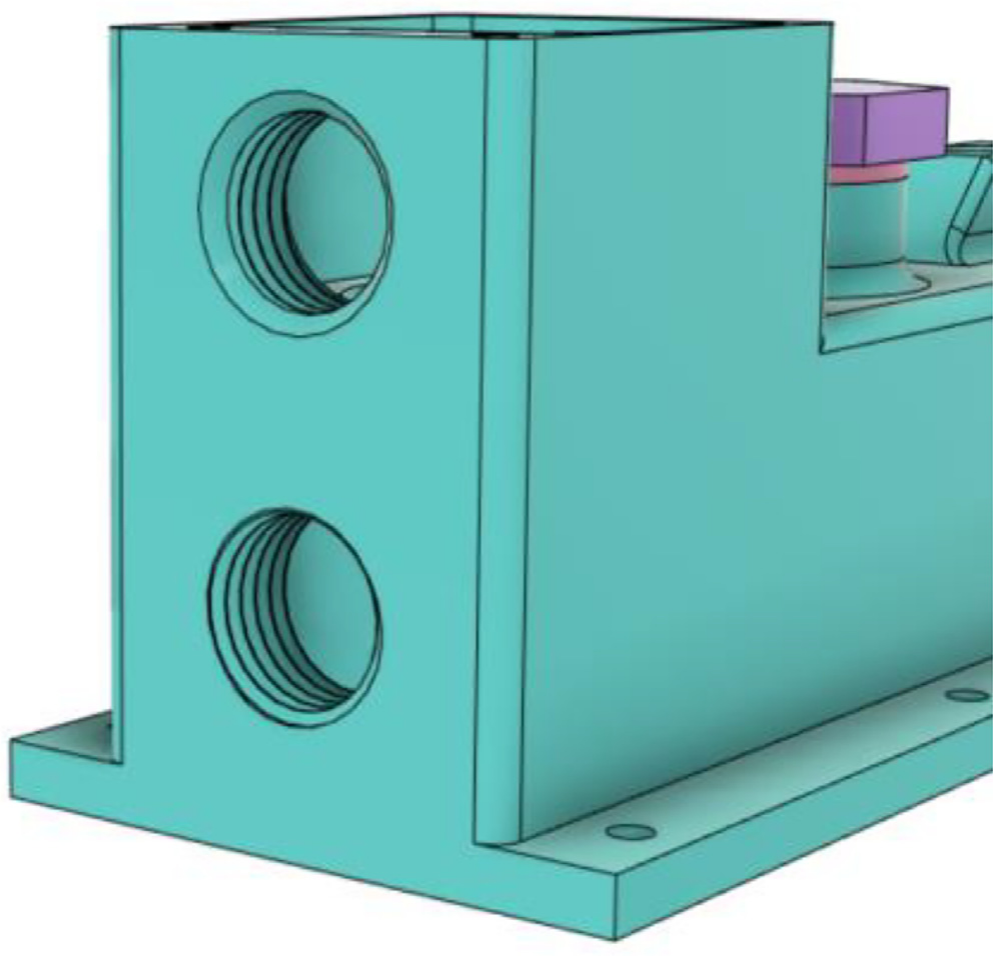
Outlet Body without the adapters.

**Fig. 79:**
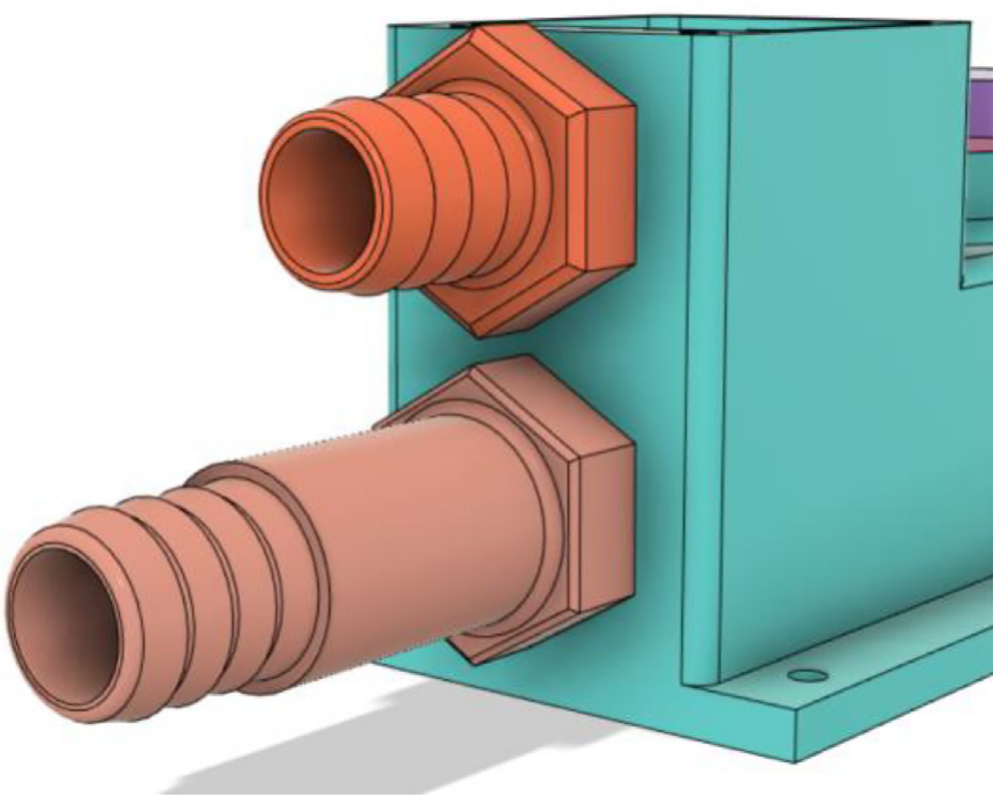
Outlet Body with the adapters.
6. Put the Exhaust Valve through the Exhaust Valve gate from the bottom of the Outlet Body and the Exhaust Gate Valve Coil Spring through the protruded screw from the top of the Outlet Body (see Fig. 80, Fig. 81)

**Fig. 80:**
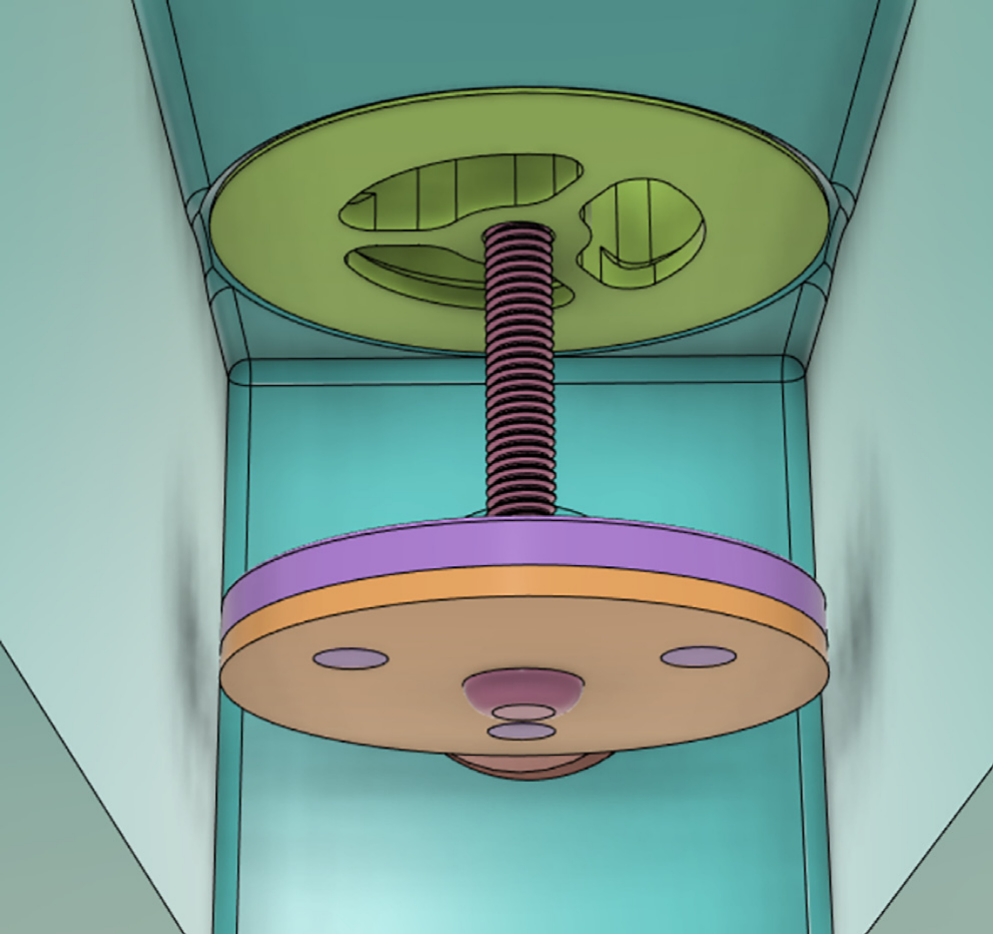
Exhaust Valve through the Exhaust Valve gate.

**Fig. 81:**
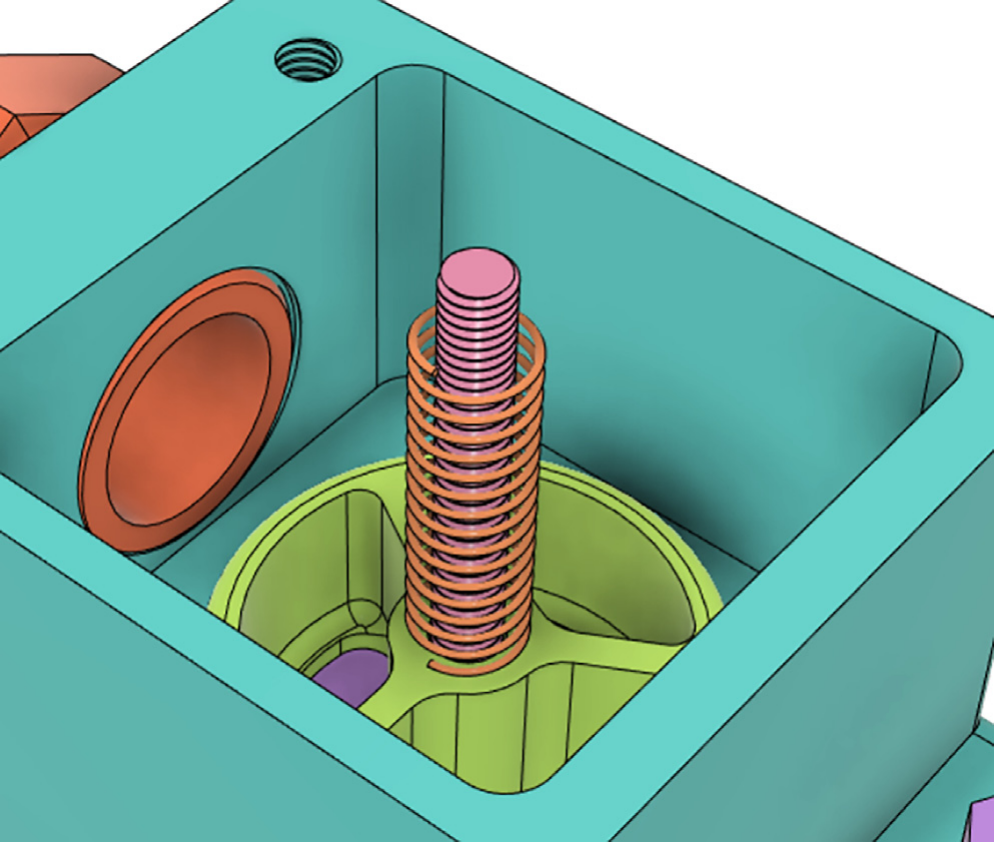
Exhaust Gate Valve Coil Spring.
7. Put the Exhaust Opening Mechanism (EOM Base and Servo bracket) on the top of the Outlet Body and then EOM (Push Body) to be screwed through the Exhaust Valve (see Fig. 82, Fig. 83, Fig. 84)

**Fig. 82:**
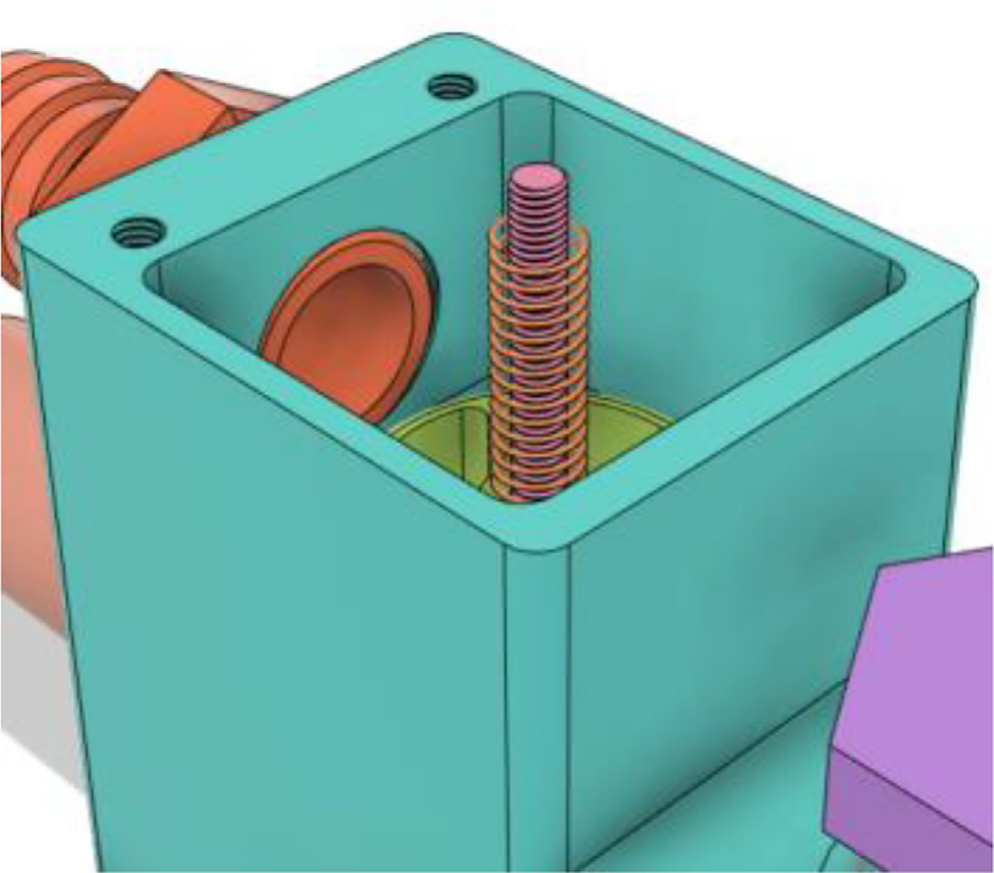
Outlet Body before EOM (Base and Servo Bracket) attached.

**Fig. 83:**
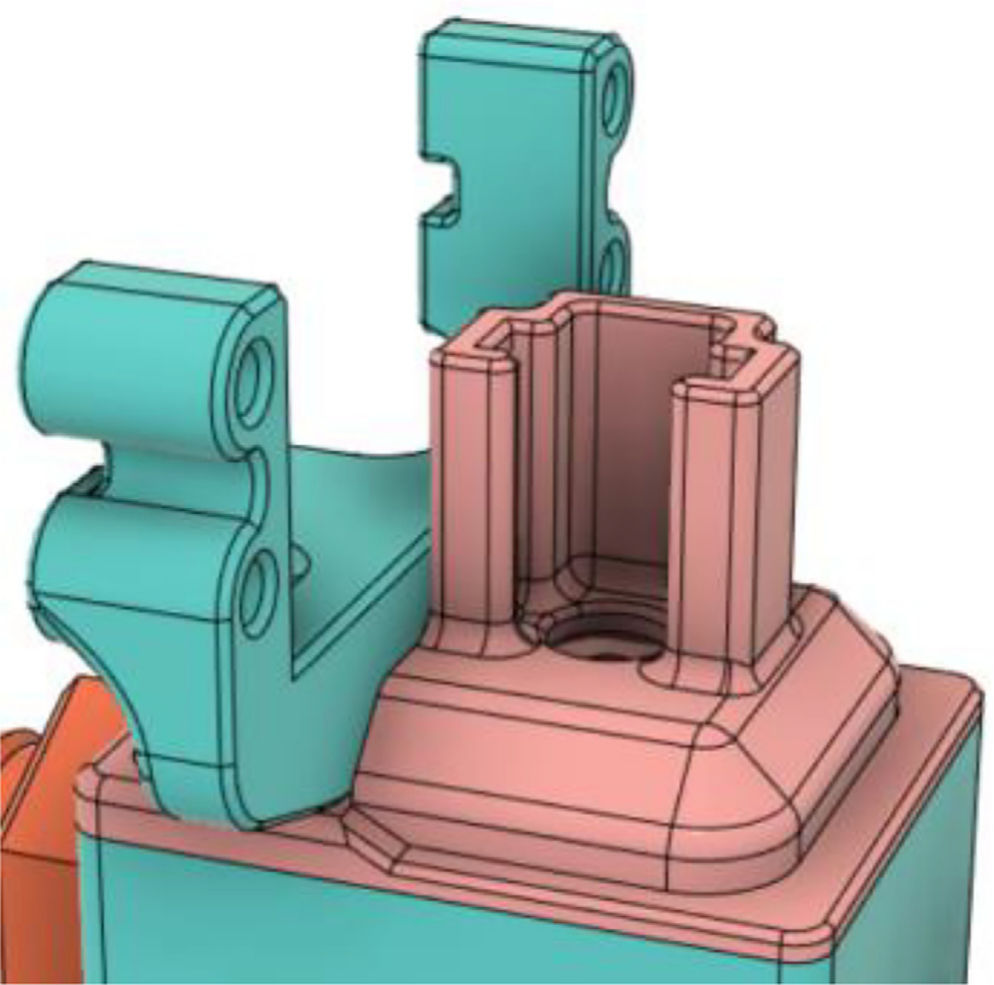
Outlet Body after EOM (Base and Servo Bracket) attached.

**Fig. 84:**
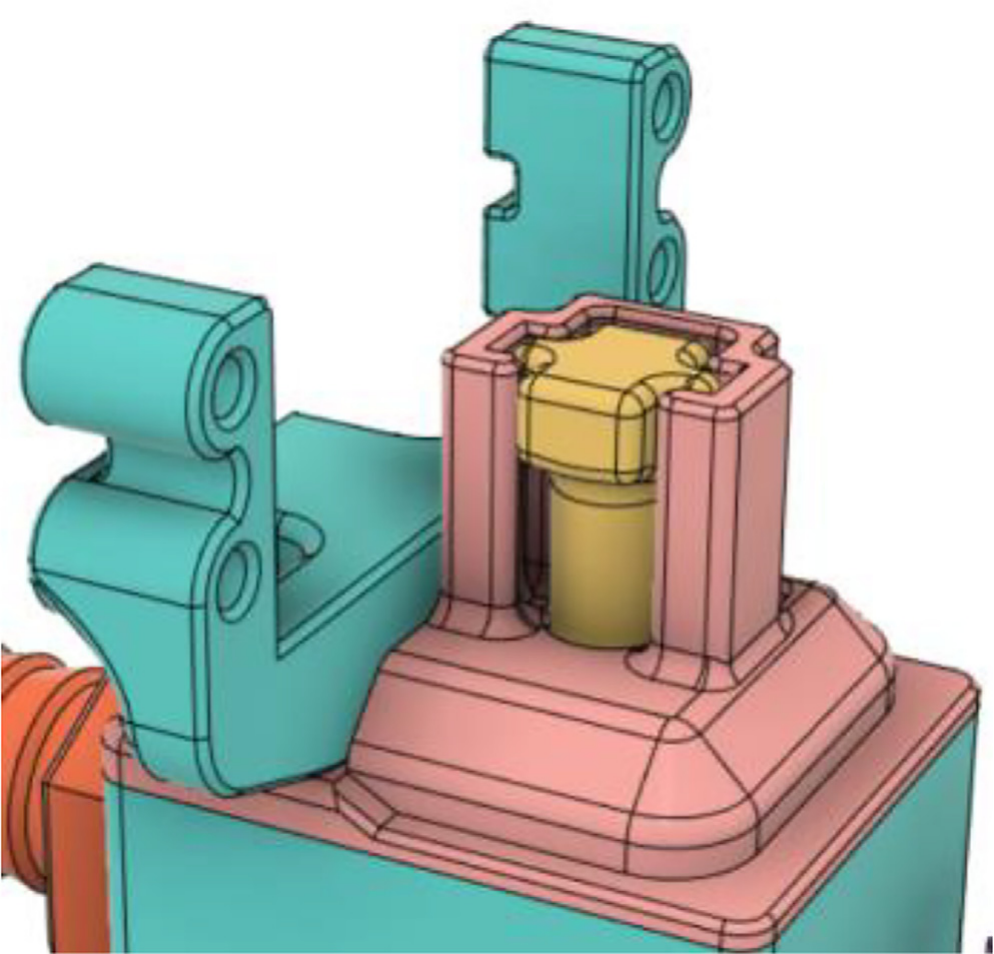
Outlet Body after EOM (Push Body) attached.
8. Screw the Servo with the EOM (Servo Bracket) and install the Servo horn with the Servo (see Fig. 85, Fig. 86)

**Fig. 85:**
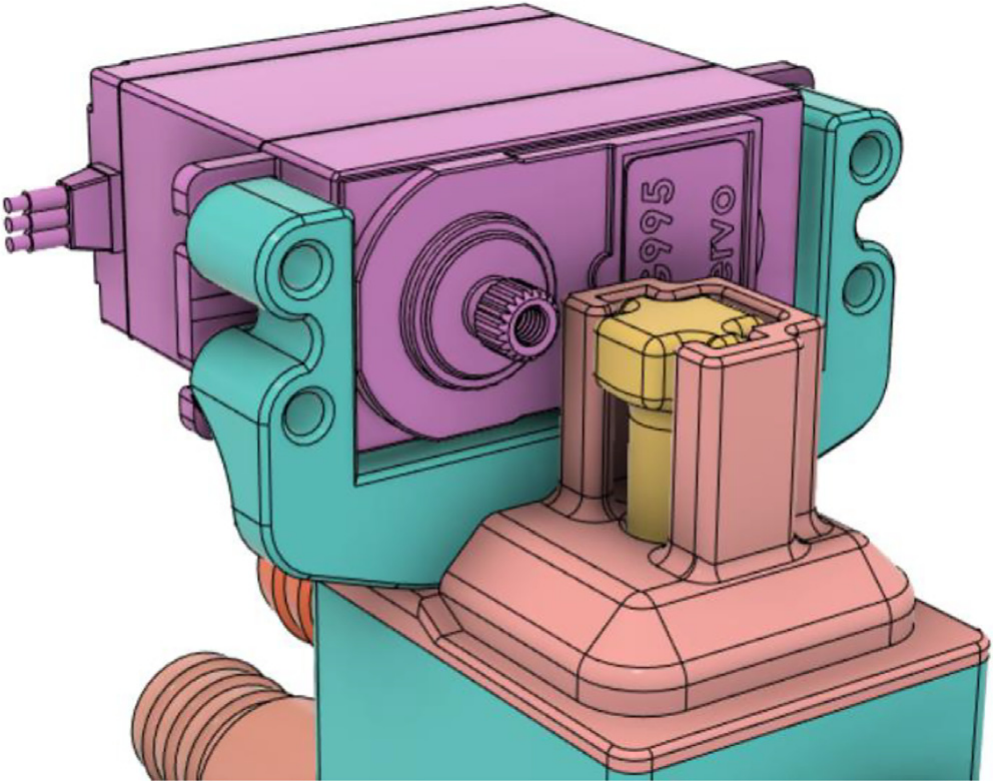
Servo installed to the EOM (Servo Bracket).

**Fig. 86:**
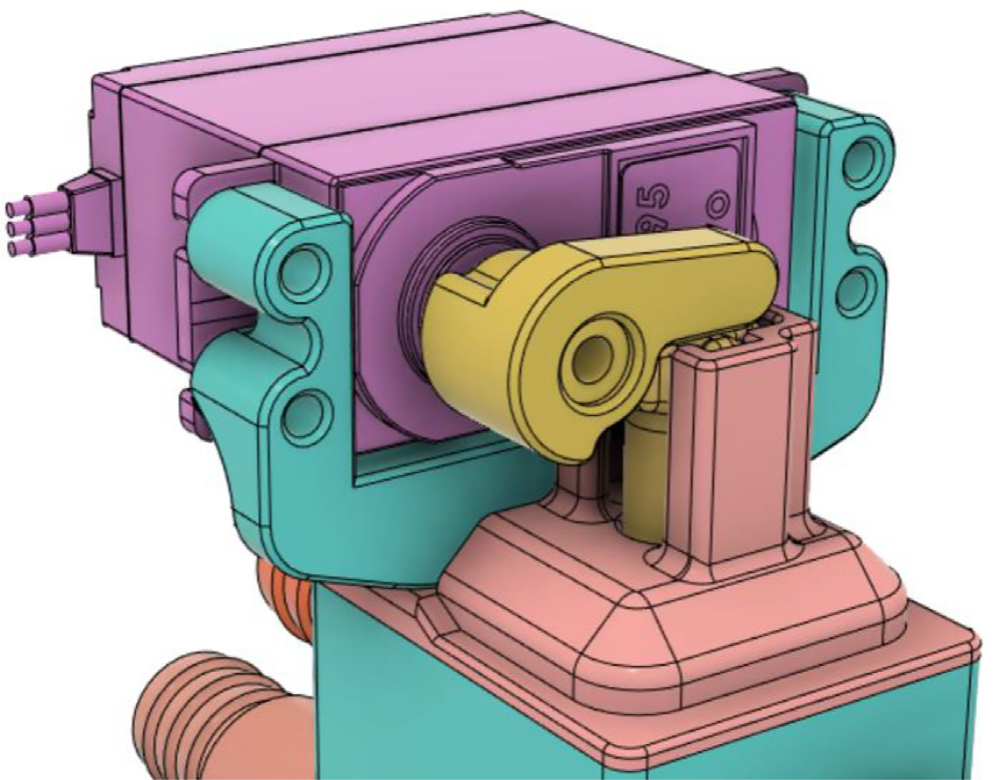
Servo Horn installed to the Servo.
9. Glue the bottom base of the Outlet Body Base with the Outlet Body using Silicone Glue (see Fig. 87, Fig. 88).

**Fig. 87:**
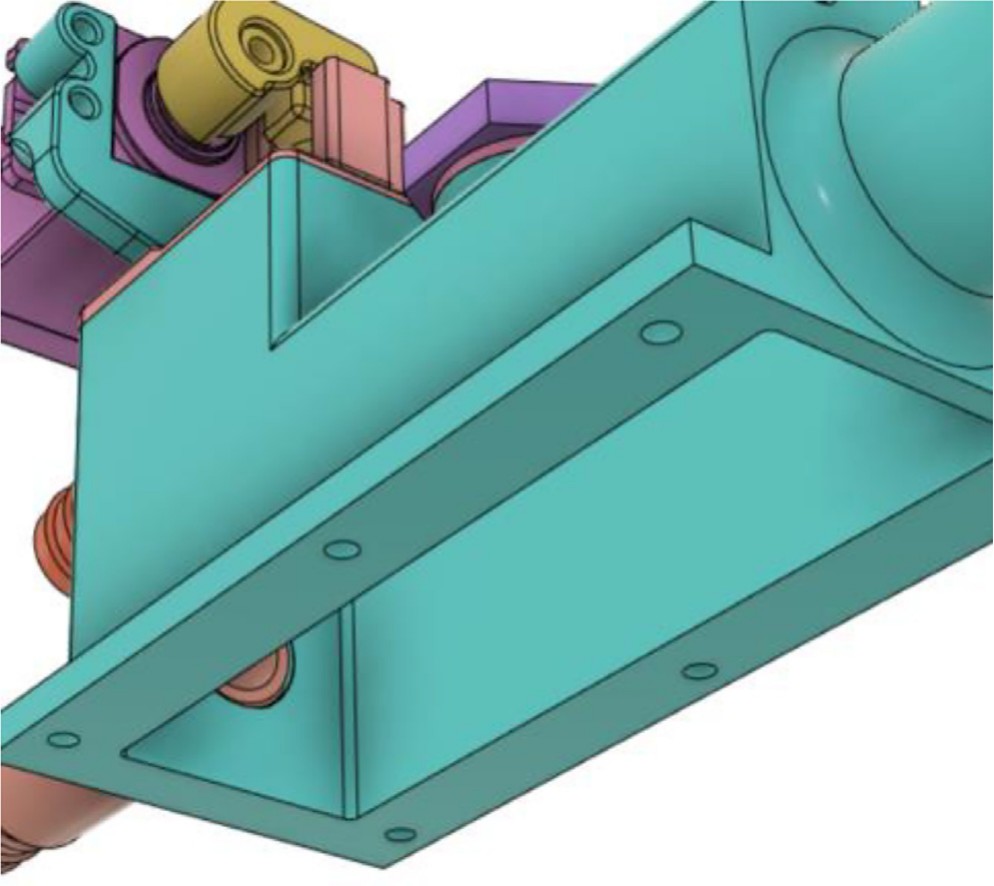
Without Outlet Base.

**Fig. 88:**
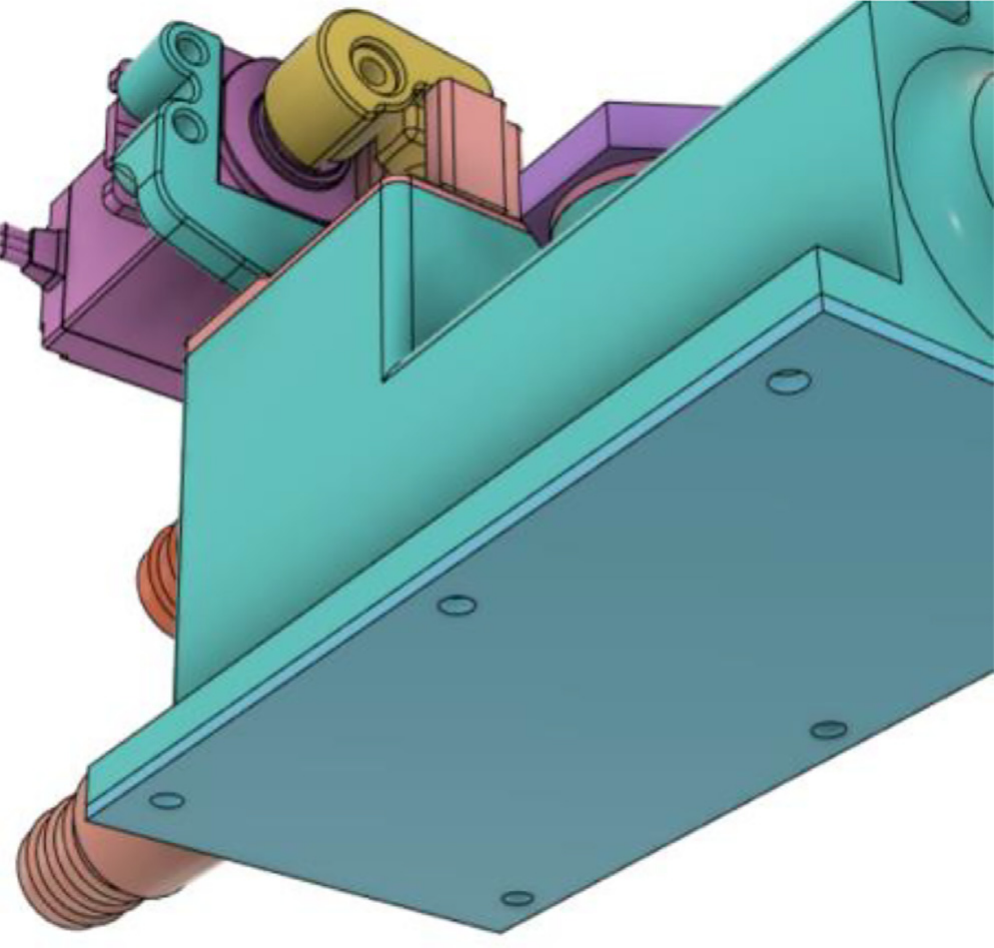
With Outlet Base.

### Filter and Pump Assembly

**Fig. 89:**
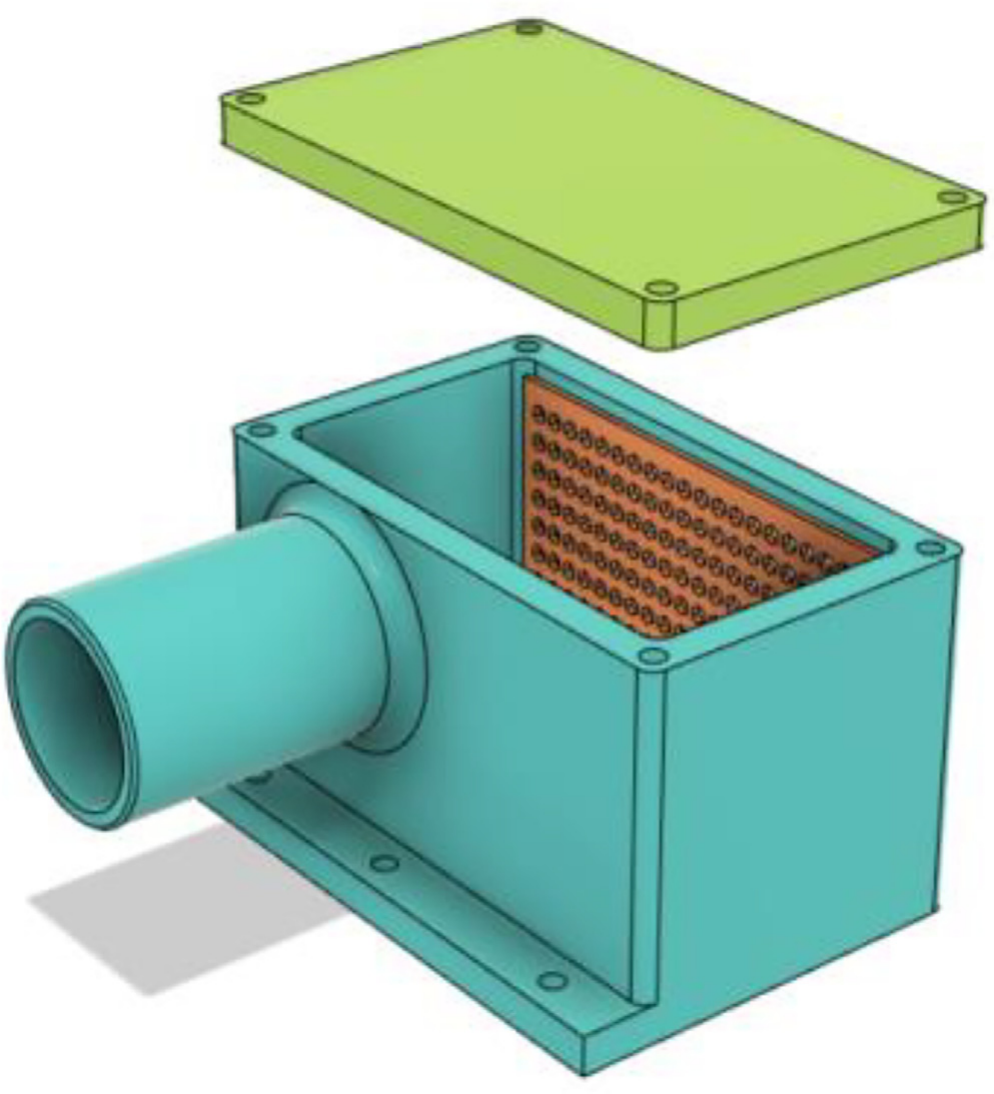
HME Cover open.

1. Install the HME Inlet Mesh and put the HEPA filter inside. Then screw in the HME Cover onto place (see Fig. 89, Fig. 90)

**Fig. 90:**
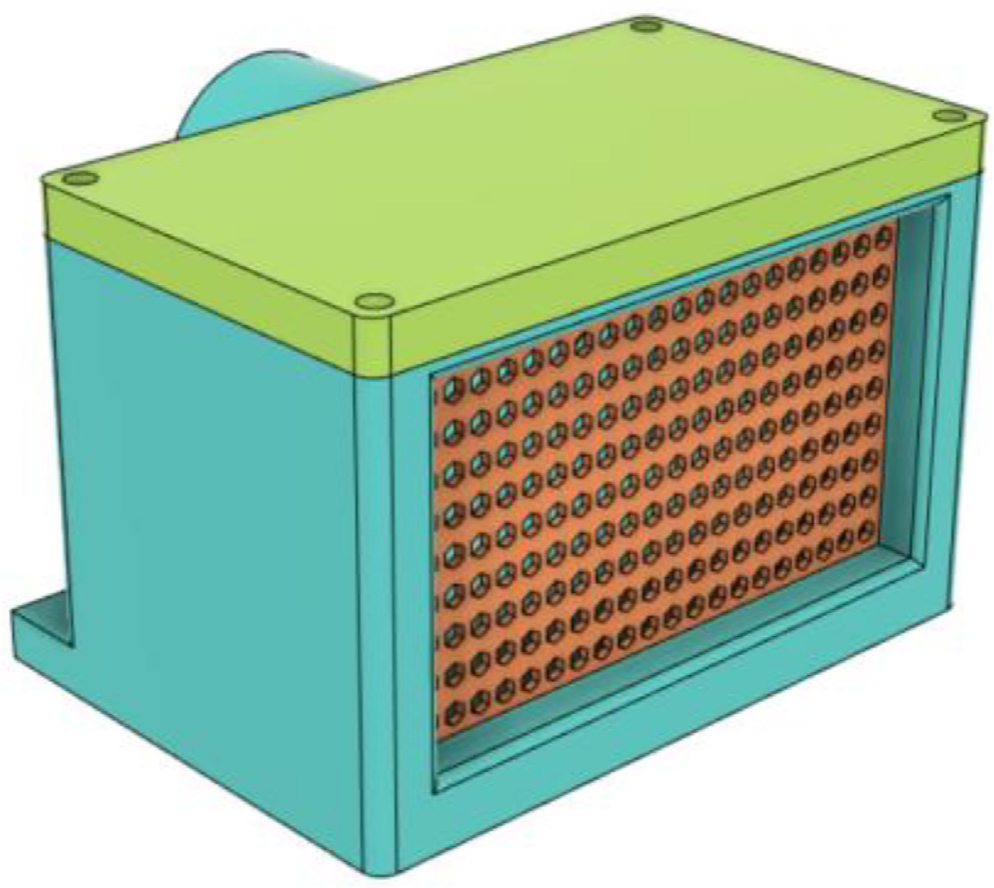
HME Filter assembled.
2. Screw down the HME Filter with the Casing Base 12 (see Fig. 91, Fig. 92)

**Fig. 91:**
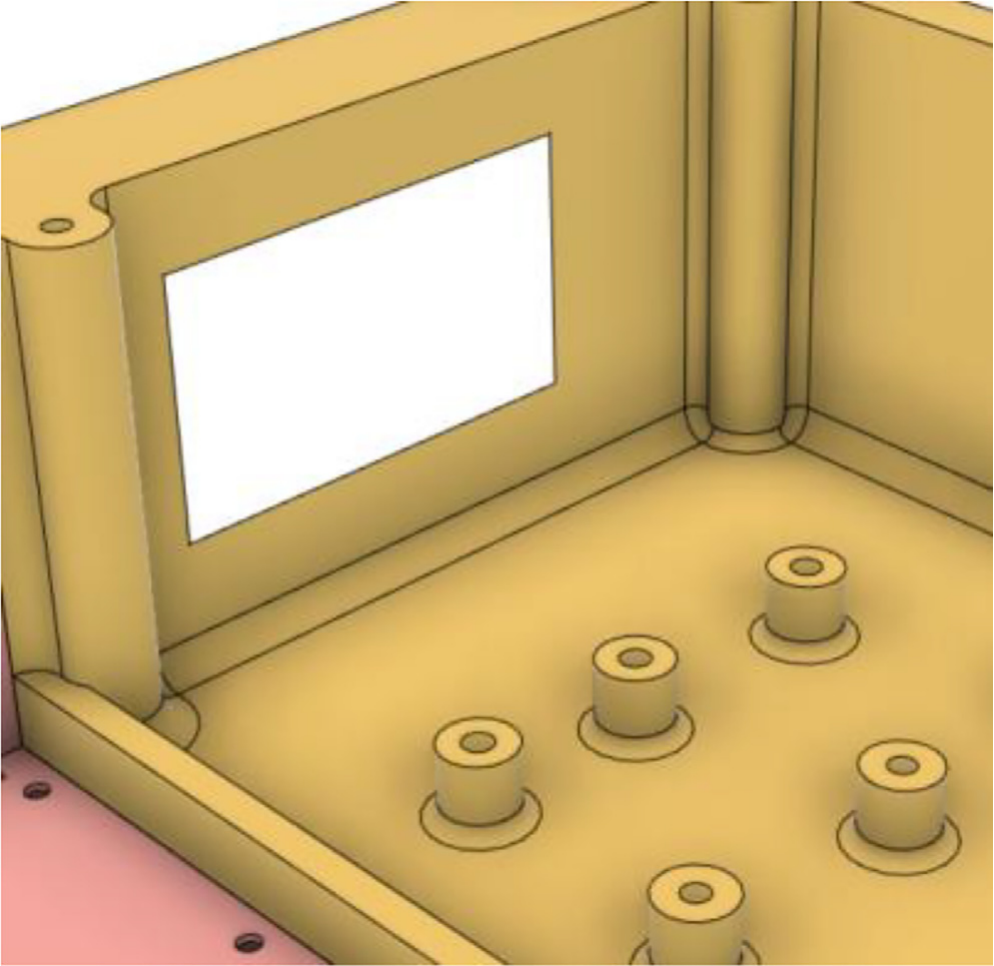
Before screwing down the HME Filter with the Base 12.

**Fig. 92:**
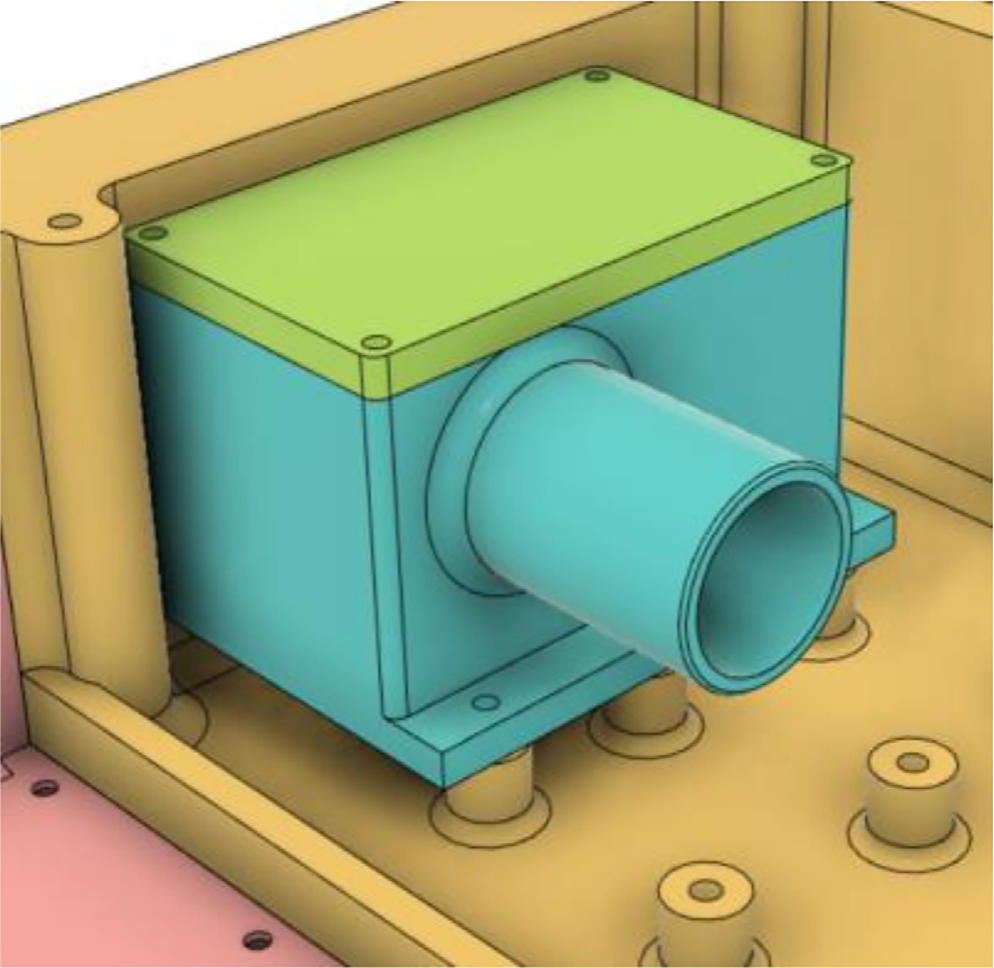
Before screwing down the HME Filter with the Base 12.
3. Install the Proportional Valve Body with the Inlet Body. Screw the bodies together from below (see Fig. 93, Fig. 94)

**Fig. 93:**
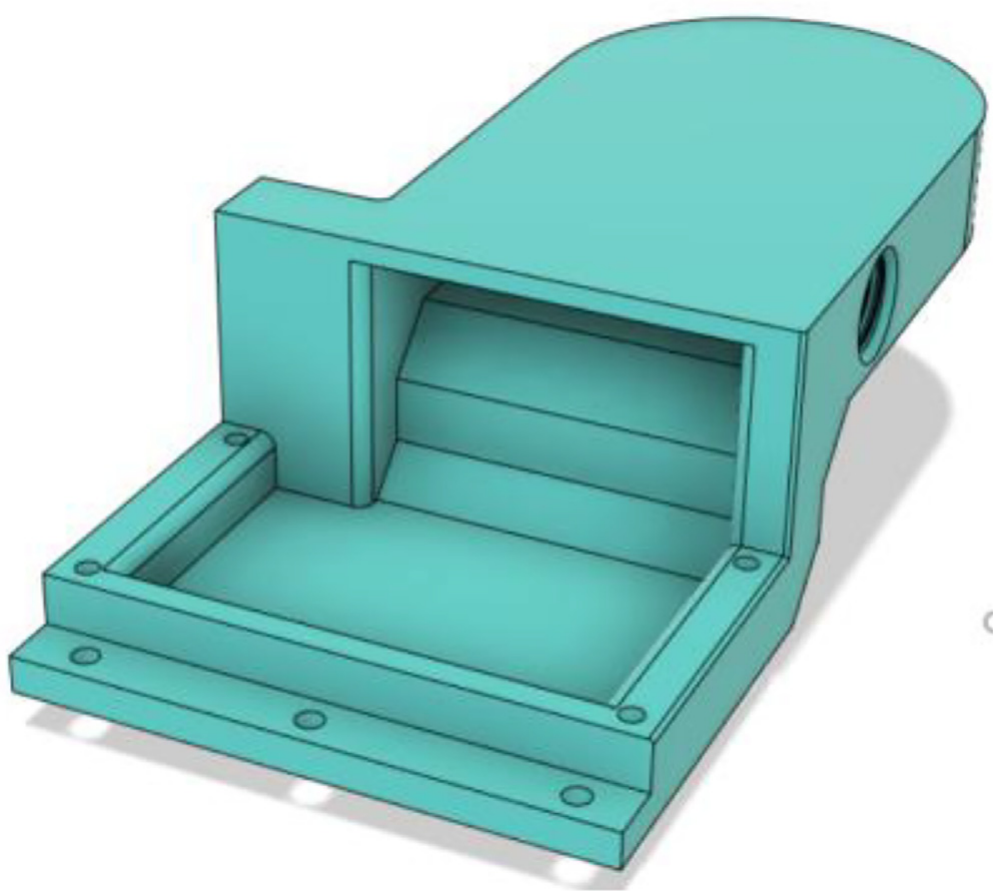
Before screwing down the Proportional Valve Body.

**Fig. 94:**
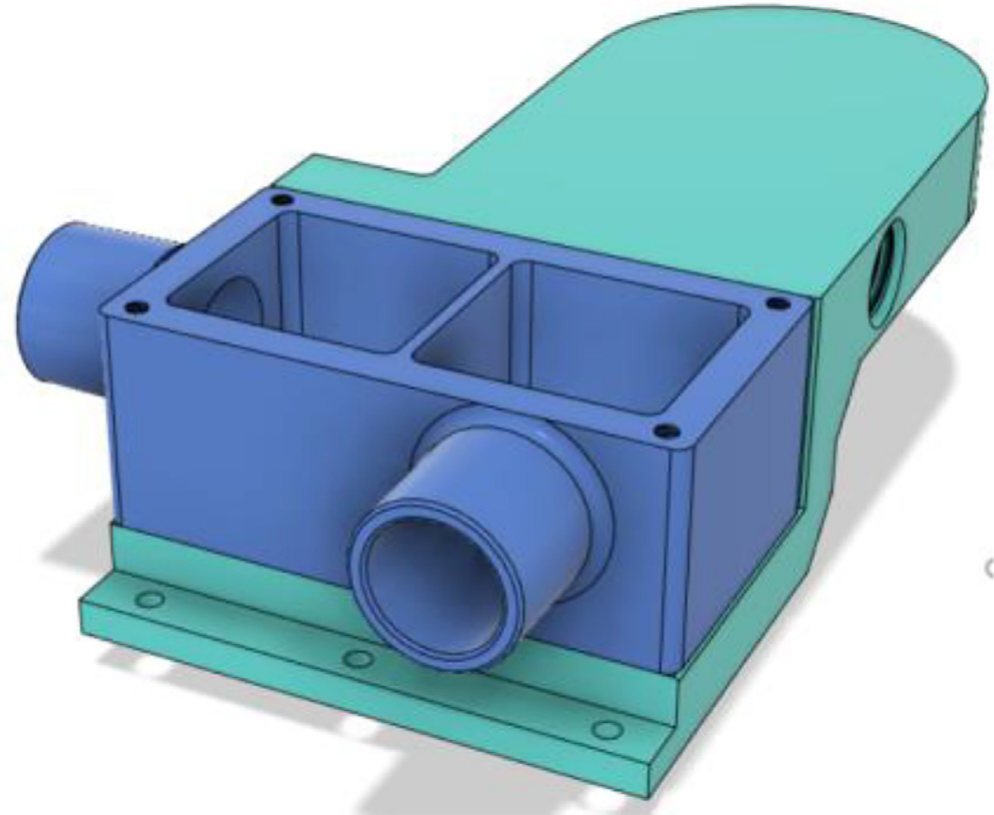
After screwing down the Proportional Valve Body.
4. Screw the 9 g Micro Servos on the top of the Proportional Valve Cover and then screw the Proportional Valve Cover with the Proportional Valve Body (see Fig. 95, Fig. 96)

**Fig. 95:**
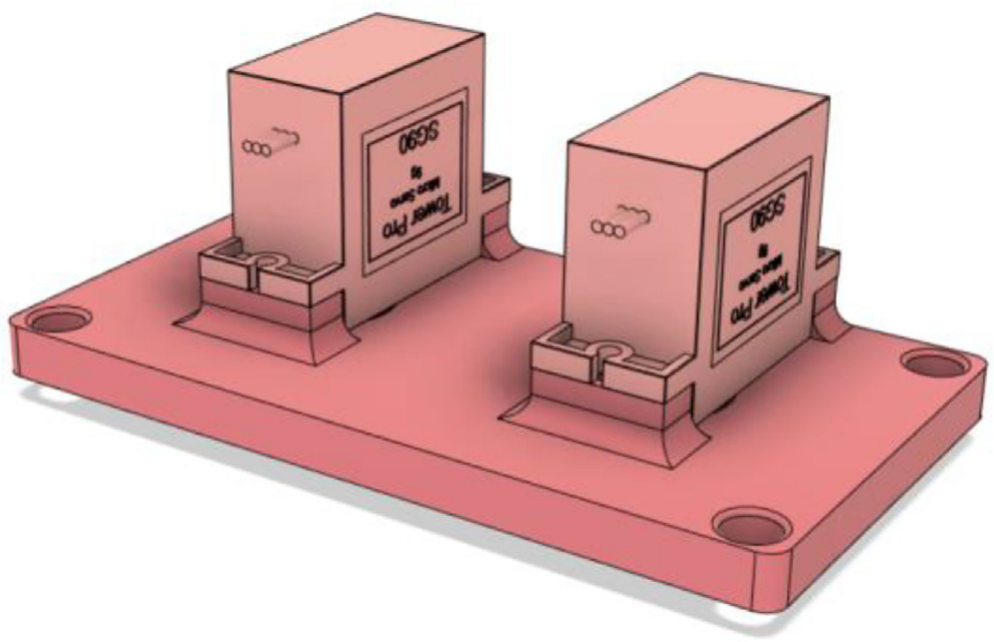
Screw down the Micro Tower 9 g servos on the Proportional Valve Cover.

**Fig. 96:**
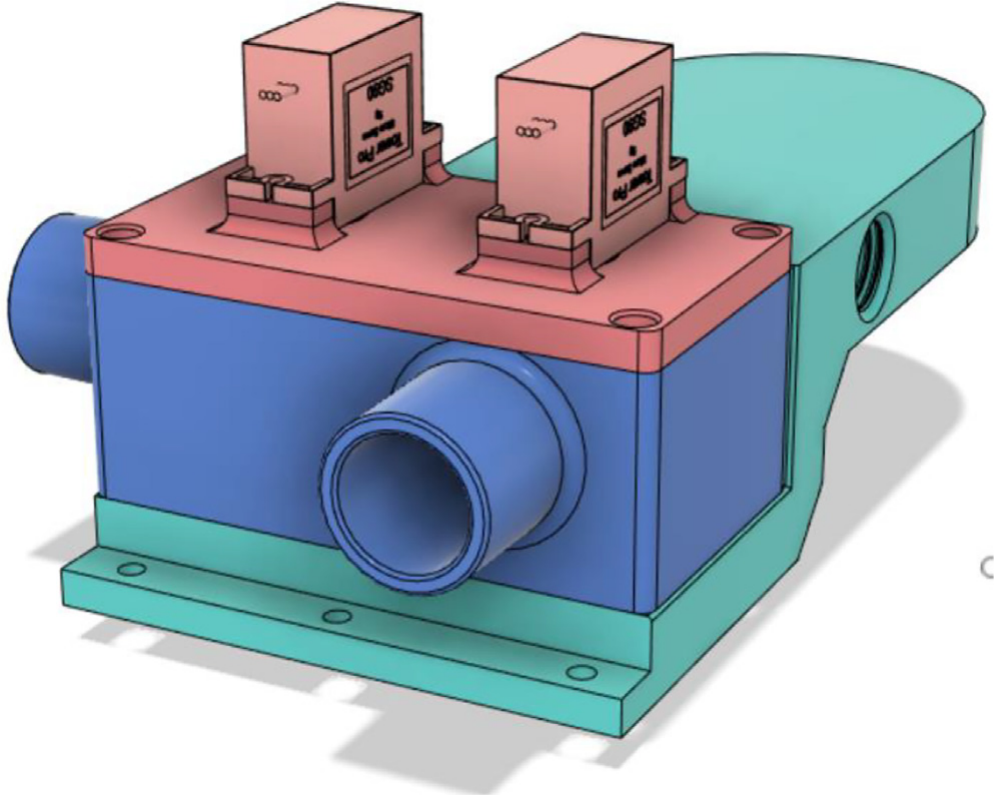
Screw down the Proportional Valve Cover onto the Proportional Valve Body.
5. Press-fit the WM7040 Pump to the Inlet Body (see Fig. 97, Fig. 98)

**Fig. 97:**
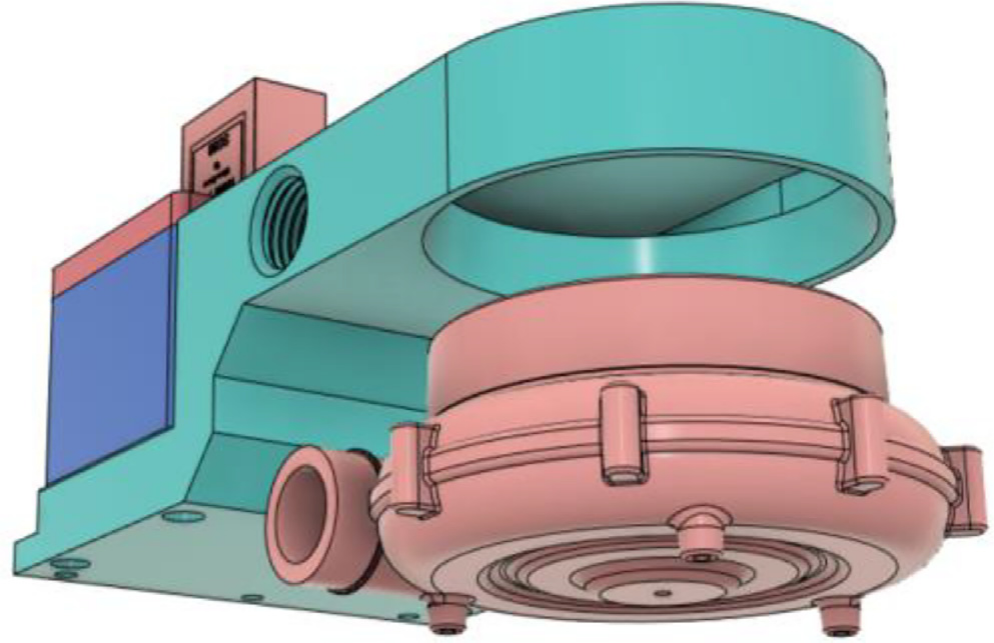
Before press-fitting the WM7040 Pump to the Inlet Body.

**Fig. 98:**
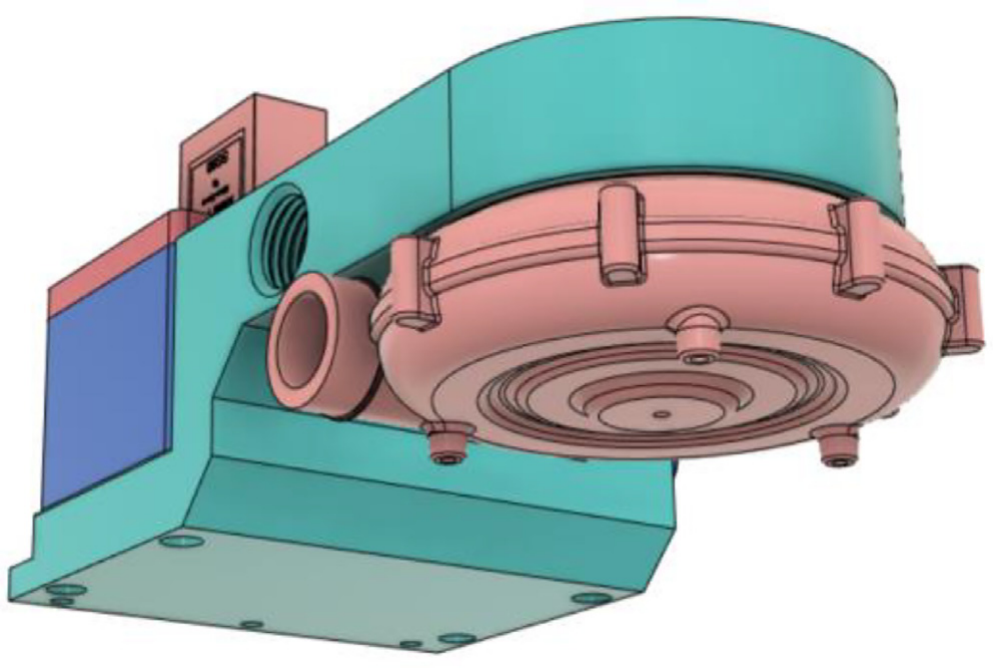
After press-fitting the WM7040 Pump to the Inlet Body.
6. Screw in the 14 mm Adapter (Short) with the Inlet Body and press-fit the 14 mm Adapter (Pump outlet) with the Pump (see Fig. 99, Fig. 100)

**Fig. 99:**
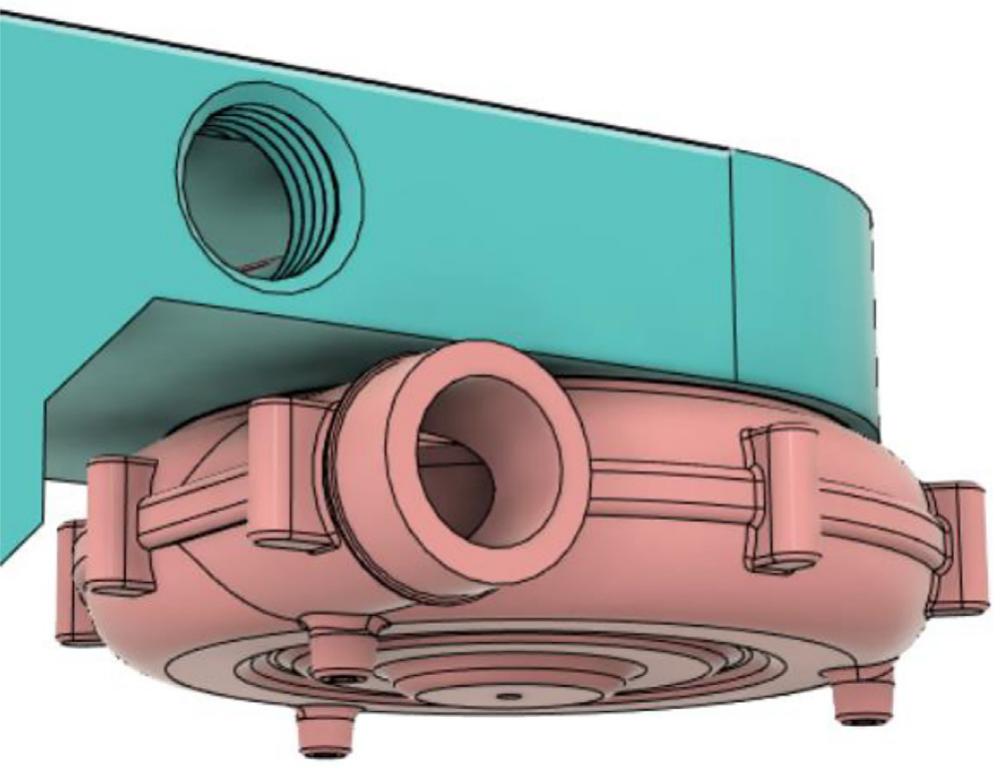
Before installing the 14 mm adapters.

**Fig. 100:**
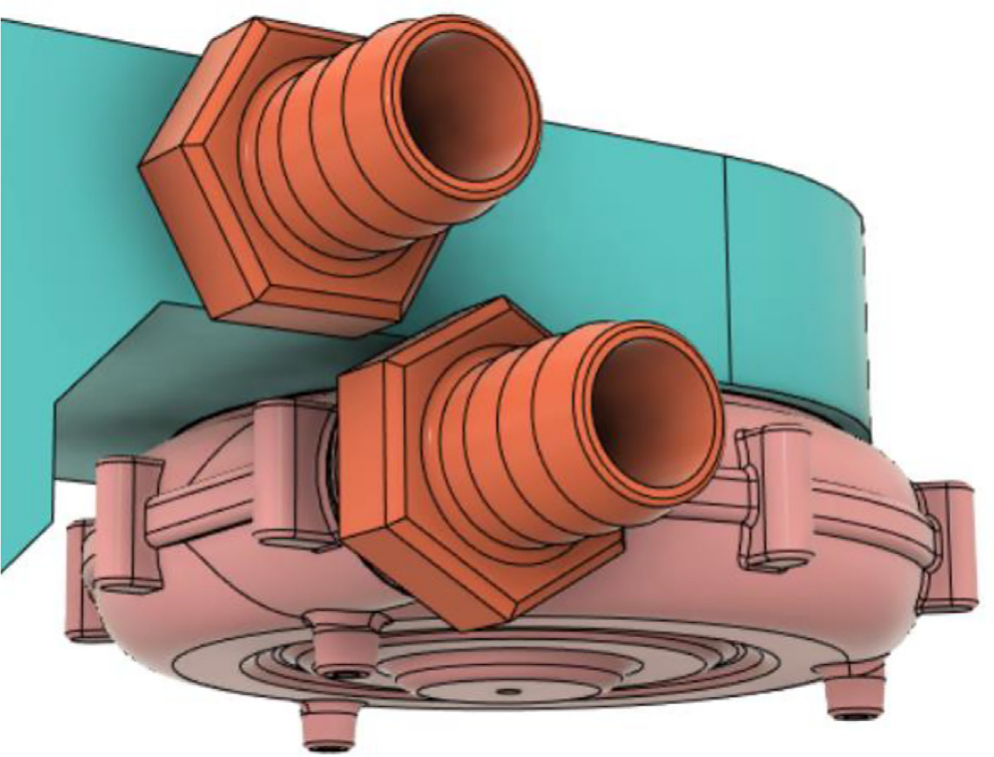
After installing the 14 mm adapters.
7. Slide the Inlet Body and Pump assembly with the HME Filter outlet (see Fig. 101, Fig. 102)

**Fig. 101:**
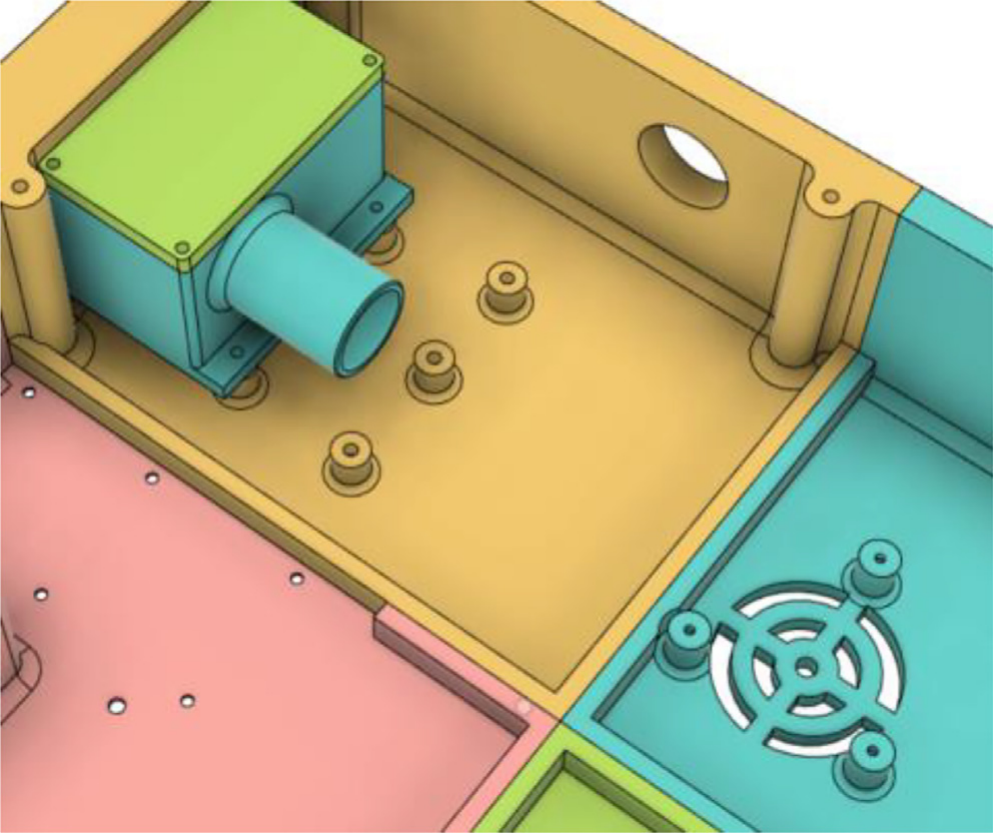
Before sliding in the Inlet Body and Pump assembly.

**Fig. 102:**
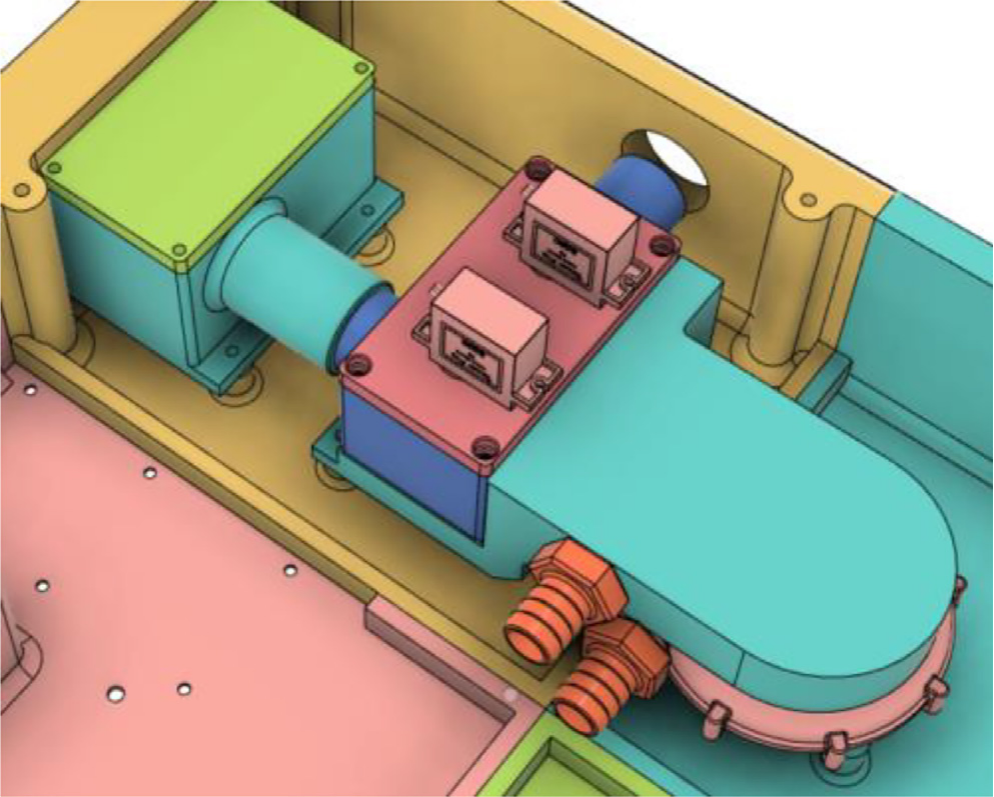
After sliding in the Inlet Body and Pump assembly.
8. Screw the 6 screws for installing the Inlet Body and Pump assembly with the Casing Base
9. Screw the Oxy Nozzle Body with the Oxy Nozzle Holder (see Fig. 103, Fig. 104)

**Fig. 103:**
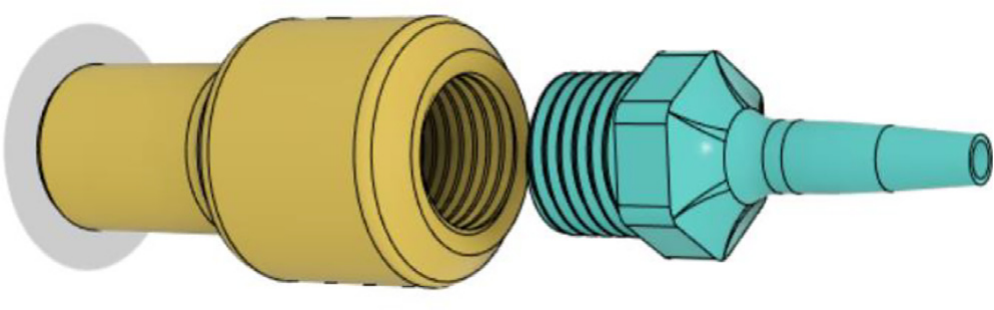
Before screwing the Oxy Nozzle Body with the Oxy Nozzle Holder.

**Fig. 104:**
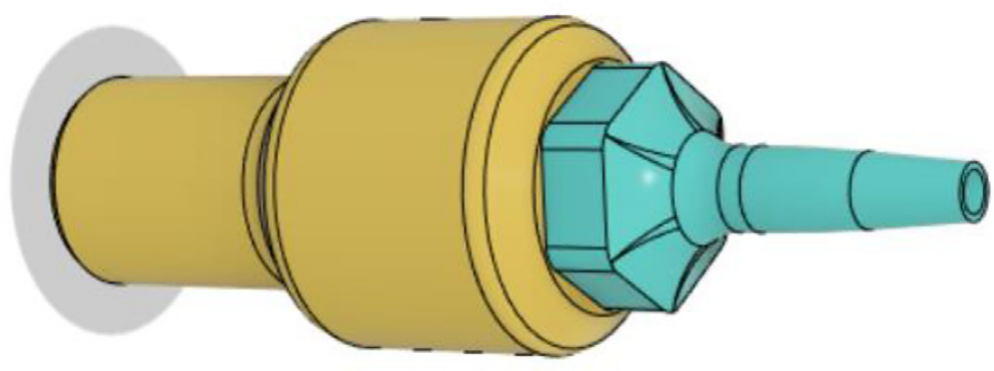
After screwing the Oxy Nozzle Body with the Oxy Nozzle Holder.
10. Press-fit the Oxy Nozzle with the Inlet Body (see Fig. 105, Fig. 106)

**Fig. 105:**
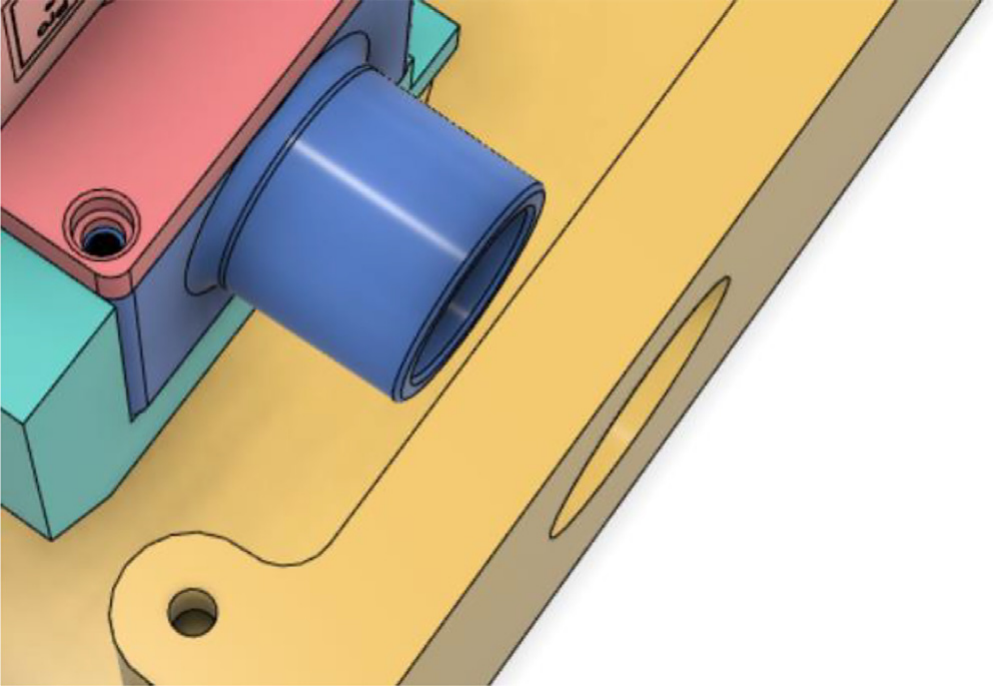
Before press-fitting the Oxy Nozzle Body with the Oxy Nozzle Holder.

**Fig. 106:**
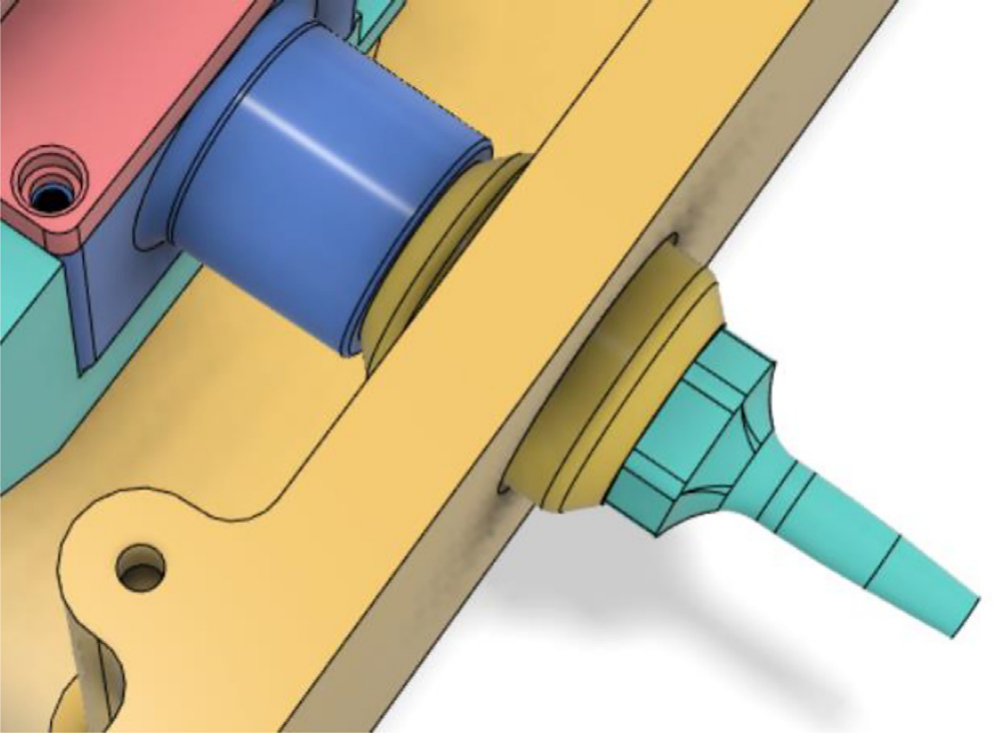
After press-fitting the Oxy Nozzle Body with the Oxy Nozzle Holder.

### Final assembly

**Fig. 107:**
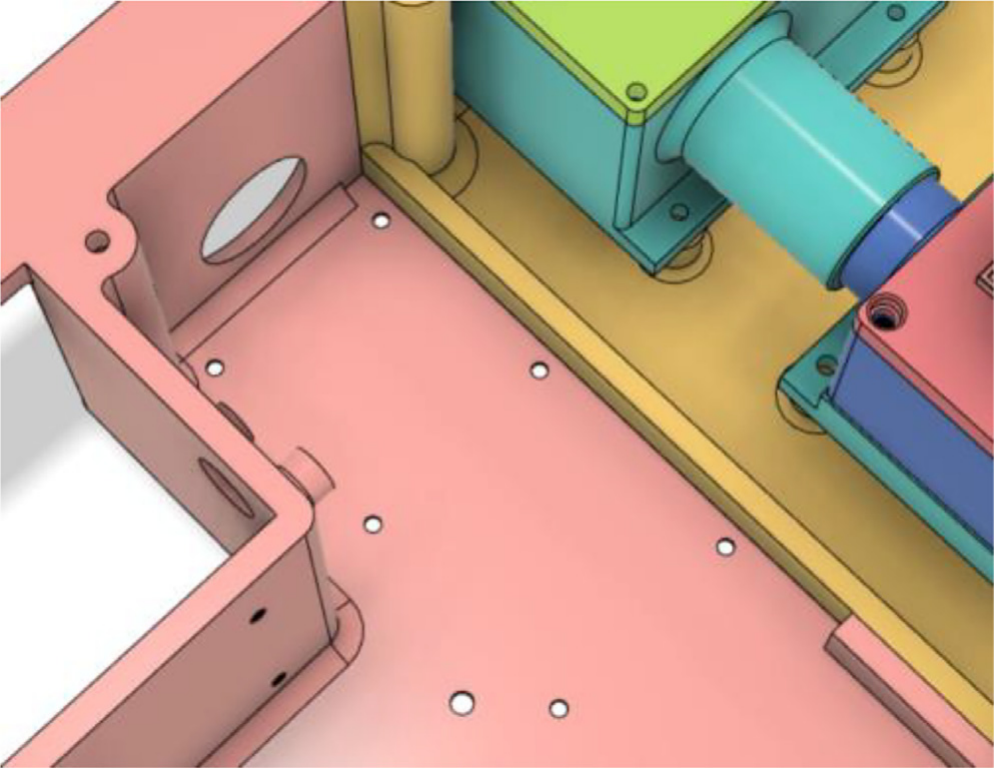
Before screwing down the Pressure Release Mechanism (PRM) with the Casing Base 11.

1. Screw down the Pressure Release Mechanism (PRM) with the Casing Base 11 (see Fig. 107, Fig. 108)

**Fig. 108:**
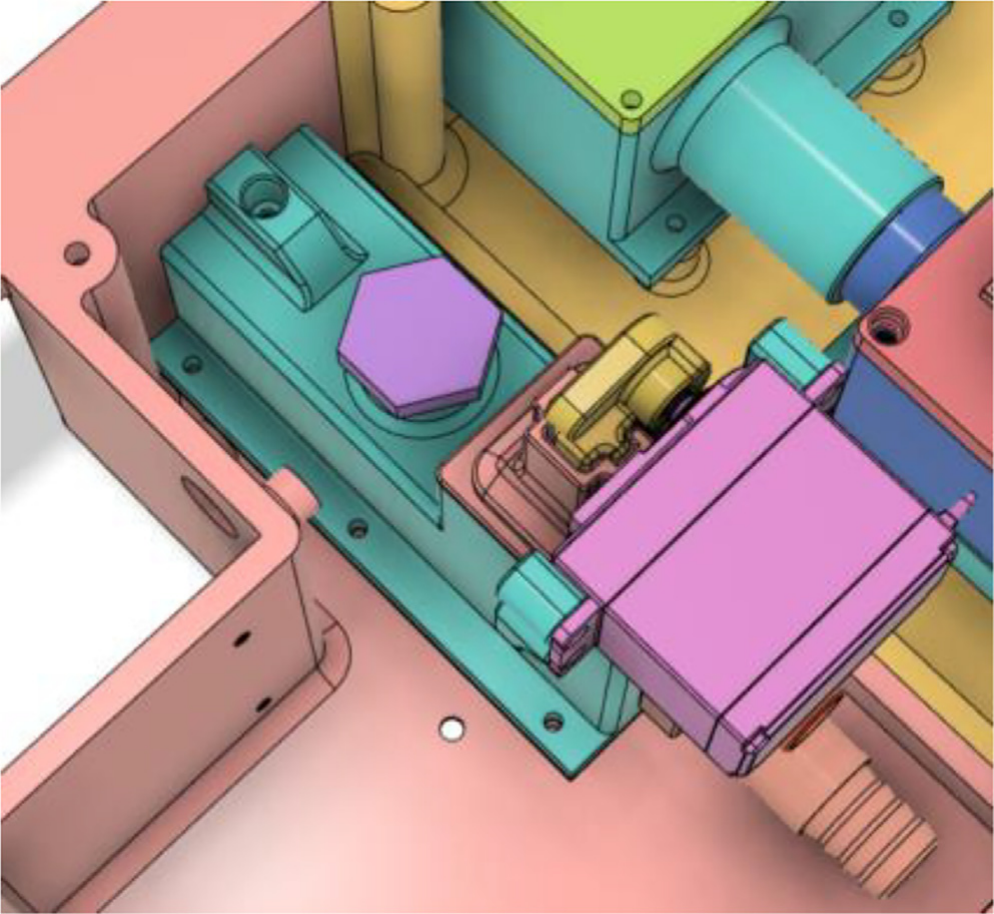
After screwing down the Pressure Release Mechanism (PRM) with the Casing Base 11.
2. Attach the One-way Valve to the outlet of the PRM (see Fig. 109, Fig. 110)

**Fig. 109:**
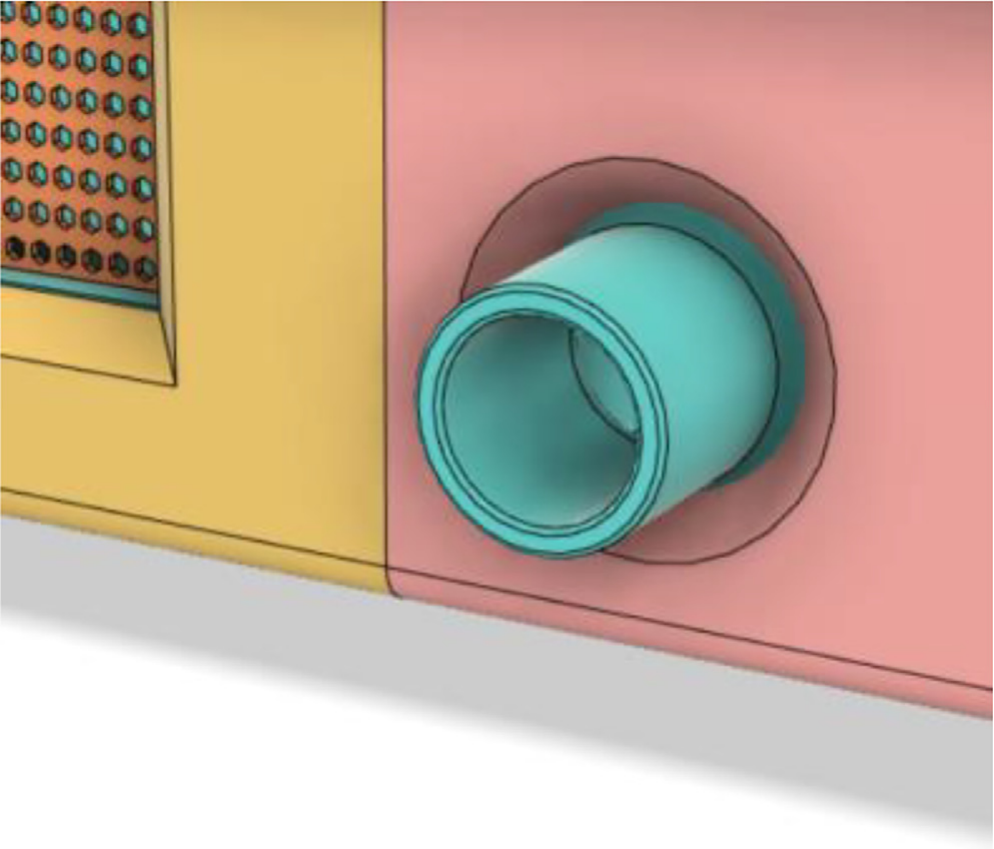
Before attaching the One-way Valve to the outlet of the PRM.

**Fig. 110:**
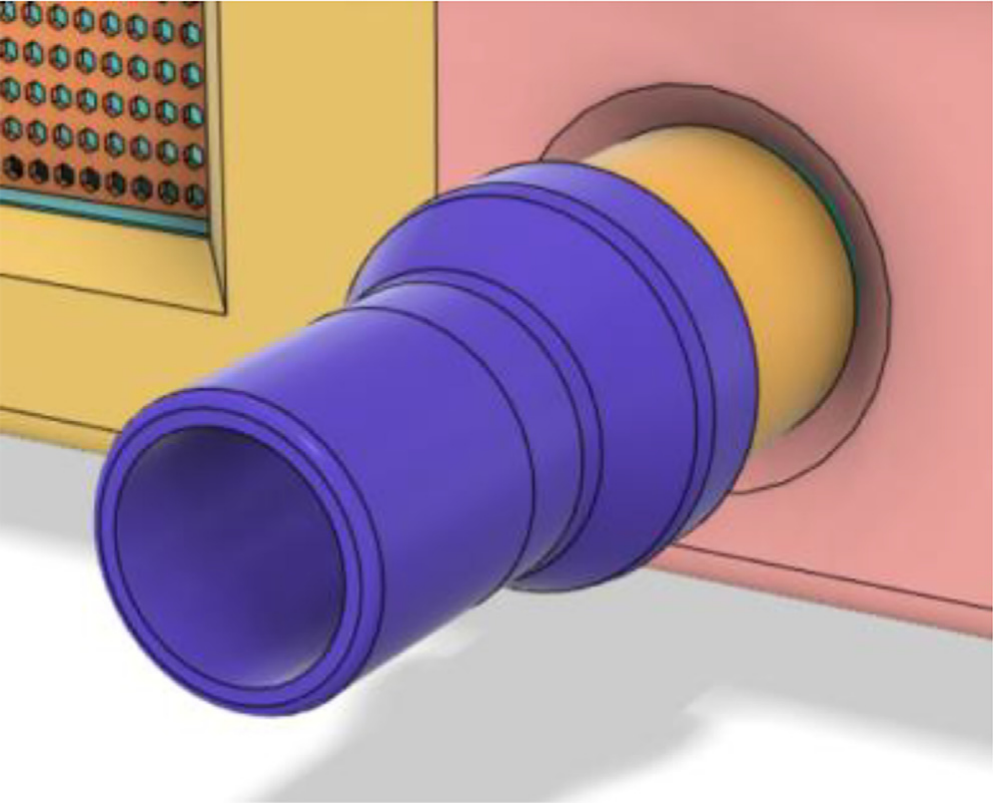
After attaching the One-way Valve to the outlet of the PRM.
3. Install Silicone Tube (Hose Release) with the Adapter 14 mm (short) of the Inlet Body to the Adapter 14 mm (short) of the PRM and install Silicone Tube (Hose Oxy) with the Adapter 14 mm (Pump Outlet) of the Pump to the Adapter 14 mm (Long) of the PRM (see Fig. 111, Fig. 112)

**Fig. 111:**
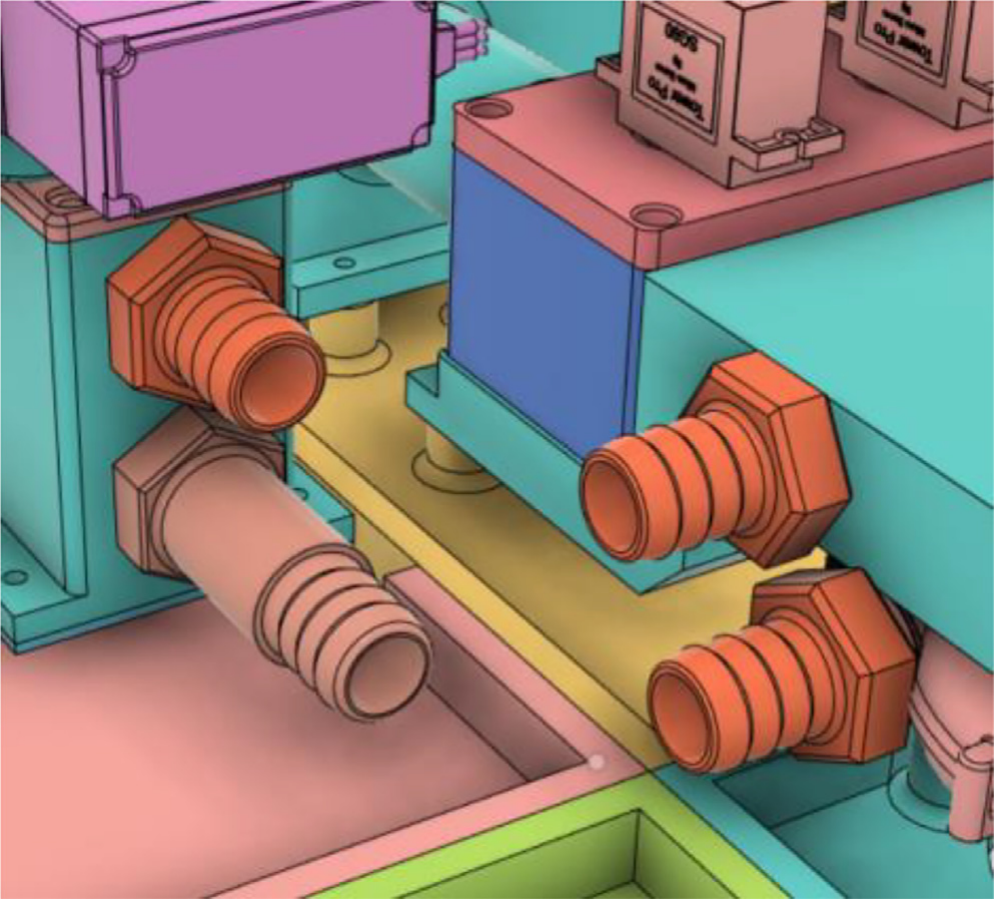
Before attaching the silicone tubes.

**Fig. 112:**
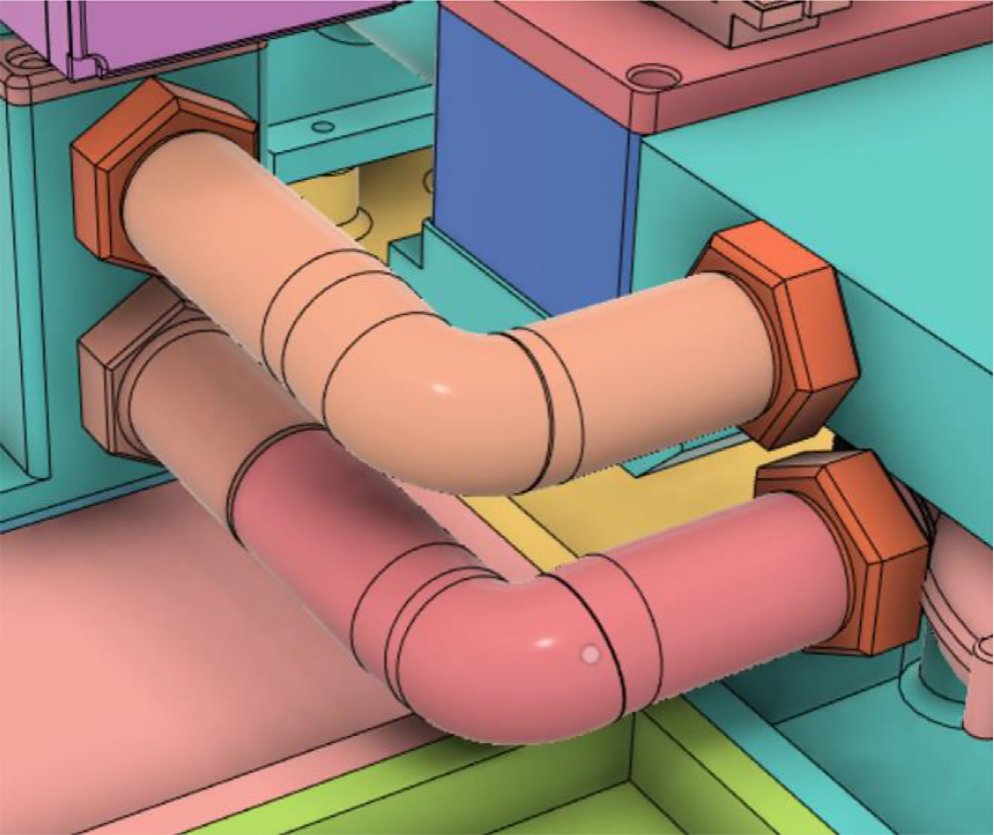
After attaching the silicone tubes.
4. Attach the PEEP Hose with PRM's outlet body and put it through Casing Base 11 for it to be attached with the PEEP Valve a bit later (see Fig. 113, Fig. 114)

**Fig. 113:**
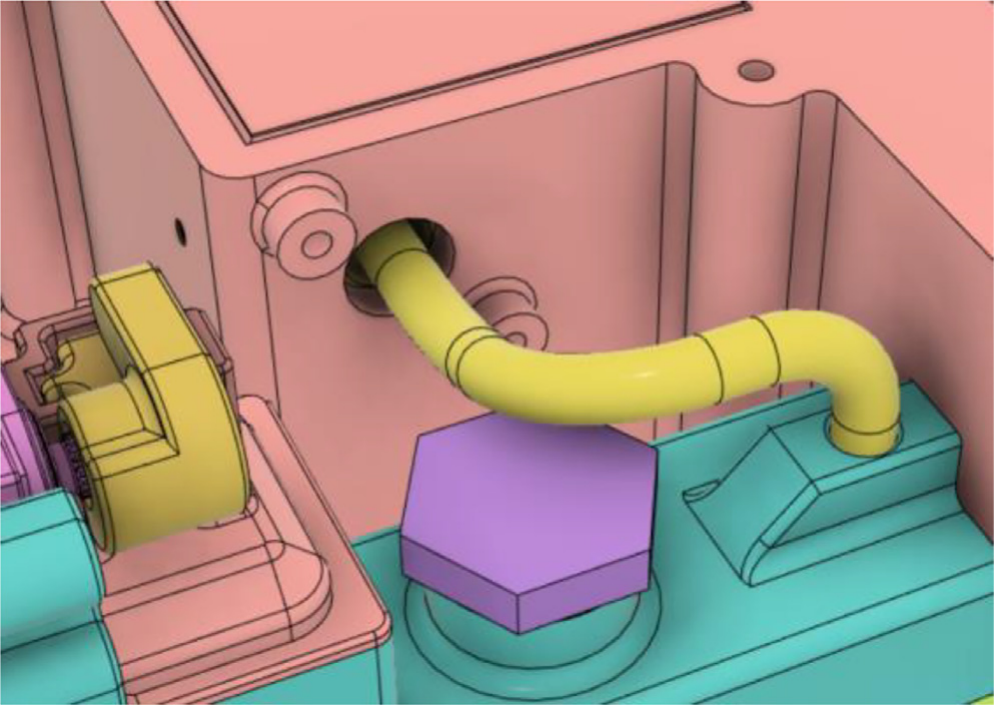
The PEEP Hose with PRM's Outlet Body and putting it through the Casing Base 11.

**Fig. 114:**
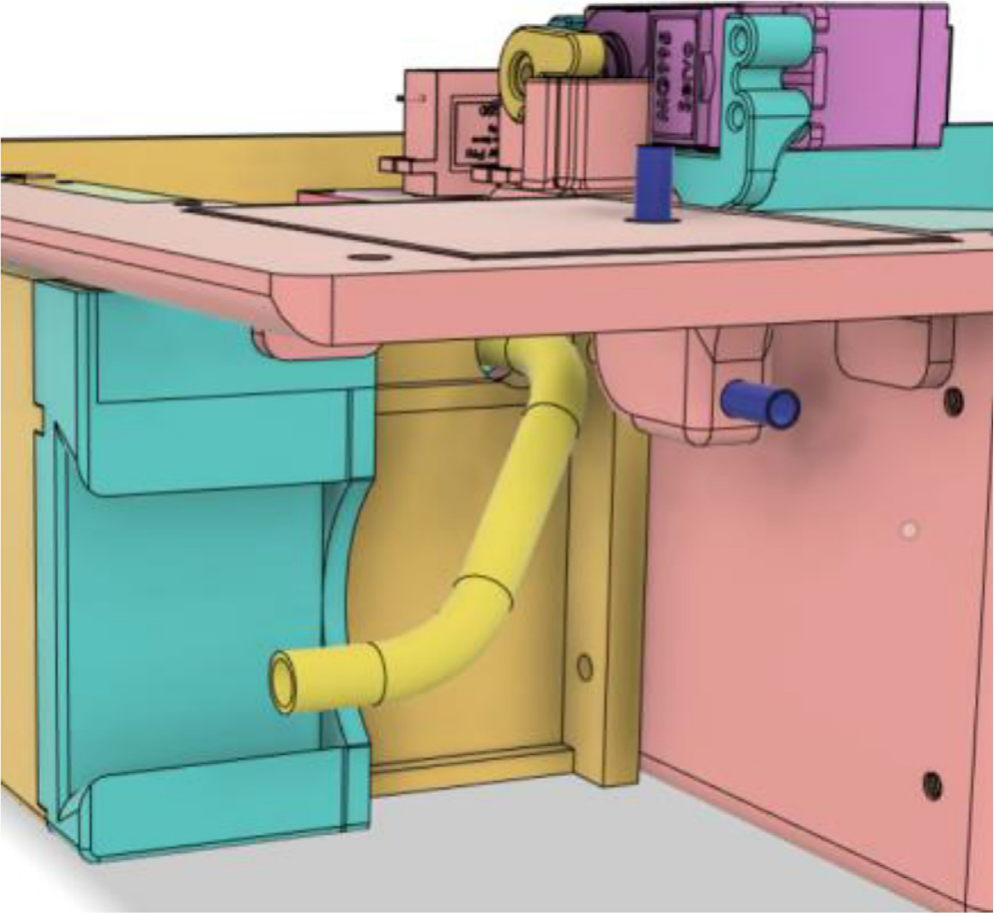
The PEEP Hose through the Front Extension 1 and ready to connect to PEEP Valve later on.
5. Slide in the PEEP Valve through the Front Extension 1 and attach the PEEP Hose with it Screw the PSU with the PSU Mount and slide into the PSU Holder Tabs to finally screw down the PSU Mount with Casing Base (22 and 11) (see Fig. 115, Fig. 116, Fig. 117, Fig. 118)

**Fig. 115:**
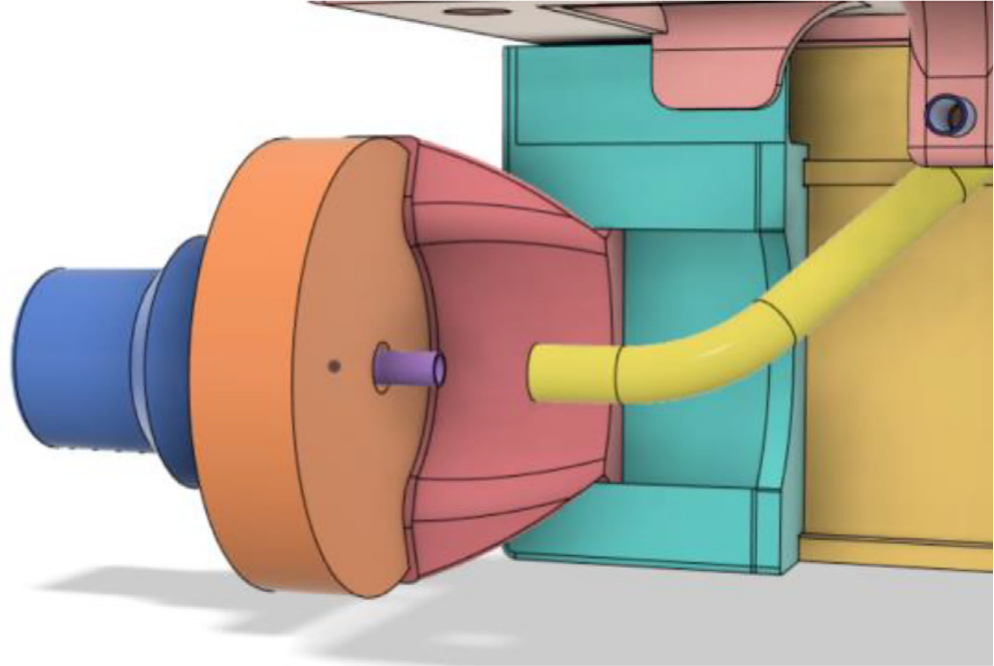
Sliding in the PEEP Valve through the Front Extension 1.

**Fig. 116:**
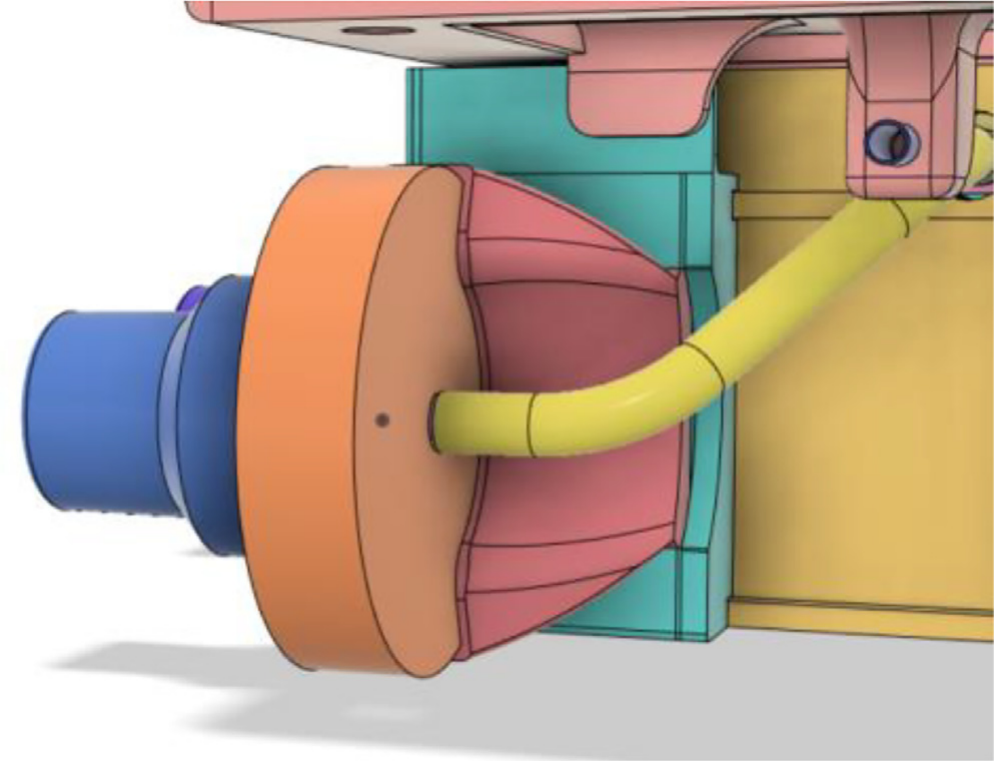
Attach the PEEP Hose with the PEEP Valve.

**Fig. 117:**
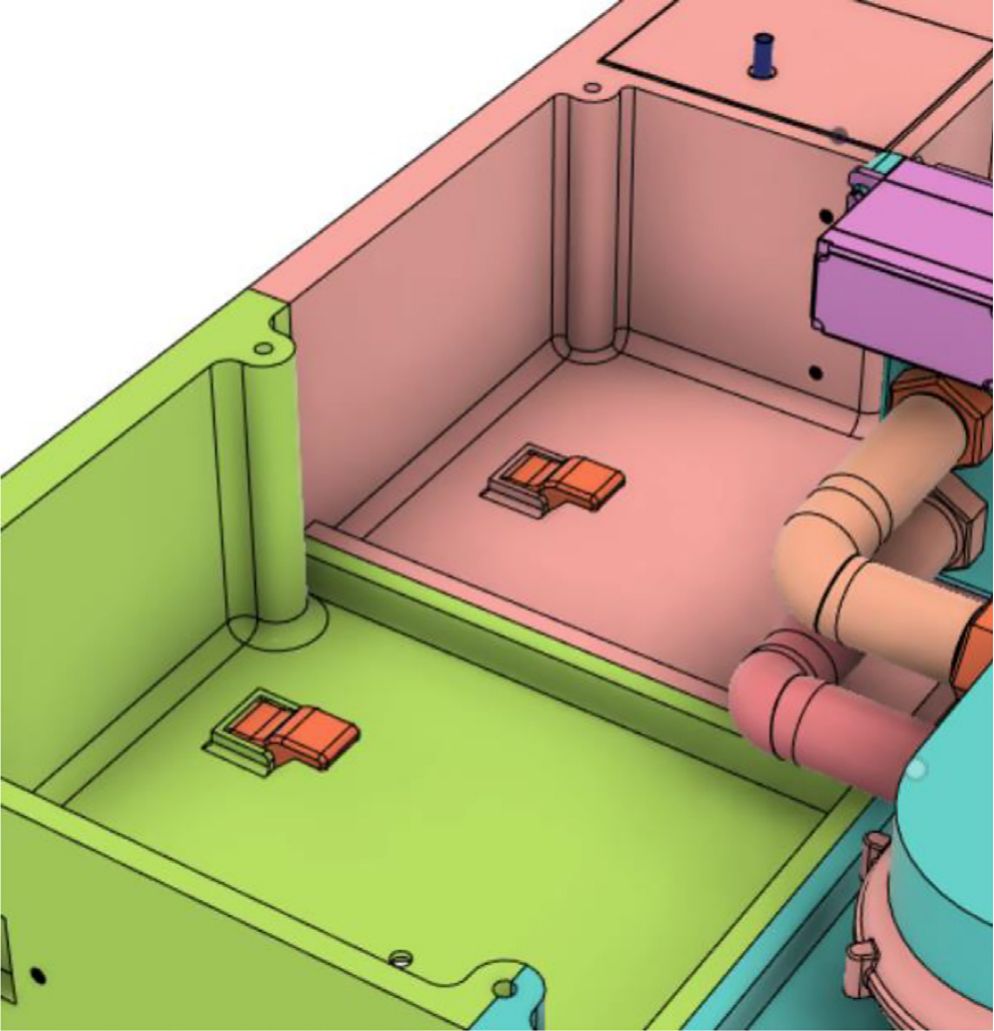
Before installing the PSU Mount and the SMPS.

**Fig. 118:**
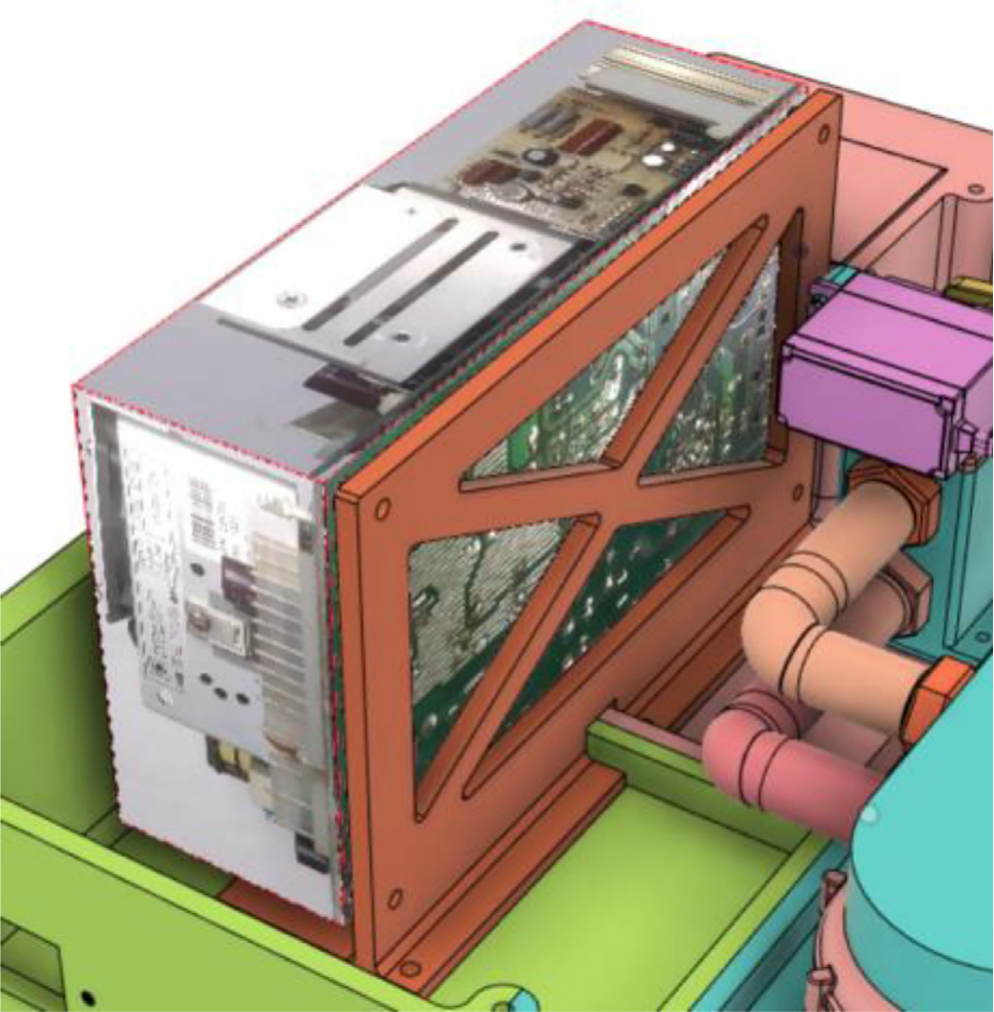
After installing the PSU Mount and the SMPS.
6. Glue the Battery Case (Holder) with the Casing Base (22) (see Fig. 119, Fig. 120, Fig. 121)

**Fig. 119:**
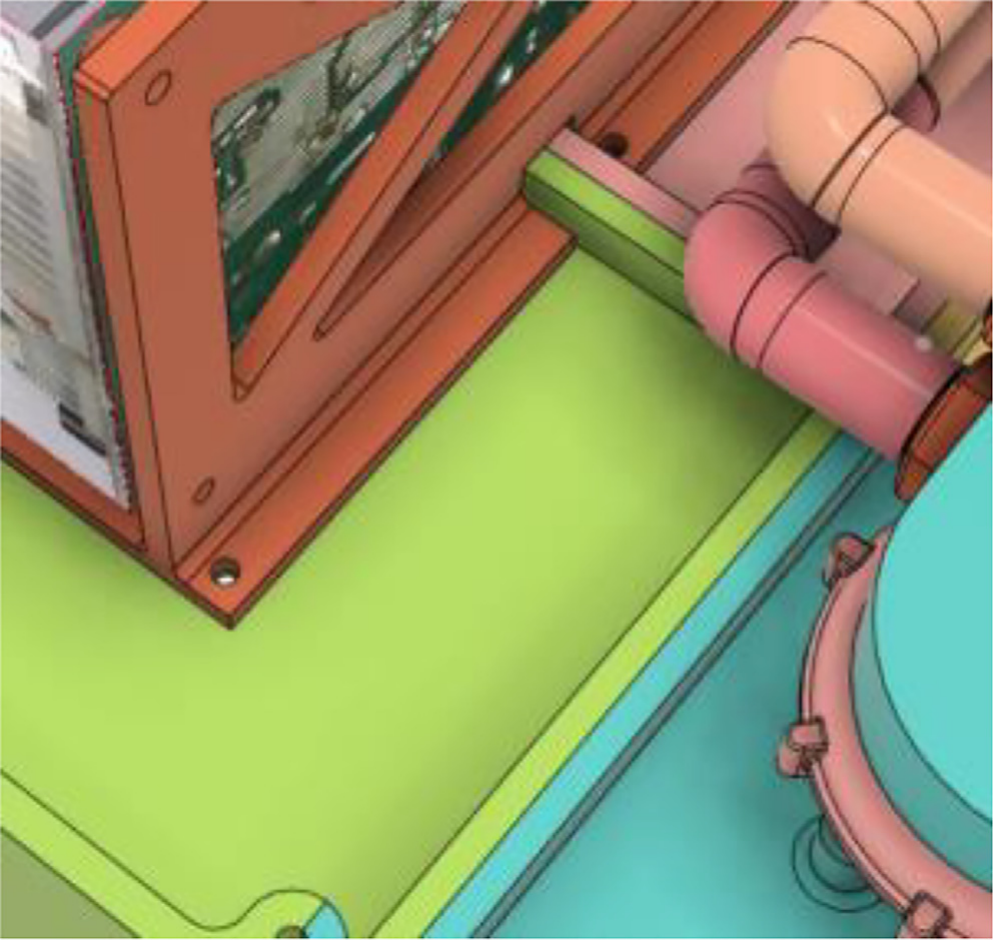
Before gluing the Battery Case (Holder) with the Casing Base (22).

**Fig. 120:**
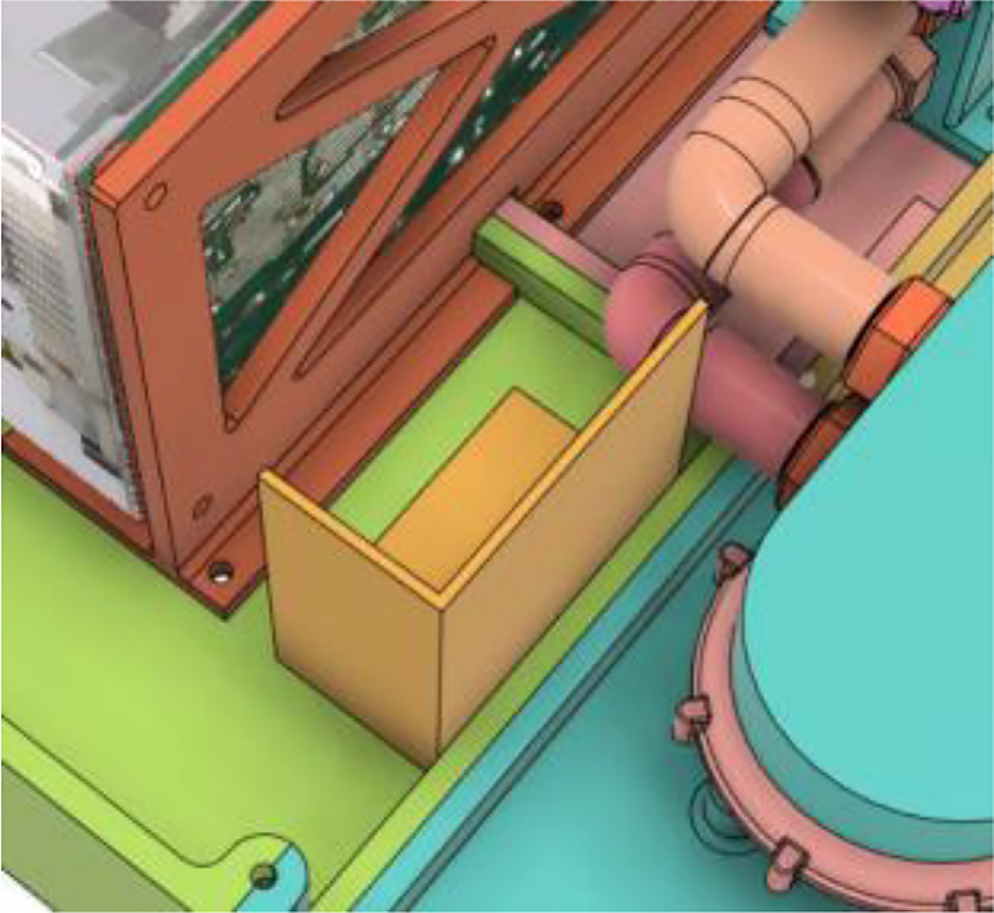
After gluing the Battery Case (Holder) with the Casing Base (22).

**Fig. 121:**
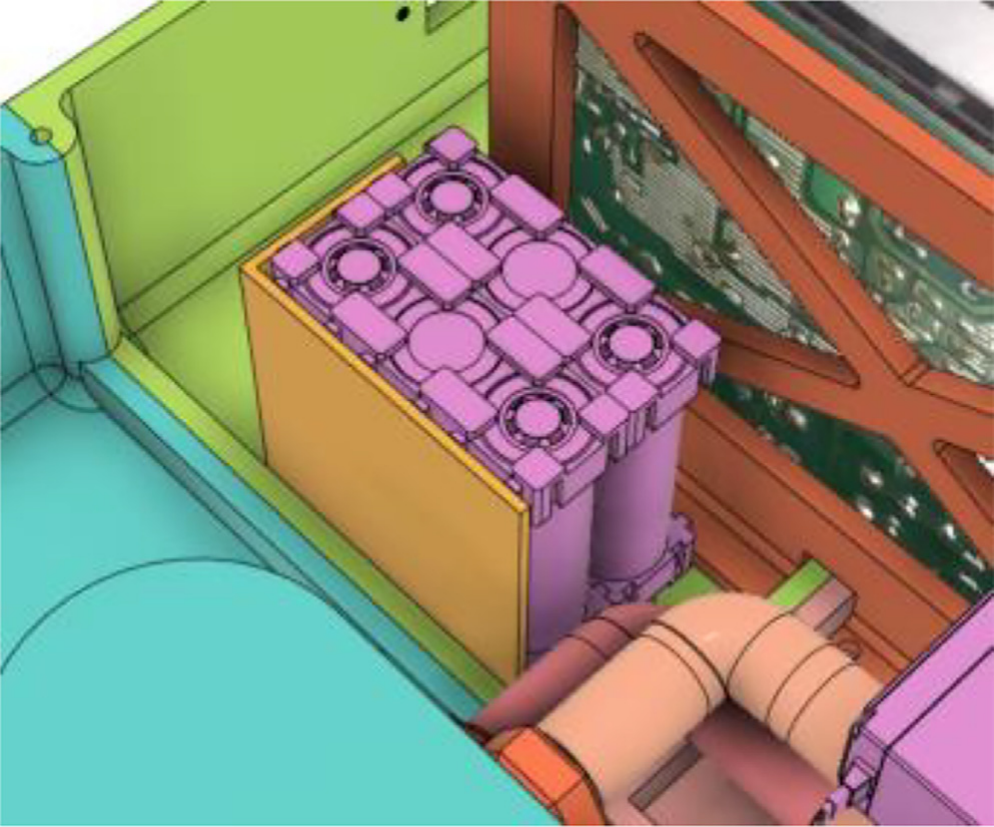
After securing the battery pack with the Battery Case (Holder).
7. Casing Base with installed components (Fig. 122).

**Fig. 122:**
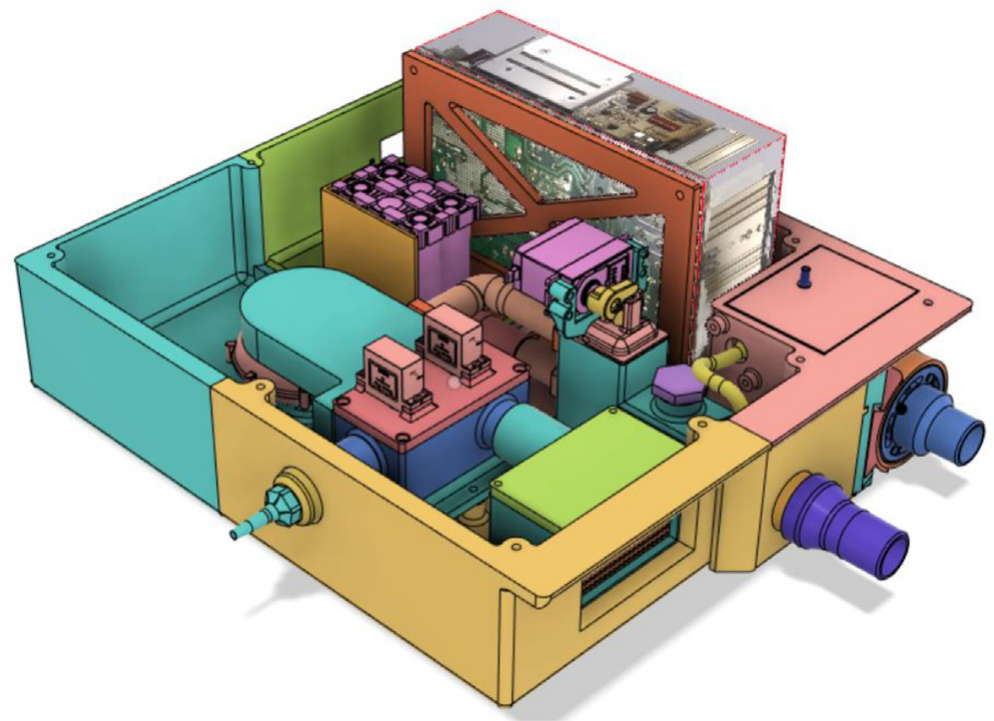
Casing Base with installed components.

### Final assembled ventilator

**Fig. 123:**
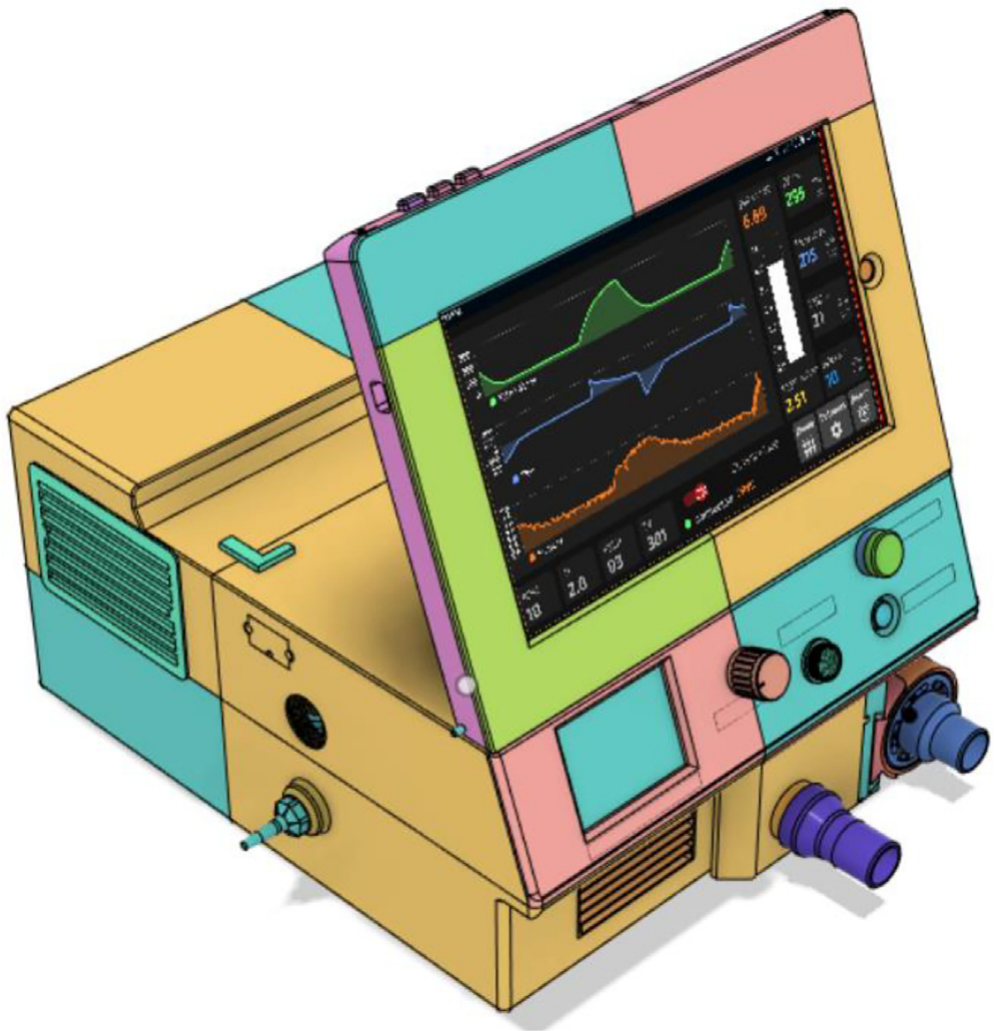
Front View 1.

**Fig. 124:**
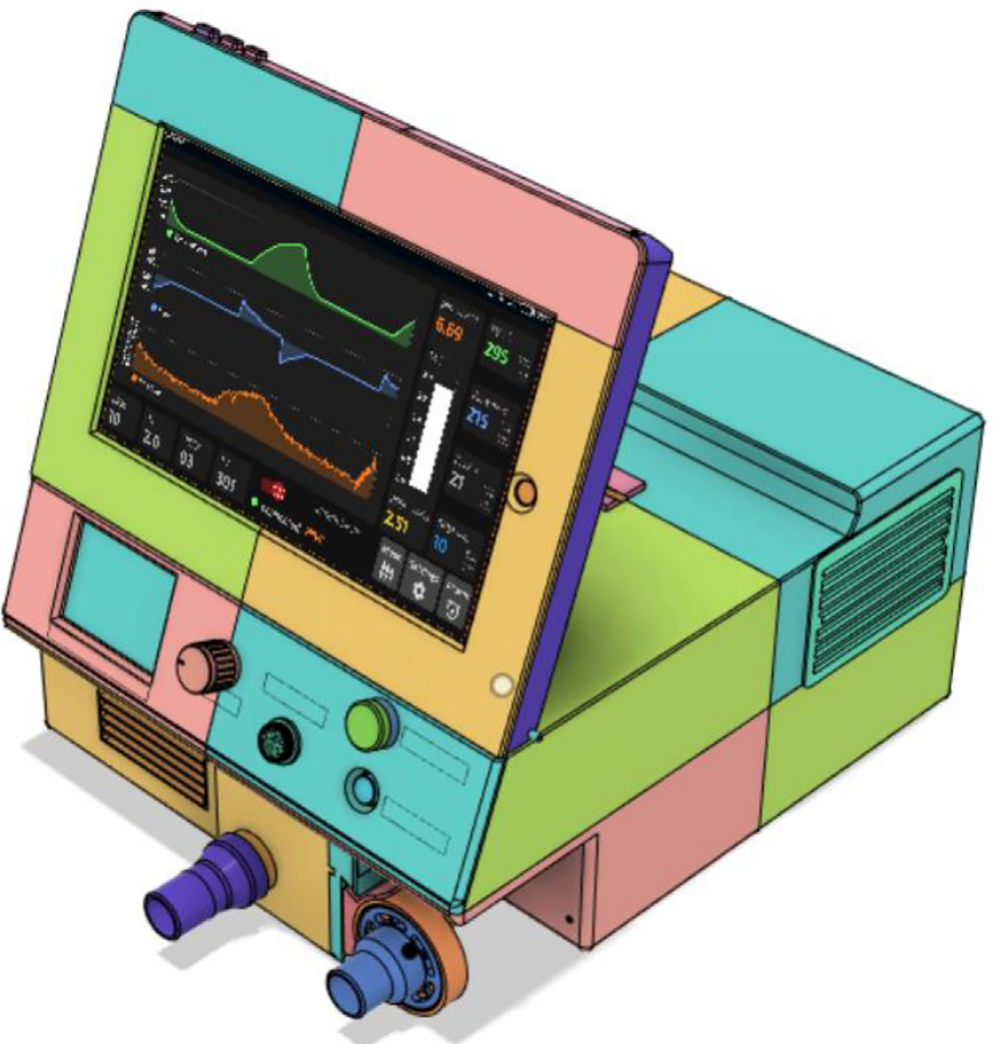
Front View 2.

**Fig. 125:**
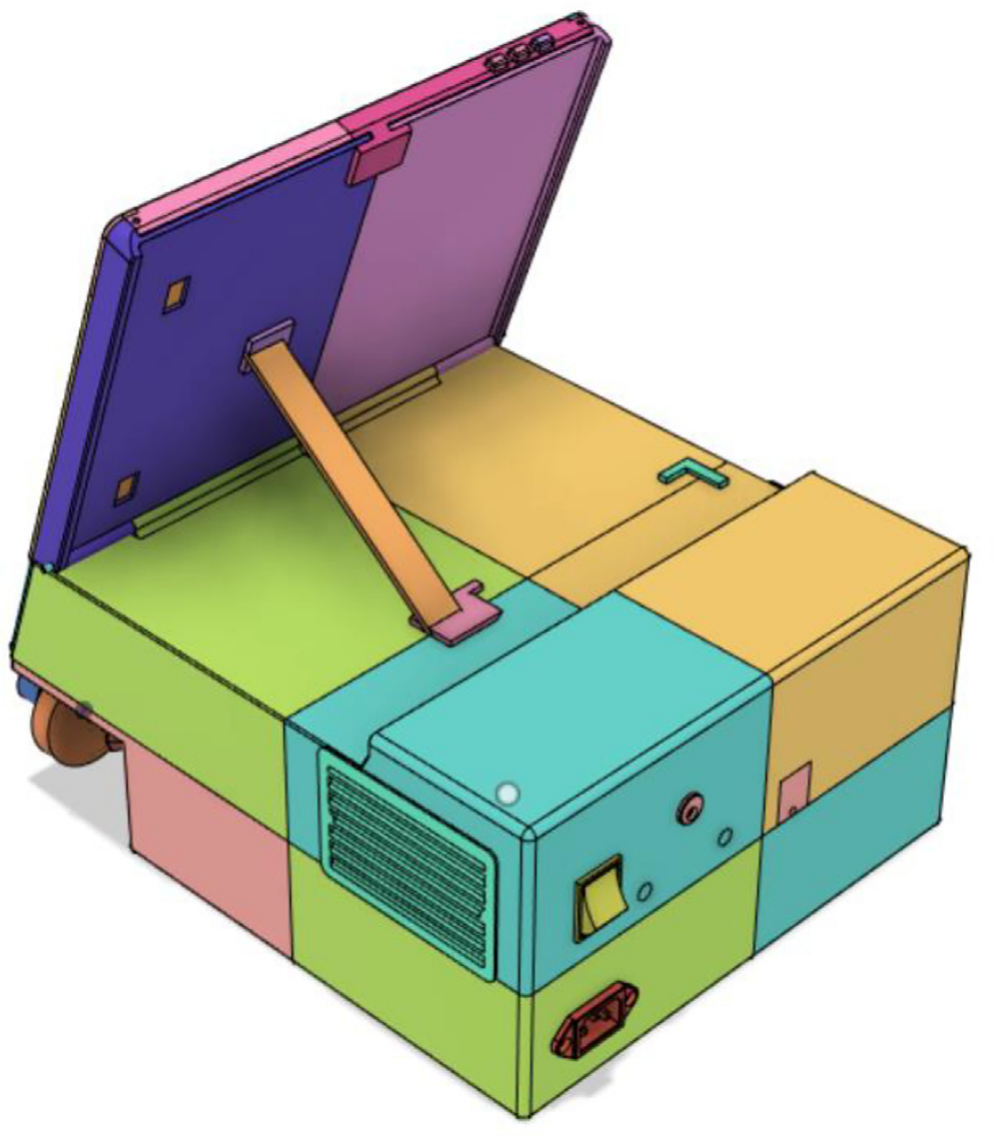
Rear View 1.

**Fig. 126:**
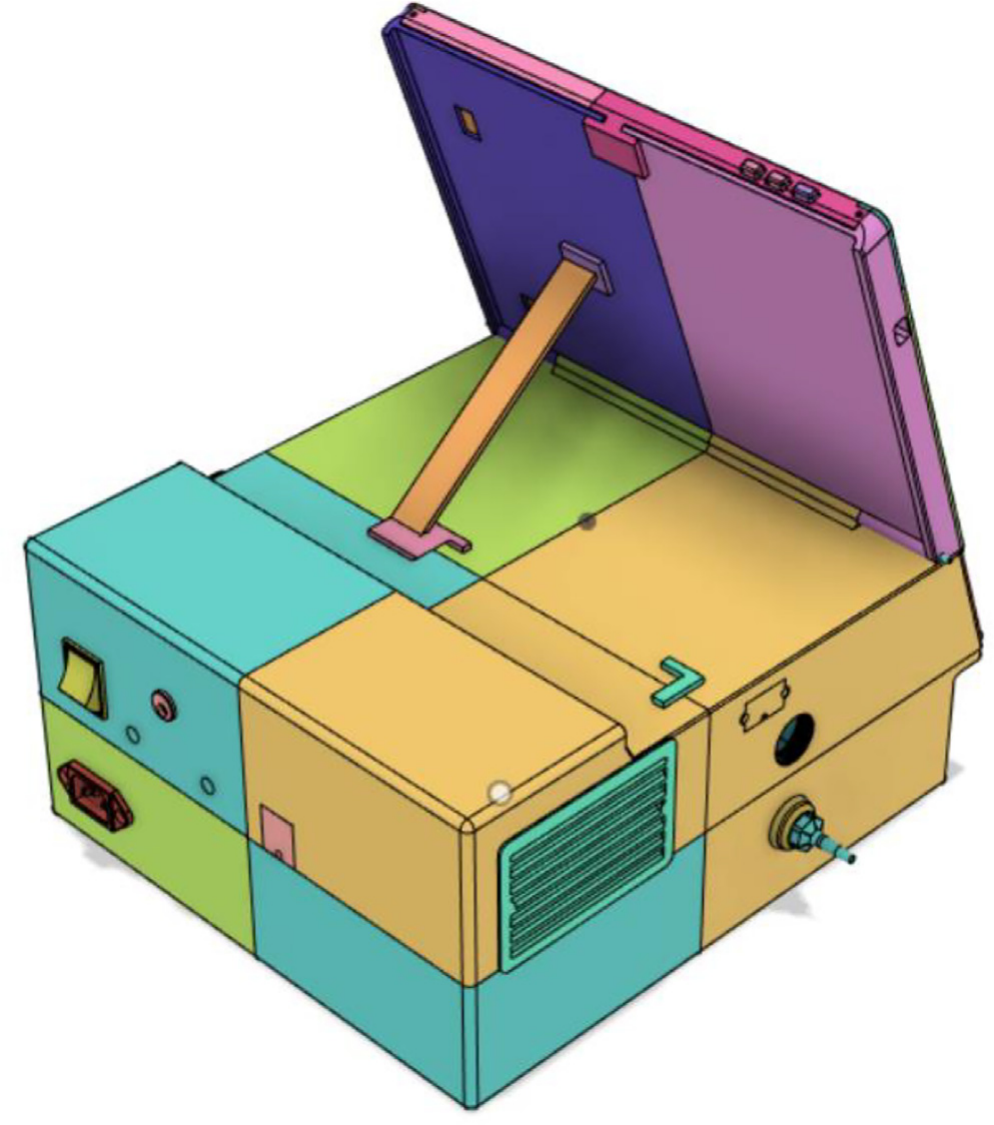
Rear View 2.

(see Fig. 123, Fig. 124, Fig. 125, Fig. 126)

3D Parts Setup:

1. Open the ‘Assembly 3D Model Files’ and find the ‘Ventilator Assembly.step’ file.
2. Open the ‘Ventilator Assembly.step’ file on Fusion360 or any other 3D modeling software that can open this file.
3. Create STL files for each of the parts in this file, which are also mentioned in the ‘Design Files’ section.
4. Open the STL files in slicing software and upload them on a 3D printer for printing the parts.
5. Follow the slicing instructions given in this section:

**Table:** 

Printer: Anycubic i3 Mega Nozzle Diameter: 0.4 mm Filament Material: ABS Filament Diameter: 1.75 mm Slicing Software: Ultimaker Cura Layer Height: 0.2 mm Line Width: 0.4 mm Wall Thickness: 1.2 mm	Top/Bottom Thickness: 1.2 mm Infill density: 50 % Infill Pattern: Zig Zag Nozzle Temperature: 240C Bed Temperature: 100C Print Speed: 40 mm/s Infill Speed: 60 mm/s Support Speed: 60 mm/s Generate Support: Yes Support Placement: Everywhere Support Overhang Angle: 45 Degree

6. After the parts are all printed, follow the detailed assembly instruction provided in the ‘Build Instructions’ section above.

Electronics Setup:

1. Get all the modules as demonstrated through Fig. 11.
2. Connect the modules accordingly using good low resistive wires.

Software Setup.

1. Use Android Studio software to generate the apk files.
2. Install into the android device. All files can be found in the “Android Application” zipped folder.

Firmware Setup:

1. Download all the codes from the ‘Firmware’ folder
2. Connect an Arduino development board
3. Upload the code onto it

Ventilator Specification:

Summarized specifications for the ventilator are demonstrated in Table 3.

**Table 3:** Ventilator specification.

Tidal Volume	100 to 999 ml*
PIP	0 to 35 cm H2O*
BPM	6 to 60*
PEEP	0 to 12 cm H2O*
Modes	PRVC, PCV/ BiPAP (SIMV available for both)
Alarms	TV, PIP, PEEP, Disconnection of the patient circuit, Flow sensor electrical disconnection
Power Supply	13.6 V to 24 V (20–30 W), 100 V − 240 V AC

*Limits restricted through GUI software but hardware and firmware capacity area much higher.

Safety Concerns:

• Under continuous operation for a few years, some of the mechanical and 3D printed parts would need to be changed.
• Prolonged use of filters (HEPA and HME) might decrease the device’s performance.

Operation Instructions:

• Power ON the ventilator.
• Connect the Pressure and Flow sensor with the ventilator.
• Connect the Patient circuit and make sure the One-way valve and the PEEP valve are in the right position.
• Turn ON the physical power switch of the ventilator.
• The pressure sensor will be calibrated automatically
• Turn ON the Android Tab/Phone
• Open “CRUX Ventilator Android App”
• Connect the Ventilator
• From the app, the screen selects the desired parameters. e.g., Mode, Pressure, Tidal Volume, Trigger Flow, etc.)
• From the right bottom corner of the app select Alarm and set desired parameters.
• From the settings option, you can turn ON/OFF debug data (which will be shown in the top left corner)
• In the bottom middle part, there is an ON/OFF switch. Press ON to start ventilation.
• Press the same switch off and a confirmation will be shown. To stop ventilation, press OK.

## Validation and characterization

The device was tested in a couple of ways and effective results were achieved which were verified by professional doctors.

(a) Experiment

**Fig. 127:**
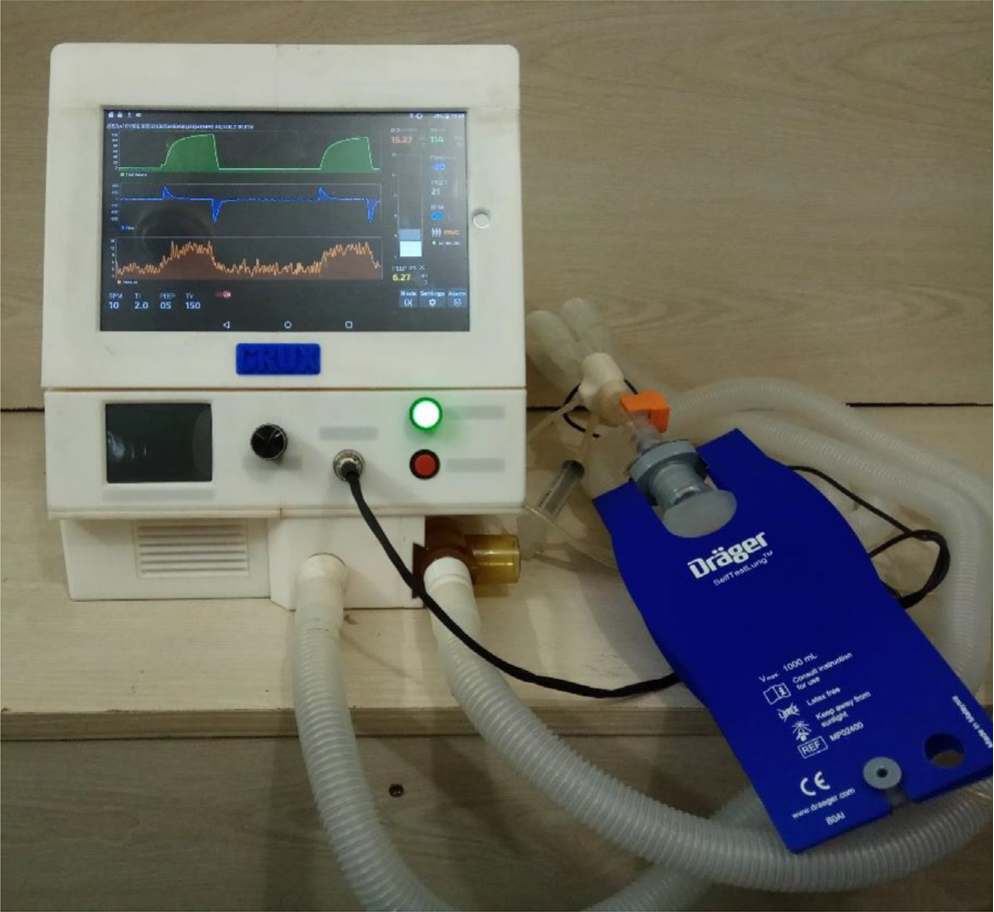
Draeger test lung with flow sensor connected to the patient circuit.

An experimental setup was constructed where the ventilator was connected with a Drager test lung [24] to simulate a patient. The Arduino module of the device was connected to a computer where the data was logged during the operation. The experiment was done on the Draeger test lung [24] to simulate real patient conditions. Fig. 127 demonstrates the test lung with the flow sensor. The Draeger test lung is designed and tested for a lot of Draeger ventilators to be operated in any clinical setting. Innovative carbon fiber, silicone, and polysulfone materials are used in its construction; enough to work with the system while delivering reliable service. This ultra-lightweight test lung merely weighs 190 g (0.42 lb). Besides that, it can be completely disassembled for easy cleaning and is autoclavable [24]. SFM3300 flow sensors [9] were used to verify the magnitude of tidal volume delivered to patients. These sensors are already used in ventilators and other respiratory devices for flow measurements. It is well-known because of its autoclavable feature, capability to measure bi-directional flow rates of 250 slm and small dead space of fewer than 10 ml. Moreover, the biggest advantage of using it is that it has a very fast update time (0.5 ms) and calibration is not needed. Two of such flow sensors were connected in series with the patient circuit through which the air flowed from the turbine to the patient. The first one was connected to the microcontroller directly and data was retrieved using the serial monitor on a computer. The second one was connected to another computer through the official interfacing tool provided by Sensiron EK-F3x-CAP [25].

The analog values from the sensor were verified and converted to cm H_2_O pressure as it is more convenient when it comes to implementing different pressure levels. Besides that, doctors are also used to cm H_2_O pressure. Hence, the analog values were converted using Eq.(11) and they can be found at [13] under SIMV patient trigger condition [Code: volume.ino ≫>≫ 128 no line].

(11) floatmpxCm=(mpxAnalog-mpx2010AnalogOffset)∗C

In the Eq. (11), C has been identified through an experiment. The changes in offset subtracted mpxAnalog has been observed through changing mpxCm. The test data is available in Table 4. Based on the observation, a slope has been calculated to find C. The value of C is thus found to be 0.208879.

**Table 4:** Ventilator specification.

**mpxCm (cm H2O)**		**Offset substracted mpxAnalog**
0		0
1		5
2		10
3		14
4		19
5		24
6		29
7		33
8		38
9		43
10		48
11		53
12		57
13		62
14		67
15		72
16		77
17		81
18		86
19		91
20		96

**Fig. 128:**
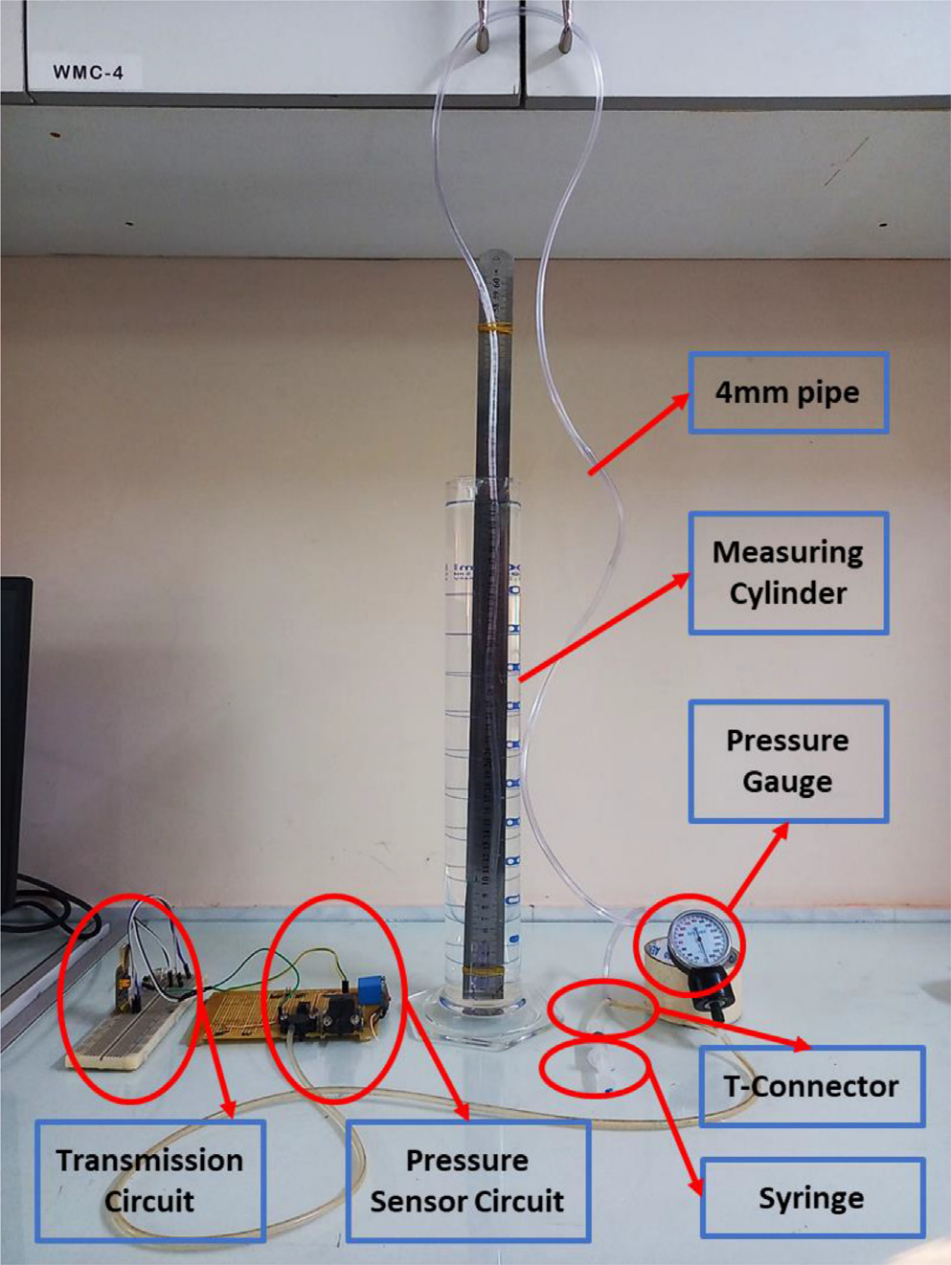
Pressure sensor calibration experimental setup.

Upon not coming across any ready-to-be-used formula for converting an analog value to cm H_2_O, we fabricated our setup in finding out the constant; 0.208879, used in the Eq.(11). The Fig. 128 demonstrates the experimental setup that was used in order to find the constant. The *t*-connector was connected with a syringe on one end and one of the pressure sensing outlets of MPX2010DP, then the third end was connected with a 4 mm pipe and was put into water held by a measuring cylinder. A ruler was held in parallel to the measuring cylinder by its side by a clamp. The height of the water in cm was recorded and then the pressure was exerted by the syringe. When we saw approximately 1 cm H_2_O displacement of water due to pressure exerted by the syringe, the change in the analog value of the differential pressure was recorded. This experiment was repeated a couple of times and the mean value was finally taken to calculate the constant that we are finally using in the formula.

(b) Results

**Fig. 129:**
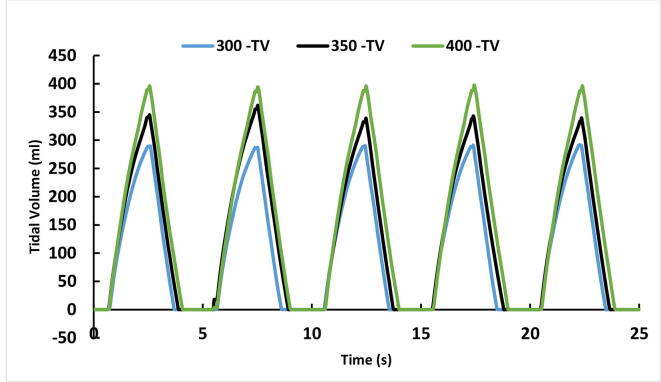
Test results at three tidal volume settings (300, 350 and 400 ml).

**Fig. 130:**
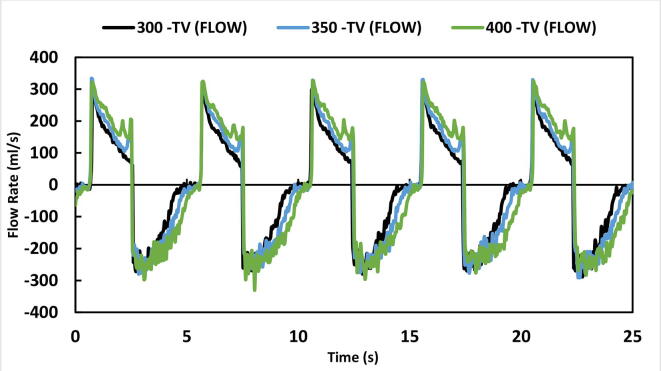
Test results of change in flow rates (ml/s) at three tidal volume settings (300, 350 and 400 ml).

To check its ability to function at different tidal volumes, the device was set at three settings: 300 ml, 350 ml and 400 ml. BPM was set at 12, inspiration time (Ti) was 2.0 Seconds and PEEP was at 4 cmH_2_O. Based on the above parameter settings the flow and volume curves of the mechanical ventilation system are shown below. Fig. 129 represents five breath cycles at the three different tidal volume settings whereas Fig. 130 represents the Flow rates (ml/s) at the same settings. Both graphs have shown expected results.

**Fig. 131:**
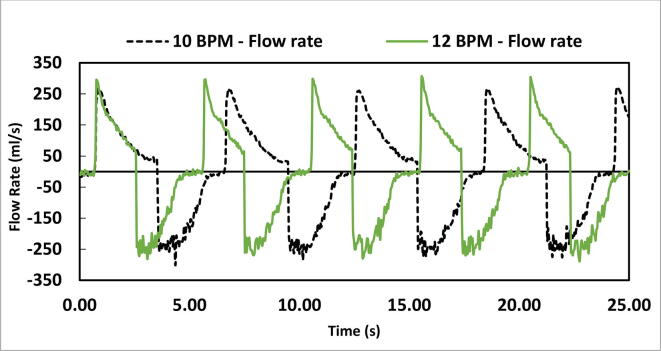
Test results at two BPM settings (10 and 12).

To check its ability to function at different BPM, the device was set at two settings: 10 and 12 BPM. Tidal volume was set at 350 ml, inspiration time (Ti) was 3.0 Seconds and PEEP was at 3 cmH_2_O. Fig. 131 represents five breath cycles at the two BPM settings.

**Fig. 132:**
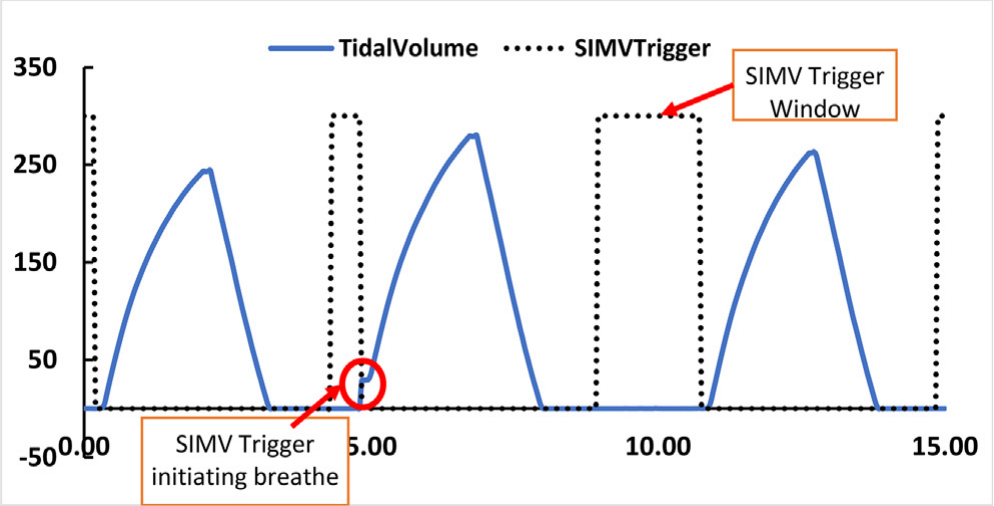
SIMV trigger in the trigger window.

To demonstrate that the system’s SIMV mode is functional, a test was conducted where the test lung was manually pulled to simulate the patient’s breath initiation. Fig. 132 illustrates one such event where SIMV was triggered and a breath was subsequently initiated in the trigger window. Tidal volume was set at 300 ml, inspiration time (Ti) was 2.0 Seconds and PEEP was at 3 cmH2O. SIMV trigger window was deliberately set at 40 % instead of 10 % as a larger window was necessary to cause the trigger by manually pulling the test lung.

(c) Visualization on an integrated display module:

Efficiency is calculated using the values obtained through two methods; J7-t power tested [26] and DPS5005 power supply [27].

J7-t tester has very low power consumption (20 mA current consumption) by itself while displaying the values. Displayed voltages and currents have an accuracy of 0.03 V and 0.02A respectively; with comparatively higher accuracy [26].

DPS5005 power supply has a resolution of 0.01 for both voltages and currents measured with 100 mV (peak to peak) at max workload [27].

**Fig. 133:**
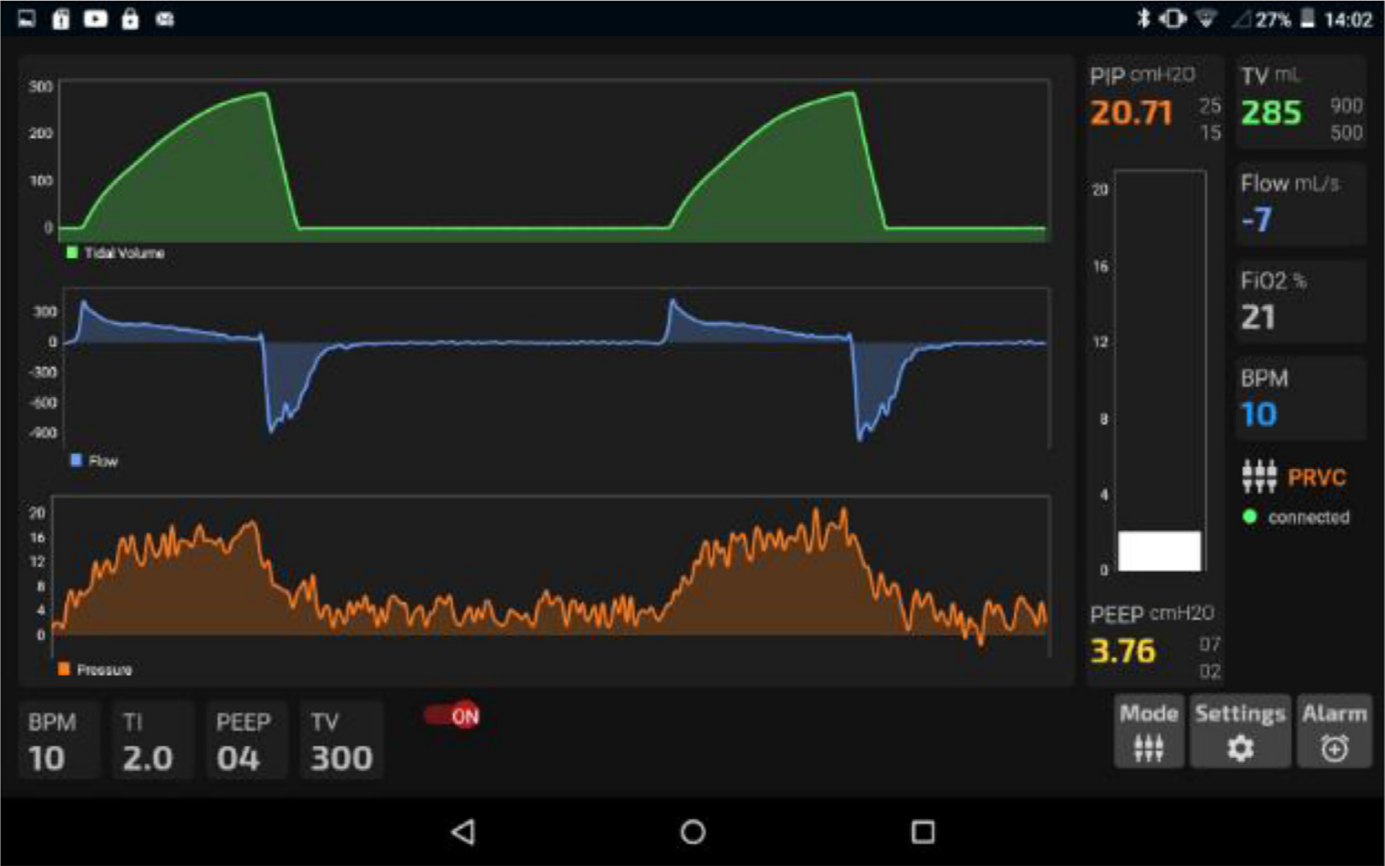
Screenshot of PRVC mode’s GUI.

**Fig. 134:**
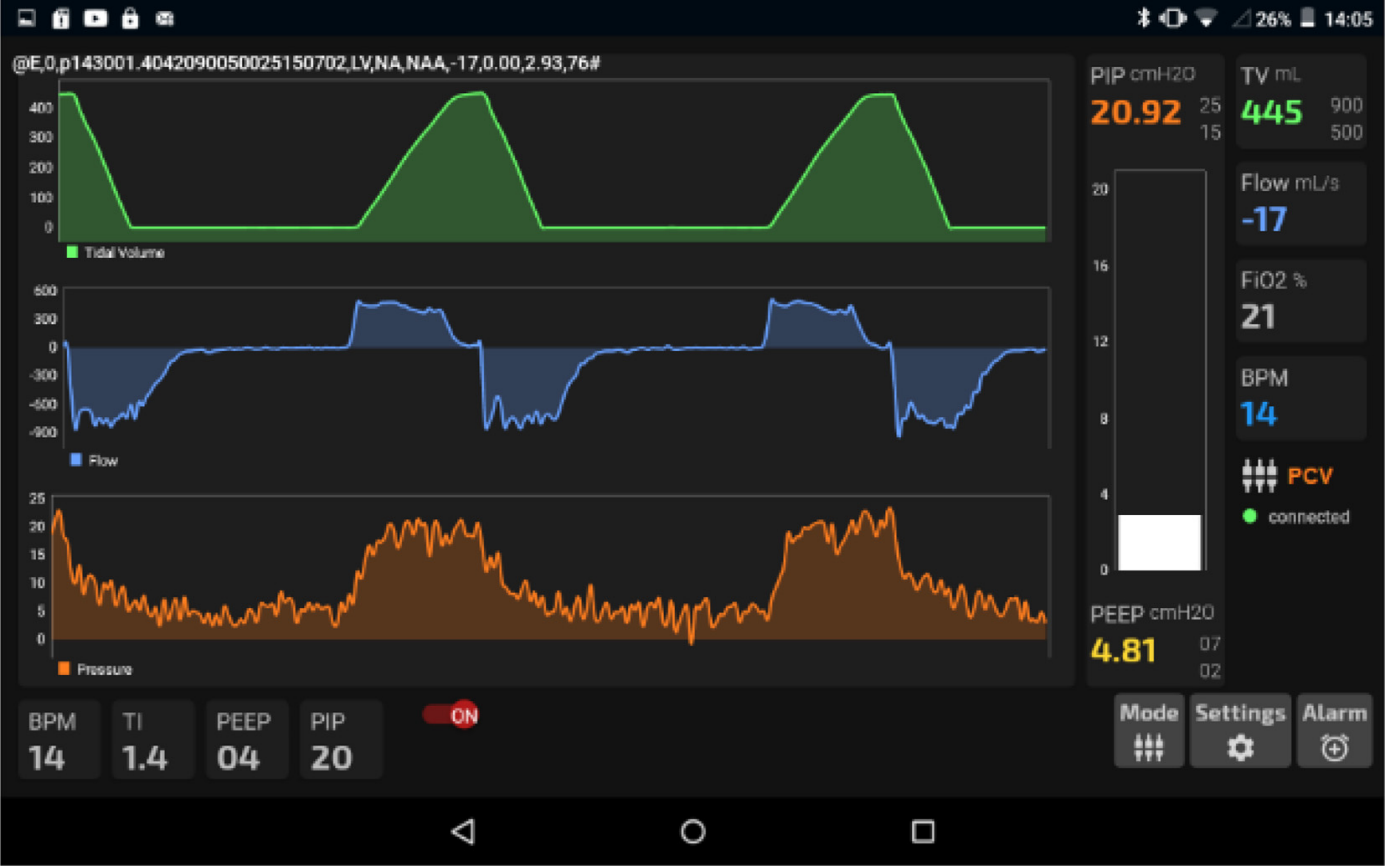
Screenshot of PCV mode’s GUI.

Finally, graphs were obtained and Fig. 133 and Fig. 134 showing the operation of the device in PRVC and PCV modes respectively.

(d) Demonstration.

A complete video demonstration of the proposed ventilator can be found in the following link: https://youtu.be/Mzp99ZKSrOA. In this video you will be able to see the device at work while tested with the test lung. Furthermore, it contains a demonstration of the graphical user interface.

**Financial Support:** This research did not receive any specific grant from funding agencies in the public, commercial, or not-for-profit sectors.

## Conclusion

Technology is always developing around the world but there is still some gap in the implementation of intensive care medical devices in finding ways to reduce cost while keeping the functionalities the same if not better. We have successfully developed a power-efficient system with two completely novel designs; PRM and PEEP valves. These valves have enabled the system to achieve more than 35 times efficiency running alongside effective control algorithms such as feed-forward and PID. The modular low cost and portable system can easily be deployed.

However, we do acknowledge that there are still some developments to be done and these are already in the process; such as, more modes are to be developed, the oxygen blending method needs to be corrected and finally the primary display system needs to be developed with a simpler display that would be able to provide minimal and crucial information so that this system can even run at the scenario where an android tablet is not available.

## Future work

Ventilator testers are not commonly available in LMICs. However, the system can be tested further with an aid of a ventilator tester.

1. Technological changes – More powerful microcontroller > STM32, Digital pressure sensor instead of analog sensor, more robust and integrated power supply.
2. Miniaturization of the design – Designing PCB.
3. FIO2 Control to be added.
4. Heated tube can be added.
5. Instead of using Android tab, a built-in HMI can be used.
6. Battery capacity can be increased.
7. More ventilation modes can be added.
8. More intuitive user interface can be developed.

## Declaration of Competing Interest

The authors declare that they have no known competing financial interests or personal relationships that could have appeared to influence the work reported in this paper.

## References

[1] H. Staff, *Continuous Positive Airway Pressure (CPAP) Therapy for Obstructive Sleep Apnea | Michigan Medicine*. Uofmhealth.org. Retrieved 5 September 2021, from https://www.uofmhealth.org/health-library/hw48752, 2020.

[2] MPX2010 Series. *Nxp.com*. Retrieved 5 June 2021, from https://www.nxp.com/docs/en/data-sheet/MPX2010.pdf.

[3] Team, R. *Raspberry Pi 4 Tech Specs*. Raspberrypi.org. Retrieved 5 September 2021, from https://www.raspberrypi.org/products/raspberry-pi-4-model-b/specifications/.

[4] O. Hardware, *Radxa Wiki*. Wiki.radxa.com. Retrieved 5 September 2021, from https://wiki.radxa.com/News/2014/10/rock-pro-lite-is-oshw-now, 2014.

[5] Kristiawan R., Imaduddin F., Ariawan D., Ubaidillah, Arifin Z. (2021). A review on the fused deposition modeling (FDM) 3D printing: Filament processing, materials, and printing parameters. Open Engineering.

[6] *Filter/ HME*. *Draeger*. Retrieved 5 June 2021, from https://www.draeger.com/en_seeur/Products/Filter-and-Heat-and-moisture-exchanger.

[7] *Ventilator centrifugal blower fan DC12V*. *aliexpress.com*. Retrieved 5 June 2021, from https://www.aliexpress.com/item/10000048326249.html?spm=a2g0s.8937460.0.0.438c2e0ePyDUwe.

[8] Acosta P., Santisbon E., Varon J. (2007). The Use of Positive End-Expiratory Pressure in Mechanical Ventilation. Critical Care Clinics.

[9] *Digital Flow Meter for medical applications*. Mouser.com. Retrieved 5 June 2021, from https://www.mouser.com/datasheet/2/682/Sensirion_Mass_Flow_Meters_SFM3300_Datasheet-1524535.pdf.

[10] *Arduino Mega 2560 Rev3 | Arduino Official Store*. Store.arduino.cc*.* Retrieved 14 June 2021, from https://store.arduino.cc/usa/mega-2560-r3.

[11] *Bluetooth Module HC-05 | Sensors & Modules*. Electronicwings.com. Retrieved 7 June 2021, from https://www.electronicwings.com/sensors-modules/bluetooth-module-hc-05-.

[12] *MG995 Servo Motor*. Components101. Retrieved 5 June 2021, from https://components101.com/motors/mg995-servo-motor.

[13] S. Nabil, *Nabilphysics/ventilator*. GitHub. Retrieved 26 August 2021, from https://github.com/Nabilphysics/ventilator/blob/main/Firmware/volume.ino, 2021.

[14] *T Connector – TARSONS*. Tarsons.com. Retrieved 7 June 2021, from https://tarsons.com/product/t-connector/.

[15] *Lithium Ion NCR18650B.* Batteryspace.com. Retrieved 17 June 2021, from https://www.batteryspace.com/prod-specs/NCR18650B.pdf.

[16] *Practical differences between pressure and volume controlled ventilation | Deranged Physiology.* Derangedphysiology.com. (2021). Retrieved 4 September 2021, from https://derangedphysiology.com/main/cicm-primary-exam/required-reading/respiratory-system/Chapter%20542/practical-differences-between-pressure-and-volume-controlled-ventilation.

[17] *Pressure Control Ventilation | WEINMANN.* Weinmann-emergency.com. (2021). Retrieved 4 September 2021, from https://www.weinmann-emergency.com/solutions/ventilation-modes/pressure-control/.

[18] *Multithreading in Java - javatpoint*. www.javatpoint.com. Retrieved 4 September 2021,from https://www.javatpoint.com/multithreading-in-java.

[19] *Asynchronous | What is Asynchronous - javatpoint*. www.javatpoint.com. Retrieved 4 September 2021, from https://www.javatpoint.com/asynchronous.

[20] *Handler | Android Developers.* Android Developers. Retrieved 4 September 2021, from https://developer.android.com/reference/android/os/Handler.

[21] *BluetoothSocket | Android Developers*. Android Developers. Retrieved 5 September 2021, from https://developer.android.com/reference/android/bluetooth/BluetoothSocket.

[22] *string::substr - C++ Reference*. Cplusplus.com. Retrieved 5 September 2021, from https://www.cplusplus.com/reference/string/string/substr/.

[23] Chatburn R., El-Khatib M., Mireles-Cabodevila E. (2014). A Taxonomy for Mechanical Ventilation: 10 Fundamental Maxims. Respiratory Care.

[24] Team, D. *Dräger SelfTestLung™ Consumables and Accessories*. Draeger.com. Retrieved 5 September 2021, from https://www.draeger.com/Products/Content/selftestlung-pi-9068491-en.pdf.

[25] *Quick Start Guide EK-F3x-CAP*. Sensirion.com. Retrieved 5 September 2021, from https://www.sensirion.com/fileadmin/user_upload/customers/sensirion/Dokumente/5_Mass_Flow_Meters/Sensirion_Mass_Flow_Meters_EK-F3x-CAP_Quick_Start_Kit_EN.pdf.

[26] *Test of USB safety tester J7-t*. Lygte-info.dk. Retrieved 5 September 2021, from https://lygte-info.dk/review/USBmeter%20safety%20tester%20J7-t%20UK.html.

[27] *DPS5005 - Joy-IT*. Joy-it.net. Retrieved 5 September 2021, from https://joy-it.net/en/products/JT-DPS5005.

